# Terahertz Spectroscopic Analysis of Lactose in Infant Formula: Implications for Detection and Quantification

**DOI:** 10.3390/molecules27155040

**Published:** 2022-08-08

**Authors:** Sopanant Datta, Kiattiwut Prasertsuk, Nuttawat Khammata, Patharakorn Rattanawan, Jia Yi Chia, Rungroj Jintamethasawat, Thawatchart Chulapakorn, Taweetham Limpanuparb

**Affiliations:** 1 Science Division, Mahidol University International College, Mahidol University, Salaya, Phutthamonthon, Nakhon Pathom 73170, Thailand; 2 National Electronics and Computer Technology, National Science and Technology Development Agency, 112 Thailand Science Park, Khlong Luang 12120, Thailand; 3 Department of Physics and Materials Science, Faculty of Science, Chiang Mai University, Chiang Mai 50200, Thailand; 4 Department of Construction Sciences, Lund University, 22100 Lund, Sweden

**Keywords:** α-lactose monohydrate, Terahertz time–domain spectroscopy, lactose quantification

## Abstract

Lactose plays a significant role in daily lives as a constituent of various food and pharmaceutical products. Yet, lactose intolerance conditions demand low-lactose and lactose-free products in the market. These increasing nutritional claims and labels on food products entail simple and reliable methods of analysis that can be used for meeting quality standards, nutritional claims and legal requirements. In this study, terahertz time–domain spectroscopy (THz-TDS) was employed to analyse α-lactose monohydrate qualitatively and quantitatively in food products. Both absorption spectra and absorption coefficient spectra were investigated for their prediction performance. Regression models for lactose quantification using peak area and height of the absorption peaks 0.53 and 1.37 THz were developed and assessed in infant formula samples. Satisfactory prediction results were achieved in ideal conditions with pure standards, but not in all predictions of infant formula samples. Reasons and further implications are discussed.

## 1. Introduction

Amongst various populations, milk and milk products are considered as integral parts of the diet. Over 6 billion people worldwide consume dairy products [1]. Milk is a good source of protein, calcium, phosphorus, magnesium and other essential macro- and micronutrients. The disaccharide lactose is the principal sugar in milk, which can be broken down into its monomers glucose and galactose by the enzyme lactase in our gastrointestinal tract [2]. Lactose intolerance is a common condition in individuals with lactase deficiency, causing symptoms such as flatulence and diarrhoea [3]. This condition is generally treated with the avoidance of lactose-containing food products. To meet the dietary requirements of the lactose intolerant population, various low-lactose and lactose-free dairy products have been developed and sold in the market.

In addition to being found naturally in milk, lactose has important applications in the food industry. It is added to infant formulas to imitate the lactose content in human milk [2]. With its lack of flavour and low sweetness, it is used as a bulking agent that does not significantly add to the overall sweetness, a diluent or enhancer of flavour and a reducing sugar in the Maillard reaction to form flavour and colour compounds [4].

In the pharmaceutical industry, lactose is a widely used excipient of drugs. It is generally used as a filler and binder in oral solid dosage formulations, such as tablets and dry powder inhalers [5]. The most common form of lactose used as an excipient is crystalline α-lactose monohydrate [6].

The detection and quantification of lactose are vital in the food and pharmaceutical industries for product research and development, quality control, nutritional labelling and verification of nutritional claims. In these processes, it is necessary to handle appropriate equipment for the rapid, simple and real-time determination of lactose content in food and pharmaceutical products.

Commonly used and recognised analytical methods for lactose determination include spectrophotometric [AOAC 2006.06], spectroscopic [AOAC 972.16], polarimetric [AOAC 896.01], gravimetric [AOAC 930.28] and chromatographic methods. Conventional methods are modified and novel methods are developed to improve the sensitivity, selectivity and limit of detection [7]. These improvements can be achieved with chromatographic and spectroscopic methods (Table 1) such as the conventional high-performance liquid chromatography (HPLC) [8], hydrophilic interaction chromatography (HILIC) [9], high-performance thin layer chromatography (HPTLC) [10], high-performance anion exchange chromatography with pulsed amperometric detection (HPAEC-PAD) [7], near-infrared spectroscopy (NIR) [11] and nuclear magnetic resonance (NMR) [12]. In spite of the sensitivity, selectivity and accuracy achieved with these methods, they are limited by their high requirements for operation cost, time consumption and operator expertise [13]. These limitations can introduce difficulties in the routine analysis of samples to meet quality standards and labelling requirements.

Recently, terahertz (THz) spectroscopy has been demonstrated to have significant potential as a rapid, non-destructive and reliable method of analysis in the food and pharmaceutical industries. Terahertz waves, with a frequency range between the microwave and infrared regions (0.1–10 THz), are non-ionising, have high penetrability and can provide intermolecular information on vibrational activities of molecules. They can detect weak intermolecular interactions such as hydrogen bonds and van der Waals forces [14]. Terahertz time–domain spectroscopy (THz-TDS) has been used for the detection and identification of pesticides [15], microplastics [16], amino acids and sugars including lactose [11,17,18,19]. As this method measures crystal lattice vibrations, it detects crystalline forms of molecules [11,20,21]. Therefore, it can be used as a preliminary method of detection and quantification or used to measure the crystallinity of compounds in food and pharmaceutical samples.

In this study, THz-TDS is employed to detect and quantify α-lactose monohydrate in food samples. A quantitative model for lactose analysis is developed using two different well-known absorption peaks at 0.53 and 1.37 THz [22]. The resulting model is applied to dairy product samples to assess THz-TDS as a potential rapid and real-time method of the detection and quantification of compounds in food.

## 2. Results and Discussion

### 2.1. Spectra of Pure Samples

**Figure 1:**
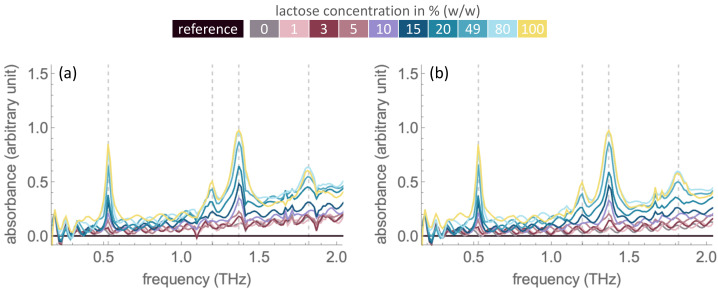
THz absorption spectra of α-lactose monohydrate at varying concentrations under (**a**) ambient condition (≈37% RH) and (**b**) nitrogen-rich (≤7% RH) atmosphere. Frequencies of absorption peaks (0.53 THz, 1.20 THz, 1.37 THz, 1.82 THz) are signified by vertical dashed lines. Absorbance values (arbitrary unit) are the ratio between the intensity of the substance and the reference, as described in Equation (1) under Section 3.

THz-TDS measurements were conducted with pure α-lactose monohydrate samples at concentrations of 0%, 1%, 3%, 5%, 10%, 15%, 20%, 49%, 80% and 100% (*w*/*w*). The spectra are in the range of 0.3–2.0 THz with a high signal-to-noise ratio. Measurements were conducted under ambient conditions (≈37% RH) and a nitrogen-purged atmosphere (≤7% RH) to explore the effects of moisture. Figure 1 shows the absorption spectra from measurements under ambient conditions and one of the replicate measurements in a nitrogen atmosphere. The absorption peaks observed are at the frequencies of 0.53, 1.20, 1.37 and 1.82 THz, in agreement with previous studies using THz-TDS [20,21,22,23,24,25] and density functional theory simulation [24]. The absorption peaks obtained from measurements in a nitrogen atmosphere and those obtained under ambient conditions are of the same frequency and magnitude, indicating that the lactose absorption peaks are independent of water absorption. 

Amongst the absorption peaks, strong peaks are observed at 0.53 THz and 1.37 THz, with clear detection at concentrations of 10% (*w*/*w*) and higher. The absorbance value of an absorption peak varies with its corresponding lactose concentration. That is, absorption peaks in spectra of higher lactose concentrations are higher in absorbance. Nevertheless, the etalon feature or fringe pattern are present in the frequency domain spectra of all samples. This etalon feature arises as the transmitted THz radiation propagates through the air/sample and sample/air interfaces. This effect is more pronounced in pellets of a low thickness (≈1 mm), as used in this study [26]. The etalon pattern leads to difficulties in baseline removal, an increased limit of detection, and inaccuracies in the quantitative analysis.

**Figure 2:**
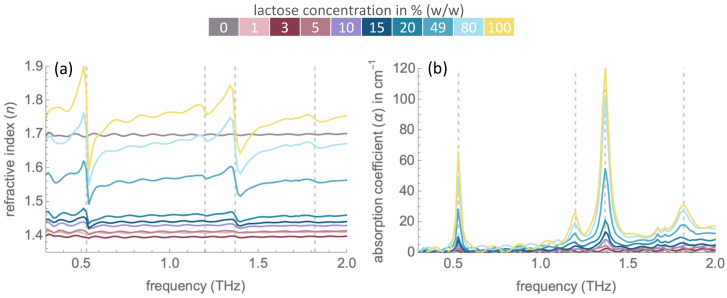
(**a**) THz refractive index (*n*) and (**b**) absorption coefficient (α in cm^−1^) spectra of α-lactose monohydrate at varying concentrations under nitrogen-rich atmosphere. Frequencies of absorption peaks (0.53 THz, 1.20 THz, 1.37 THz, 1.82 THz) are signified by vertical dashed lines. The calculation of refractive index and absorption coefficient are described in Equations (2) and (3) under Section 3.

Spectral artefacts induced by the etalon effect are not easily removed by ratioing to a reference spectrum. One of the techniques of etalon effect elimination is the use of a reference spectrum obtained from a combination of thicknesses and the refractive index of the sample [26]. Thus, absorption coefficient spectra are investigated in this study, as the spectra involve more parameters including the correction for sample thickness (see calculation methods in Section 3). The refractive index and absorption coefficient spectra of pure lactose samples are shown in Figure 2. In comparison to Figure 1, the etalon effect in the absorption coefficient spectra is considerably reduced, yet not totally eliminated. Both refractive indices and absorption coefficients increase with the increasing concentration of lactose. However, pronounced transitions can be observed for the lactose concentrations of 5% (*w*/*w*) and higher. In the absorption coefficient spectra, strong absorption peaks centred at 0.53, 1.20, 1.37 and 1.82 THz are observed. Dispersion regions at the same frequencies are observed in the refractive index spectra, whereby those at 1.82 THz are considerably less pronounced, which is in agreement with a previous study [25].

### 2.2. Quantification Model

**Figure 3:**
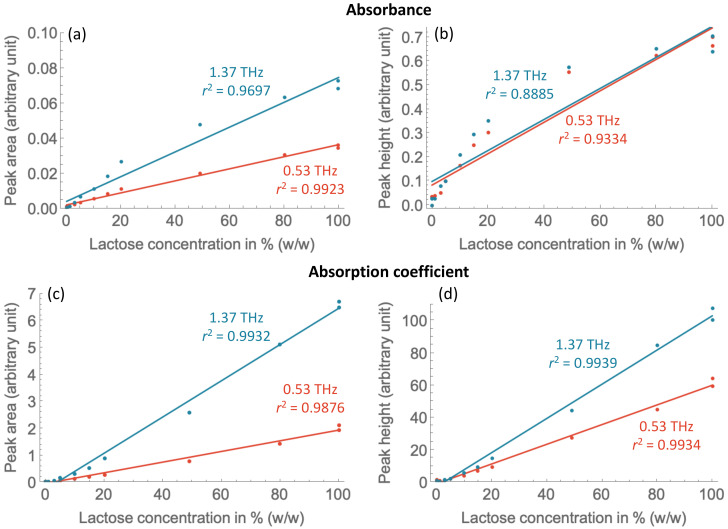
The relationship between α-lactose monohydrate concentration and (**a**) absorbance peak area, (**b**) absorbance peak height, (**c**) absorption coefficient peak area and (**d**) absorption coefficient peak height of the absorption peaks at 0.53 THz and 1.37 THz, from measurements in nitrogen atmosphere. Simple linear regression analysis [19] was applied on the datasets and results are reported in Table 2.

The positive relationship between the α-lactose monohydrate concentration and peak area or peak height of the two major absorption peaks, 0.53 THz and 1.37 THz, were observed as shown in Figure 3. Peaks from the absorption spectra are considered in comparison to those from the absorption coefficient spectra. Gaussian fitting and baseline correction were performed to extract the peak area and peak height values from the spectra. The resulting models and predictions from different datasets (experimental replicates) and baseline correction methods can be found in the Supplementary Materials. A model of lactose quantification, using the absorption and absorption coefficient spectra as predictors, from a measurement performed in a nitrogen atmosphere is shown in Table 2.

For the absorption spectra, the plot of the lactose concentration with the peak area shows that the slope from the 1.37 THz absorption peak is higher than that from the 0.53 THz peak, denoting how lactose vibrational modes are more sensitive to 1.37 THz waves. Peak area of the 0.53 THz absorption peak results in a slightly more accurate prediction of lactose concentration, with *r*^2^ values of 0.9923 and 0.9697 for the 0.53 THz and 1.37 THz absorption peaks, respectively. This is also reflected in the RMSE values shown in Table 2. The better performance of the 0.53 THz peak is consistent with the analysis of a previous study, whereby the peak is found to have a more linear baseline and clear start and end points [27]. The plot of the lactose concentration with the peak height, on the other hand, shows very similar trendlines between the 0.53 THz and 1.37 THz absorption peaks. Similar to the peak area models, the model from the 0.53 THz absorption peak has a higher *r*^2^ value (and lower RMSE value) than the 1.37 THz absorption peak, 0.9334 and 0.8885, respectively. Both models from the peak area had a better prediction performance than those from the peak height, as the peak area takes into account the unequal peak broadenings and peak shape distortions. The result is consistent with a previous study on α-lactose monohydrate, whereby prediction using the peak area of the 0.53 THz absorption peak had a better performance than that using the peak height [19].

For the absorption coefficient spectra, plots of the 1.37 THz show higher slopes than those of 0.53 THz in a similar manner to the absorption spectral data. However, slightly more accurate predictions are achieved with the 1.37 THz peak, in contrast to the results from the absorption spectra. In addition, the peak height of both peaks resulted in higher *r*^2^ values than the peak area.

Comparing the results from the absorption and absorption coefficient spectral data, most models from the absorption coefficient spectra had better prediction accuracy than those from the absorption spectra. The more pronounced etalon effect in the absorption spectra could have led to greater difficulties in the baseline removal. This is responsible for the relatively high limit of detection (see Section 2.1). The use of the absorption coefficient spectra can reduce the etalon effect, thereby improving the limit of detection with high prediction accuracy.

### 2.3. Application on Diary Product Samples

The qualitative and quantitative determination of lactose using THz spectroscopy were performed on three commercial infant formula samples of the same brand. Sample 1 and Sample 2 are normal infant formula products and Sample 3 is a lactose-free product (see product labels in Figures S2–S4, Supplementary Materials). To assess the prediction performance of the quantification model, Sample 1 and Sample 2 were sent to two external accredited laboratories for lactose quantification (using AOAC official methods 980.13 and 982.14) and are used as reference values. 

**Figure 4:**
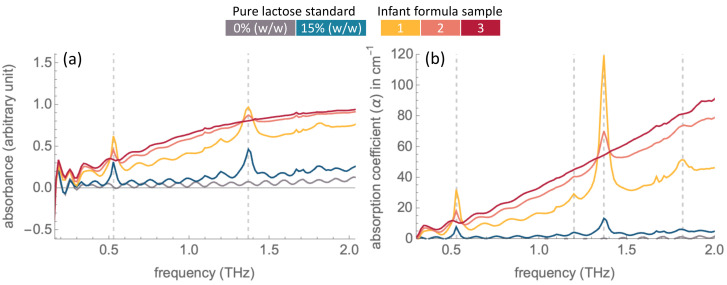
THz (**a**) absorption and (**b**) absorption coefficient spectra of infant formula samples, 15% (*w*/*w*) lactose and 0% (*w*/*w*) lactose standard samples.

The absorption and absorption coefficient spectra obtained from measurement in a nitrogen atmosphere are shown together with pure lactose standard samples (0% and 15% (*w*/*w*) lactose) in Figure 4. Absorption peaks at 0.53 THz and 1.37 THz are clearly observed from the spectra of Sample 1 and Sample 2, signifying the presence of α-lactose monohydrate in these samples. These absorption peaks are not observed in the spectrum of the lactose-free Sample 3.

For the quantification performance, the mean predicted concentration of lactose using the absorption peak area are shown in Table 3. The lactose concentration obtained from the HPLC measurements in the external laboratories and carbohydrate content from the product nutritional labels are shown as a reference. In this table, the predicted concentrations of lactose anhydrous (molar mass = 342.3 g mol^−1^), which is ≈95%, by mass, that of α-lactose monohydrate (molar mass = 360.3 g mol^−1^), are used for comparison to align to the values from the product labels and HPLC.

According to product label and HPLC quantification, Sample 1 and Sample 2 have similar lactose concentrations (~60%). However, THz-TDS results differ significantly (~60% vs. ~20% for Samples 1 and 2, respectively). This discrepancy can be due to the distinctive formulations of the two samples (see Figures S2 and S3, Supplementary Materials), with different sources of lactose and preparation techniques. This can result in lactose of different forms and properties, to which THz-TDS is sensitive [25].

The models from the 0.53 THz peak in the absorption coefficient spectra resulted in a prediction closest to the result from the gold standard method of HPLC in Sample 1. However, the prediction from the other models were far from the HPLC result. The inaccuracy of prediction can be due to multiple reasons. First, there is a significant baseline shift in the spectra of the infant formula samples compared to the spectra of the pure standards (Figure 4), resulting from the infant formula matrix. It has been found that the magnitude of the baseline can be influenced by particle size [27]. The scattering effects in a nonlinear baseline also vary with the sample thickness [26]. Second, to achieve an accurate prediction of compound concentration, the selection of optimum conditions and parameters are necessary. For instance, the linearity of the data was improved with the use of absorption coefficient spectra, resulting in a better prediction performance compared to the other models.

While the prediction in Sample 1 was quite close to the HPLC result, the prediction in Sample 2 differed significantly. This can be attributed to the distinctive formulation and processing methods for the two samples, resulting in the presence of lactose in different forms. Lactose may exist in its amorphous form and crystalline forms of α-lactose monohydrate, α-lactose anhydrous and β-lactose anhydrous. The major form of lactose used in commercial infant formula is α-lactose monohydrate, due to its non-hygroscopicity [28]. However, it may not be at its maximum purity and may undergo mutarotation or conversion to other anomers during storage [28,29]. This can occur depending on storage conditions such as temperature and humidity. While HPLC resulted in similar concentration values between the two samples, THz-TDS results differed considerably due to its sensitivity to distinctive forms of a compound.

For Sample 3, the negative concentration value predicted from the 0.53 THz absorption peak could be due to an improper baseline fitting, as mentioned earlier. The other three predictors resulted in lactose concentrations close to 0% (0 is in the range of mean ± SD) or no detection of lactose, which is in line with the “lactose-free” label.

Overall, THz-TDS can be employed to verify the lactose-free claim in products. However, the accuracy of quantification in lactose-containing products can be influenced by its presence in multiple forms. Pioneer THz-TDS studies on mixtures of pure compounds with the utilisation of nonlinear quantification models were explored by the authors [30]. This may be applicable to lactose in the future.

## 3. Materials and Methods

Analytical grade α-lactose monohydrate and polyethylene (PE) powder of >98% purity were purchased from Sigma-Aldrich. Commonly available infant formula products were bought from a local supermarket. Chemicals and samples were stored in a dry cabinet at room temperature without any pre-treatment.

To obtain the absorption spectra of pure α-lactose monohydrate samples, twelve mixtures of α-lactose monohydrate standard and polyethylene were prepared at concentrations of 0%, 1%, 3%, 5%, 10%, 15%, 20%, 49%, 80% and 100% (*w*/*w*). The 0% and 100% (*w*/*w*) samples were prepared in duplicates. Mixtures were grounded to fine powders and mixed in a mortar until well-combined. They were formed into disc pellets of 13 mm diameter and approximately 100 mg weight. Each mixture was pressed into pellets with a hydraulic press under a pressure of 7 tons for 5 min. The thicknesses of the pellets were between 0.550 and 0.980 mm. The mass and thickness of each pellet are reported in Table S1, Supplementary Materials. Pellets removed from the mold were stored in a plastic (PE) bag inside a dry cabinet.

Samples of commercial infant formula were used as a test set to assess the quantification models derived from the spectra of pure samples. The samples were prepared to a final weight of 100 mg (without PE), in a similar manner to the pure α-lactose monohydrate standards.

Spectroscopic measurements were conducted with a commercial THz-TDS (TF4-1511, Toptica Photonics, Germany) with a resolution of 0.005 THz at room temperature. The system is housed in a custom-made cabinet (Figure S1, Supplementary Materials). Radiated THz waves were delivered by four parabolic mirrors, propagated through the samples and then to the THz waves receiver. The signal transmitted from the sample holder (without a sample) was used as the reference signal $E_{\mathrm{ref}}\left(t\right)$ and the signal transmitted through the sample is subsequently obtained as the sample signal $E_{\mathrm{sam}}\left(t\right)$. The time domain pulse is transformed to the frequency domain with fast Fourier transform (FFT) based on $E_{\mathrm{ref}}\left(t\right)$ and $E_{\mathrm{sam}}\left(t\right)$, Equation (1):

(1) $$\tilde{E}\left(\omega \right)=E\left(\omega \right)e^{-i\varphi \left(\omega \right)}=\int E\left(t\right)e^{-i\omega t}\varphi \left(\omega \right)dt$$

where $E\left(\omega \right)$ and $\varphi \left(\omega \right)$ are the amplitude and phase of the electric field, respectively, and $E\left(t\right)$ is the time–domain waveform. The refractive index and absorption coefficient spectra were obtained using the optical constant extraction model by Dorney [31] and Duvillaret [32,33]. The refractive index $n\left(\omega \right)$ and absorption coefficient $\alpha \left(\omega \right)$ of the samples were calculated by the following Equations (2) and (3):

(2) $$n\left(\omega \right)=1+\frac{\varphi \left(\omega \right)c}{\omega d}$$

(3) $$\alpha \left(\omega \right)=\frac{2}{d}\ln\frac{4n\left(\omega \right)}{\rho \left(\omega \right){\left[n\left(\omega \right)+1\right]}^{2}}$$

where *d* is the thickness of the sample, *c* = 3 × 10^8^ ms^−1^ is the speed of electromagnetic wave in vacuum, $\varphi \left(\omega \right)$ is the phase difference between the sample signal and the reference signal, and $\rho \left(\omega \right)$ is the module ratio of the amplitude of sample signal $E{\left(\omega \right)}_{\mathrm{sam}}$ to the amplitude of reference signal $E{\left(\omega \right)}_{\mathrm{ref}}$ after Fourier transform.

All spectra in this study are averaged results of over 100 acquisitions to optimise signal-to-noise ratio. Sets of THz-TDS measurements were performed under ambient conditions (≈37% RH) and in nitrogen-purged atmosphere (≤7% RH). The first set and second set were performed at sampling rates of 250 and 500 times per measurement, respectively, with replicate measurements in nitrogen atmosphere. Results presented in this paper are from the second dataset. Results from all datasets can be found in the Supplementary Materials.

Fourier transform of raw time–domain data, curve-fitting (Gaussian and Lorentzian) and baseline correction for the computation of peak area and height were performed with Python 3.10 programming language and Jupyter IDE (version 2022). Visualisation of spectra and linear regression analysis for the quantification models were performed using Wolfram Mathematica 12.3. Raw data and source codes (Jupyter and Mathematica notebooks) for analysis are available in the Supplementary Materials.

## 4. Conclusions

In this pilot study, a lactose detection and quantification procedure by THz-TDS was presented and evaluated with infant formula samples. Quantification models from the peak area and peak height of the absorption and absorption coefficient spectra were compared. The peak areas of two absorption peaks at frequencies of 0.53 THz and 1.37 THz resulted in a high quantification performance in pure α-lactose monohydrate standard samples. Although satisfactory results were obtained in pure standards, the prediction in infant formula samples may not be reflective of the total lactose content in the product. These were due to baseline shifts arising from the infant formula matrix and potential mutarotation of lactose anomers. Optimum conditions and predictors are also necessary for accurate quantification. Since THz-TDS is sensitive to different forms of the analyte, it may not be suitable for the accurate in-line determination of sugars in processed food and pharmaceutical products. THz-TDS can be a promising technique for the rapid, non-destructive and in-line detection of compounds. Nevertheless, caution must be taken in the quantitative analysis when the analyte can be present in a myriad of forms.

## Figures and Tables

**Table 1:** Comparison of common analytical methods for lactose quantification.

Method	Principle	Sample Preparation	Reagents
chromatographic: HPLC, HILIC, HPTLC, HPAEC	separation of compounds based on polarity, electrical charge or molecular size	sample purification, solid removal	solvents
spectrophotometric	conversion of lactose into its monomers for further reactions, followed by spectrophotometric quantification	hydrolysis of lactose, addition of reagents	solvent, enzyme, oxidising agents
spectroscopic: NMR	determination of molecular structure through chemical shifts when magnetic field is applied	sample purification, solid removal	solvents
spectroscopic: NIR, THz	determination of molecular structure through molecular vibrations	grounding, water removal	-

**Table 2:** Equation of trendline and RMSE of different prediction models for lactose quantification.

Predictor		Absorption Peak	Equation of Trendline	RMSE
Absorbance	Peak area	0.53 THz	3.43 × 10^−4^ *x* + 0.0021	0.0011
		1.37 THz	7.06 × 10^−4^ *x* + 0.0041	0.0047
	Peak height	0.53 THz	6.54 × 10^−3^ *x* + 0.0840	0.0664
		1.37 THz	6.46 × 10^−3^ *x* + 0.0990	0.0870
Absorption coefficient	Peak area	0.53 THz	1.98 × 10^−2^ *x* − 0.0288	0.0840
		1.37 THz	6.71 × 10^−2^ *x* − 0.2468	0.2127
	Peak height	0.53 THz	6.08 × 10^−1^ *x* − 0.6794	1.8840
		1.37 THz	1.06 *x* − 2.6018	3.1344

**Table 3:** Mean predicted concentration of lactose anhydrous in infant formula samples using 0.53 THz and 1.37 THz absorption peak area from THz-TDS measurements conducted in nitrogen atmosphere, mean concentration obtained from HPLC measurements in external laboratories and product nutritional labelling.

		Mean Concentration ± SD in % (*w*/*w*)		
		Sample 1	Sample 2	Sample 3
Absorbance	0.53 THz	39.0 ± 1.6	1.8 ± 1.0	–19.4 ± 1.5
	1.37 THz	45.1 ± 1.5	11.4 ± 0.7	0.0 ± 0.6
Absorption coefficient	0.53 THz	62.5 ± 4.4	23.6 ± 9.2	1.8 ± 10.1
	1.37 THz	68.4 ± 1.6	20.8 ± 0.5	not detected *
HPLC (from external laboratories)		61.97 (*n* = 2)	59.97 (*n* = 3)	not done
Product label: carbohydrate content		60	58	assumed to be 0 (lactose-free)

*Absorption coefficient <–0.1

## Data Availability

All data are included in the Supplementary Materials.

## Supplementary Materials

The following supporting information can be downloaded at: https://www.mdpi.com/article/10.3390/molecules27155040/s1, Table S1: Pellet mass and thickness of pure α-lactose monohydrate standards and infant formula samples for THz-TDS measurement; Figure S1: Custom-made THz-TDS system in this study at National Electronics and Computer Technology Centre, Pathum Thani, Thailand (a zoom-in of the sample holder is framed in red); Figure S2: Product label of infant formula Sample 1; Figure S3: Product label of infant formula Sample 2; Figure S4: Product label of infant formula Sample 3; data and source codes.

## Sample Availability Statement

Samples of the compounds are not available from the authors. Sample information and their sources are reported in the Supplementary Materials.
