# Supplementary material for: Terahertz Spectroscopic Analysis of Lactose in Infant Formula: Implications for Detection and Quantification
Source: Molecules. 2022 Aug 8;27(15):5040. doi: 10.3390/molecules27155040 (PMC9370465; doi:10.3390/molecules27155040)
Supplement: Supplementary file 1 [file molecules-27-05040-s001.zip › codes/data-processing.pdf]

```
# DOI: 10.3390/molecules27155040
```

Module import

```
%matplotlib inline
import matplotlib.pyplot as plt
import matplotlib as mpl
import glob
import numpy as np
import pandas as pd
import rampy as rp
import scipy.interpolate as spi
from scipy.optimize import curve_fit

mpl.rcParams['figure.figsize'] = [6, 4]
```

Transmission and absorption spectra

```
dataset = 'set2_N2_5' # choose dataset folder here
path = 'C:/Lactose/' + dataset + '/time_domain/'
plt.figure(figsize=(12, 6))

for filename in glob.glob(path + '*.csv'):

    # import raw time-domain data from csv files
    signal_td = pd.read_csv(filename, sep = ',', encoding ='utf-8' , names = ["Time

    # reformat time, reference signal and sample signal data according to NumPy
    count_nonfloat = 0
    time = []
    ref = []
    sam = []

    for i in range(len(signal_td.Time)):
        try:
            time.append(float(signal_td.Time[i]))
            ref.append(float(signal_td.Ref[i]))
            sam.append(float(signal_td.Sam[i]))
        except ValueError:
            if count_nonfloat == 1:
                break
            else:
                count_nonfloat += 1

    # apply Fourier Transform to reference signal and sample signal
    # frequency
    freq = np.fft.rfftfreq(len(time), 0.05)
```

```
    # amplitude
    ref_fd = abs(np.fft.rfft(ref))
    sam_fd = abs(np.fft.rfft(sam))

    # normalise spectra to evaluate transmittance and absorbance
    beta = 0.0001
    sig_trans_fd = np.sqrt(sam_fd**2 + beta**2)/np.sqrt(ref_fd**2 + beta**2)
    sig_absorp_fd = 1 - sig_trans_fd

    # visualise spectra

    plt.plot(freq, sig_trans_fd, label = '{}: Fd TR'.format(filename[len(path):]))

    df = pd.DataFrame({'Frequency (THz)' : freq, 'Transmittance': sig_trans_fd, 'Ab
    df.to_csv(path + "../frequency_domain/" + filename[len(path):], index = False,


plt.xlabel('Frequency (THz)')
plt.ylabel('Transmittance (arbitrary unit)')
plt.legend()
plt.xlim(0.1, 3.5)
plt.ylim(-0.5, 1.5)
plt.show()
```

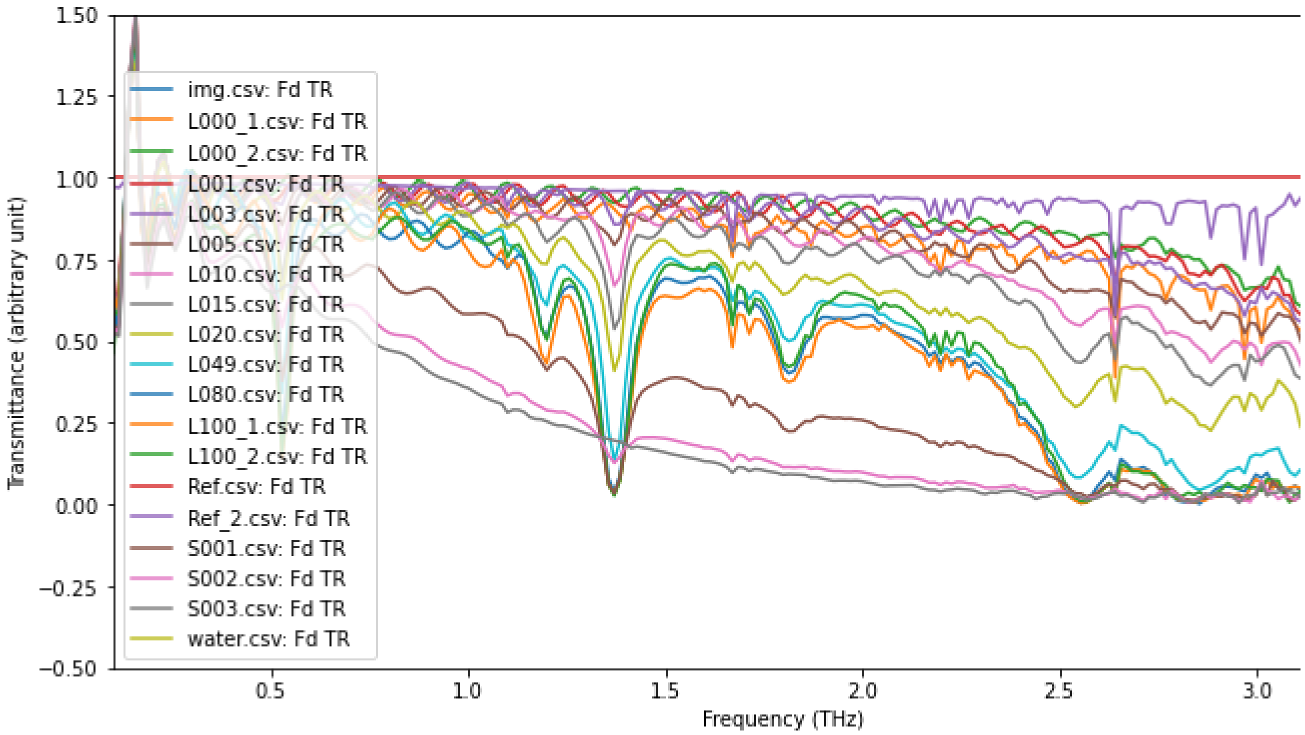

Peak area and peak height calculation

```
#Functions for Lorentzian and Gaussian fitting
def lorentz(x, I, gamma, x0):
    return I * gamma**2 / ((x - x0)**2 + gamma**2)
```

```
def gaussian(x, I, mu, sig):
    [illegible]

dataset = 'set2_N2_5' #choose dataset folder here
path = 'C:/Lactose/' + dataset + '/time_domain/'
filelist = ['L000_1','L000_2','L001','L003','L005','L010','L015','L020','L049','L08
for i in range(len(filelist)):
    filelist[i] = path + filelist[i] + '.csv'

peakvalues = []
for filename in filelist:

    # import raw time-domain data from csv files
    signal_td = pd.read_csv(filename, sep = ',', encoding ='utf-8' , names = ["Time

    # reformat time, reference signal and sample signal data according to NumPy
    count_nonfloat = 0
    time = []
    ref = []
    sam = []

    for i in range(len(signal_td.Time)):
        try:
            time.append(float(signal_td.Time[i]))
            ref.append(float(signal_td.Ref[i]))
            sam.append(float(signal_td.Sam[i]))
        except ValueError:
            if count_nonfloat == 1:
                break
            else:
                count_nonfloat += 1

    # apply Fourier Transform to reference signal and sample signal
    # frequency
    freq = np.fft.rfftfreq(len(time), 0.05)

    # amplitude
    ref_fd = abs(np.fft.rfft(ref))
    sam_fd = abs(np.fft.rfft(sam))

    # normalise spectra to evaluate transmittance and absorbance
    beta = 0.0001
    sig_trans_fd = np.sqrt(sam_fd**2 + beta**2)/np.sqrt(ref_fd**2 + beta**2)
    sig_absorp_fd = 1 - sig_trans_fd

    begin_f = 0.3
    end_f = 3

    mask = (freq >= begin_f)*(freq <= end_f)
    freq = freq[mask]
    sig_absorp_fd = sig_absorp_fd[mask]

    enable_interpolation = False
    if enable_interpolation:
        N = 2000
        f = spi.interp1d(freq, sig_absorp_fd)
```

```
        freq = np.linspace(freq[0], freq[-1], N)
        sig_absorp_fd = f(freq)
        sig_absorp_fd = np.array(sig_absorp_fd)
    sig_absorp_fd = np.reshape(sig_absorp_fd, (len(sig_absorp_fd), 1))

    check = []
    for i in sig_absorp_fd:
        if i not in check:
            check.append(i[0])
    list=[]

    if check != [0.0]:
        corrected_poly, baseline_poly = rp.baseline(freq, sig_absorp_fd, np.array([
        corrected_als, baseline_als = rp.baseline(freq, sig_absorp_fd, np.array([[f
        corrected_arpls, baseline_arpls = rp.baseline(freq, sig_absorp_fd, np.array
        corrected_drpls, baseline_drpls = rp.baseline(freq, sig_absorp_fd, np.array

        for (begin_roi, end_roi) in [(0.46, 0.60), (1.27, 1.47)]: # center frequenc
            if False:
                plt.axvline(x = begin_roi, color = 'red', linestyle = '--')
                plt.axvline(x = end_roi, color = 'red', linestyle = '--')
                plt.plot(freq, sig_absorp_fd, label = 'Normalized absorption')
                plt.plot(freq, baseline_poly, label = 'Baseline-poly')
                plt.plot(freq, baseline_als, label = 'Baseline-als')
                plt.plot(freq, baseline_arpls, label = 'Baseline-arpls')
                plt.plot(freq, baseline_drpls, label = 'Baseline-drpls')
                plt.xlabel('Frequency (THz)')
                plt.ylabel('Amplitude (N/A)')
                plt.title('{}: Frequency domain'.format(filename[len(path) + 1:]))
                plt.legend()
                plt.xlim(begin_f, end_f)
                plt.ylim(-0.2, 1.2)
                plt.show()

            # remove baseline using drPLS method
            mask = (freq >= begin_roi)*(freq <= end_roi)
            corrected_absorp_fd = np.ravel(sig_absorp_fd - baseline_drpls)

            popt_l, pcov_l = curve_fit(lorentz, freq[mask], corrected_absorp_fd[mas
                                       p0 = [0.1, (begin_roi + end_roi)/2, 5*(end_roi
            popt_g, pcov_g = curve_fit(gaussian, freq[mask], corrected_absorp_fd[ma
                                       p0 = [0.1, (begin_roi + end_roi)/2, (end_roi -

            lorentz_absorp_fd = lorentz(freq[mask], popt_l[0], popt_l[1], popt_l[2]
            guassian_absorp_fd = gaussian(freq[mask], popt_g[0], popt_g[1], popt_g[
            lorentz_height_fd = lorentz(popt_l[2], popt_l[0], popt_l[1], popt_l[2])
            gaussian_height_fd = gaussian(popt_g[1], popt_g[0], popt_g[1], popt_g[2

            area_numer = np.trapz(corrected_absorp_fd[mask], freq[mask])
            area_lorentz = np.trapz(lorentz_absorp_fd, freq[mask])
            area_gaussian = np.trapz(guassian_absorp_fd, freq[mask])

            list.append(area_gaussian)
            list.append(gaussian_height_fd)
```

```
        if True:
            plt.axvline(x = np.min(freq[mask]), color = 'black', linestyle = '-
            plt.axvline(x = np.max(freq[mask]), color = 'black', linestyle = '-
            plt.scatter(freq, corrected_absorp_fd, color = 'blue', label = 'Cor
            plt.plot(freq[mask], lorentz_absorp_fd, color = 'orange', label = '
            plt.plot(freq[mask], guassian_absorp_fd, color = 'green', label = '
            plt.xlabel('Frequency (THz)')
            plt.ylabel('Amplitude (N/A)')
            plt.title('{}: range = [{:.2f}, {:.2f}] THz'.format(filename[len(pa
                    '\nArea (numerical, lorentz, gaussian) = ({:.3f}, {:.3f}, {
                    '\nHeight (lorentz, gaussian) = ({:.3f}, {:.3f})'.format(lo
            plt.xlim(begin_roi - 0.05, end_roi + 0.05)
            plt.ylim(-0.2, 1.2)
            plt.legend()
            plt.show()
    else:
        list = ['de', 'de', 'de', 'de']

    peakvalues.append([filename[len(path)],int(filename[len(path) + 1:len(path) + 4
```

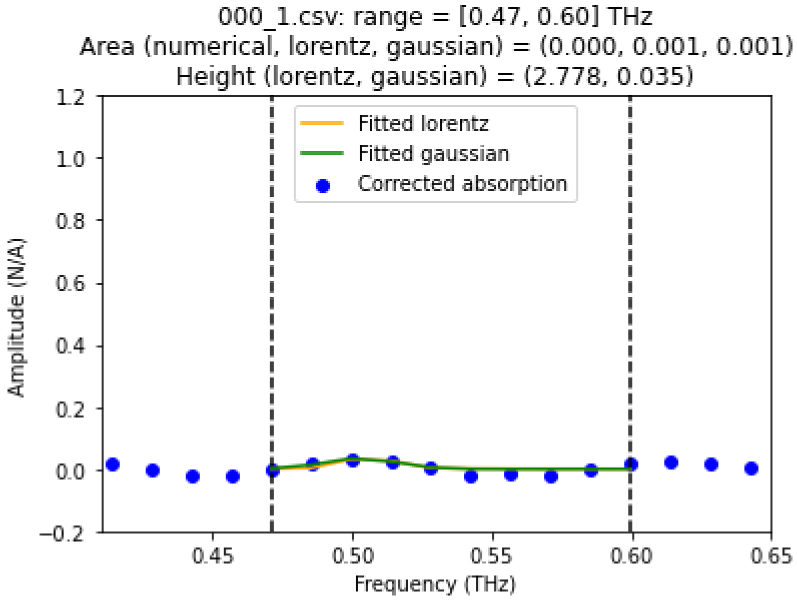

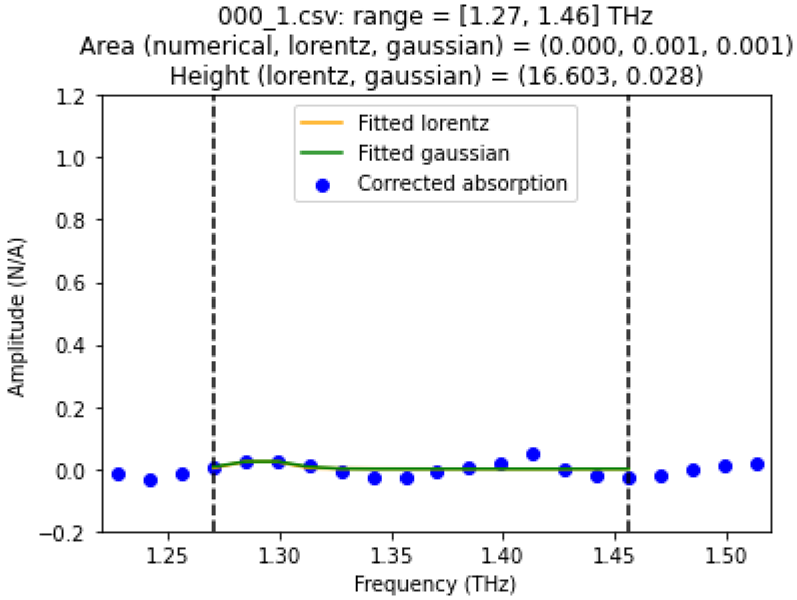

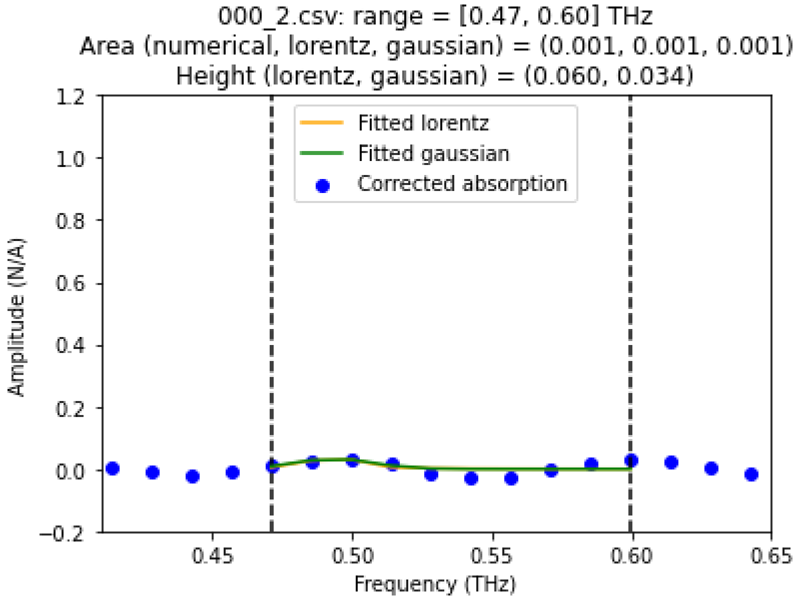


```
C:\Users\ASUS\anaconda3\lib\site-packages\scipy\optimize\minpack.py:833: Optim
  warnings.warn('Covariance of the parameters could not be estimated',
```

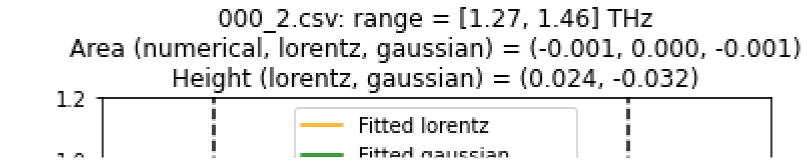

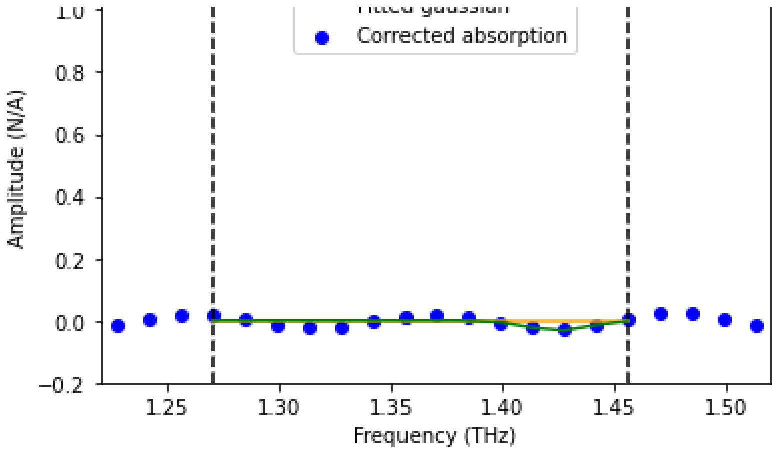

001.csv: range = [0.47, 0.60] THz
Area (numerical, lorentz, gaussian) = (0.001, 0.001, 0.001)
Height (lorentz, gaussian) = (0.054, 0.039)

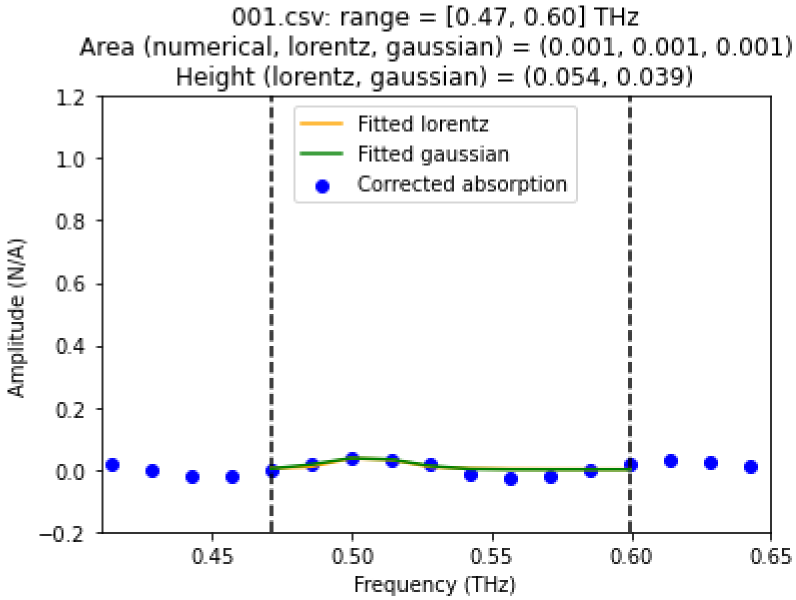

001.csv: range = [1.27, 1.46] THz
Area (numerical, lorentz, gaussian) = (0.001, -0.000, 0.001)
Height (lorentz, gaussian) = (-0.003, 0.028)

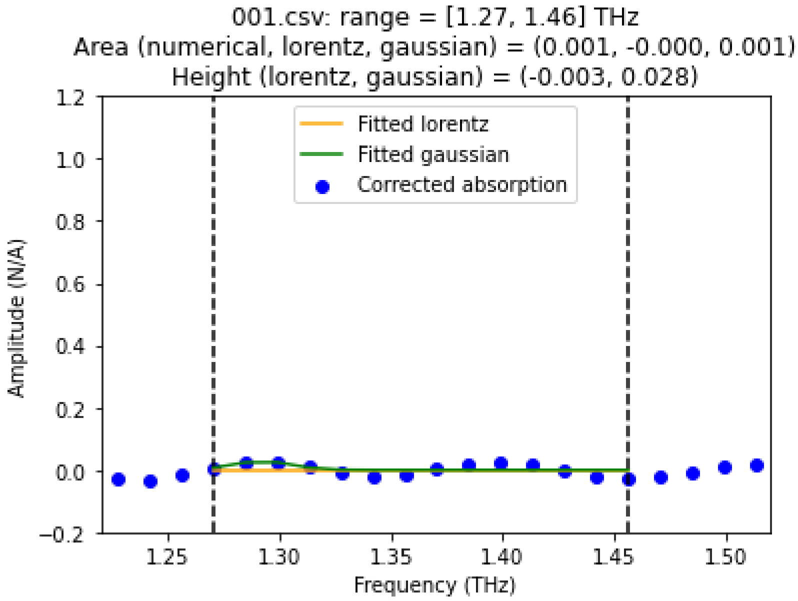

003.csv: range = [0.47, 0.60] THz
Area (numerical, lorentz, gaussian) = (0.002, 0.003, 0.002)
Height (lorentz, gaussian) = (0.049, 0.050)

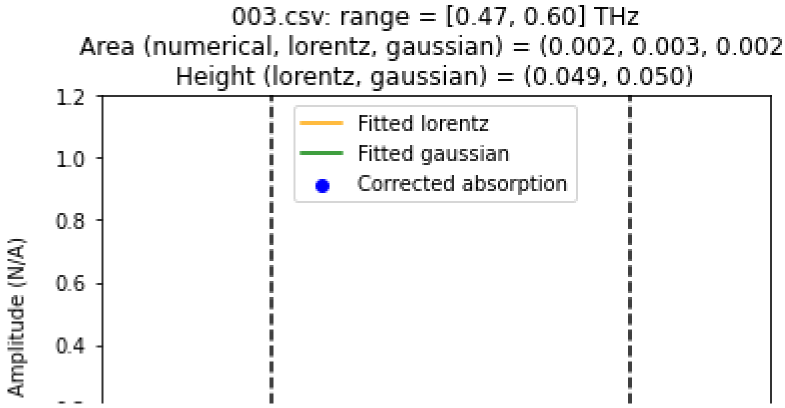

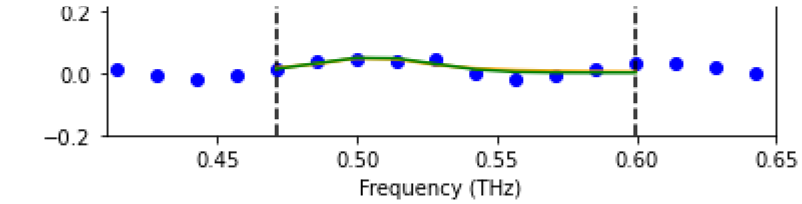

003.csv: range = [1.27, 1.46] THz
Area (numerical, lorentz, gaussian) = (0.002, 0.003, 0.003)
Height (lorentz, gaussian) = (0.108, 0.081)

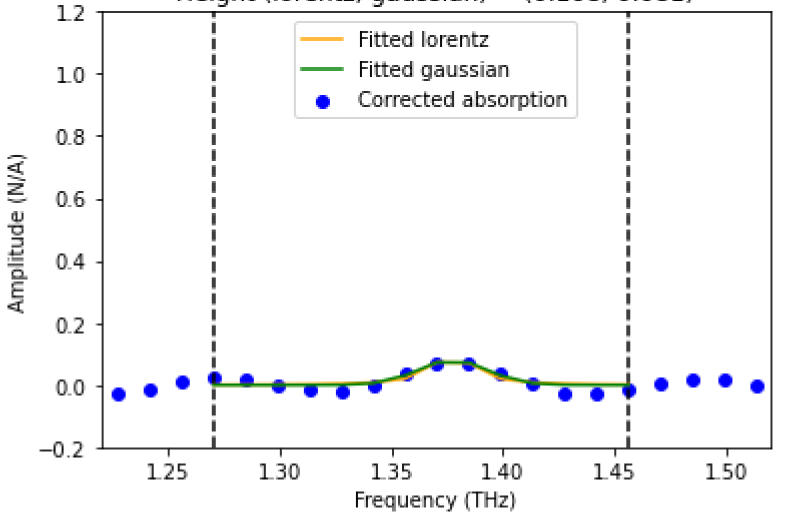

005.csv: range = [0.47, 0.60] THz
Area (numerical, lorentz, gaussian) = (0.003, 0.004, 0.004)
Height (lorentz, gaussian) = (0.112, 0.099)

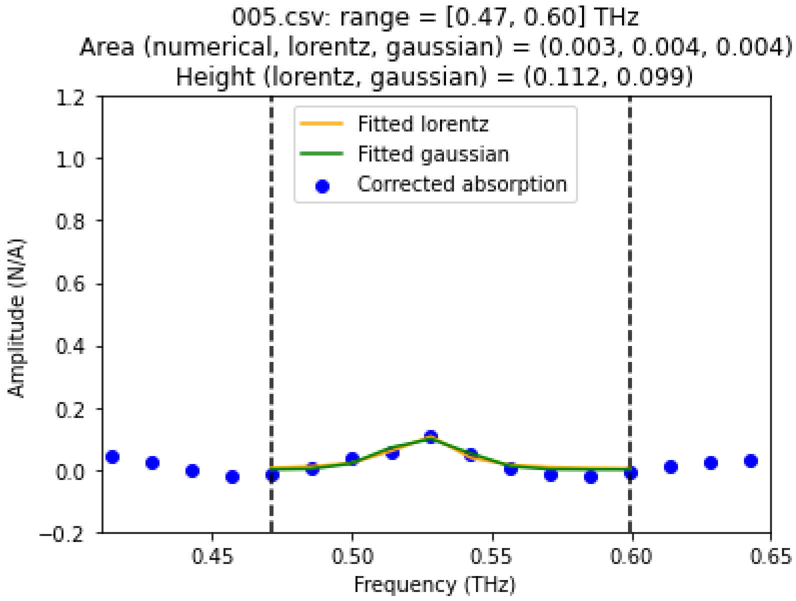

005.csv: range = [1.27, 1.46] THz
Area (numerical, lorentz, gaussian) = (0.007, 0.007, 0.007)
Height (lorentz, gaussian) = (0.110, 0.099)

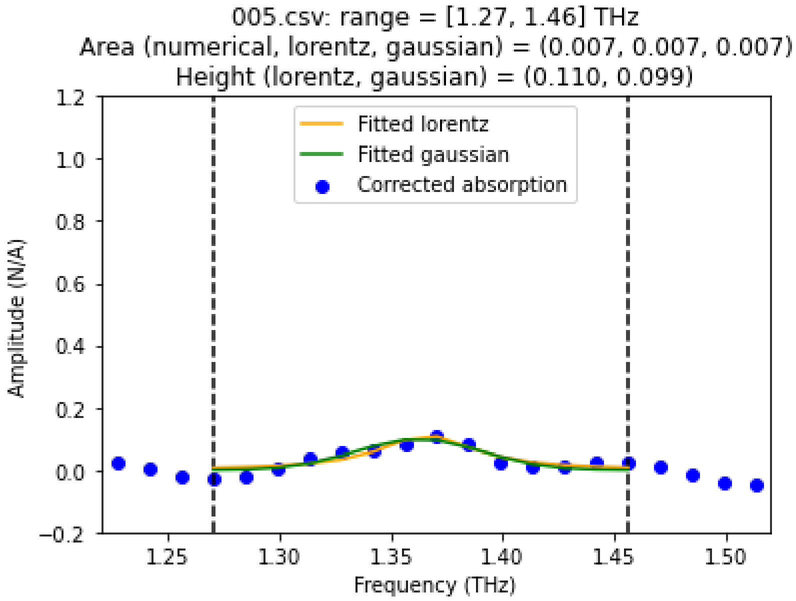

010.csv: range = [0.47, 0.60] THz
[illegible]

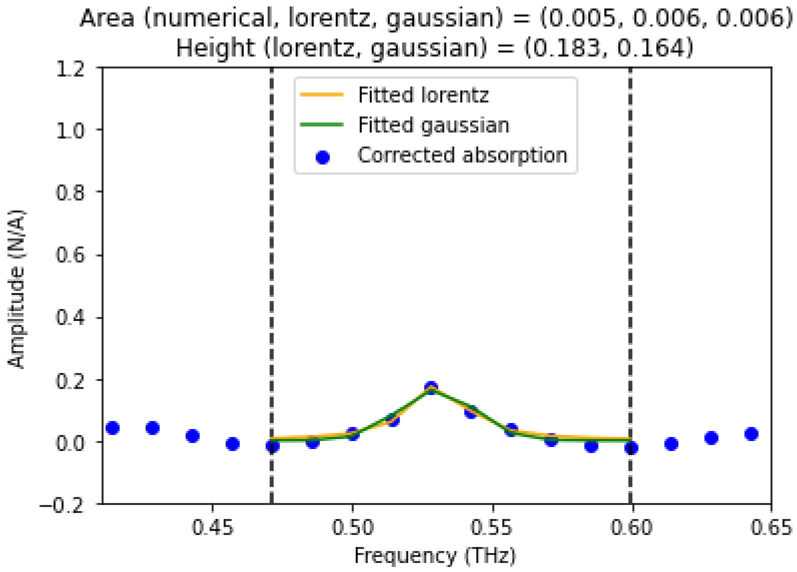
Area (numerical, lorentz, gaussian) = (0.005, 0.006, 0.006)
Height (lorentz, gaussian) = (0.183, 0.164)
Fitted lorentz
Fitted gaussian
Corrected absorption
Amplitude (N/A)
Frequency (THz)

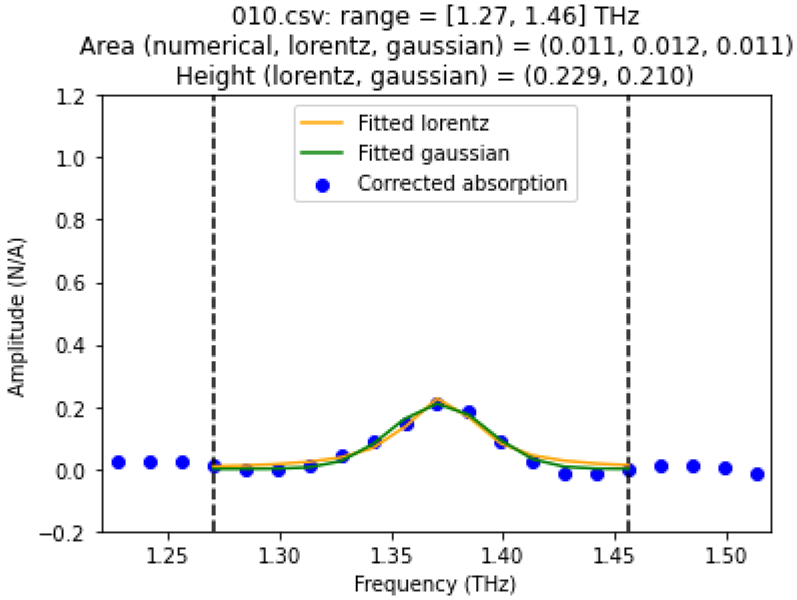
010.csv: range = [1.27, 1.46] THz
Area (numerical, lorentz, gaussian) = (0.011, 0.012, 0.011)
Height (lorentz, gaussian) = (0.229, 0.210)
Fitted lorentz
Fitted gaussian
Corrected absorption
Amplitude (N/A)
Frequency (THz)

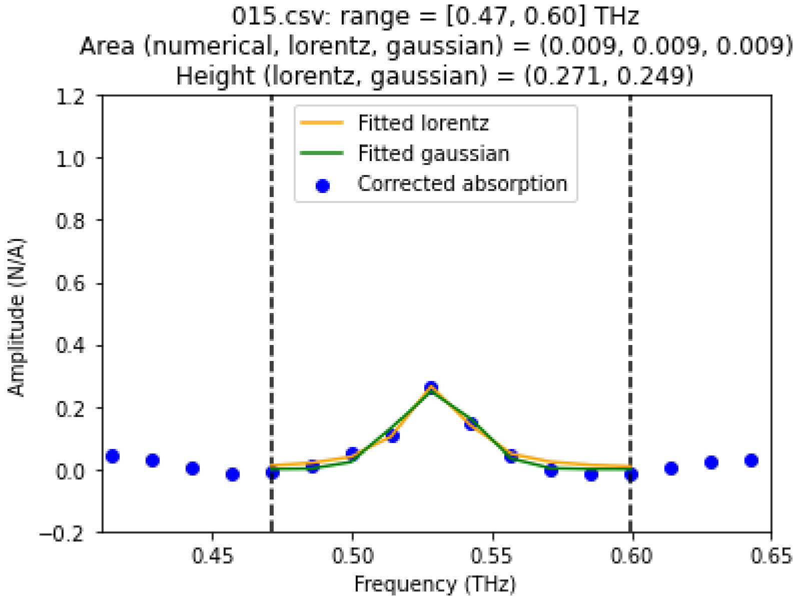
015.csv: range = [0.47, 0.60] THz
Area (numerical, lorentz, gaussian) = (0.009, 0.009, 0.009)
Height (lorentz, gaussian) = (0.271, 0.249)
Fitted lorentz
Fitted gaussian
Corrected absorption
Amplitude (N/A)
Frequency (THz)

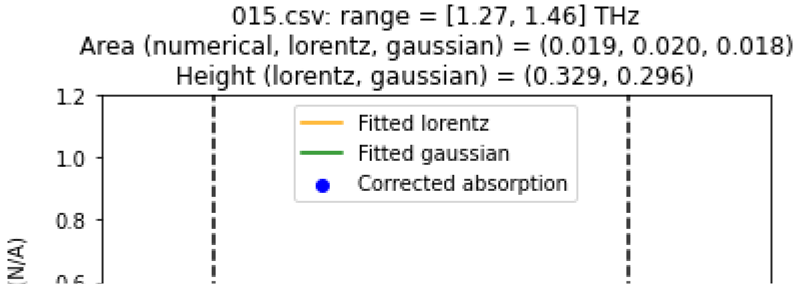
015.csv: range = [1.27, 1.46] THz
Area (numerical, lorentz, gaussian) = (0.019, 0.020, 0.018)
Height (lorentz, gaussian) = (0.329, 0.296)
Fitted lorentz
Fitted gaussian
Corrected absorption

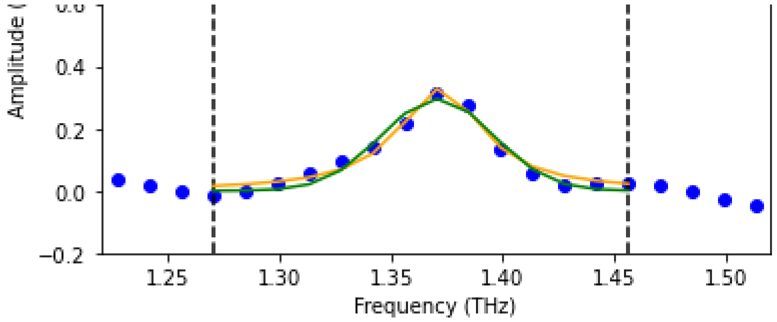

020.csv: range = [0.47, 0.60] THz
Area (numerical, lorentz, gaussian) = (0.013, 0.012, 0.011)
Height (lorentz, gaussian) = (0.338, 0.301)

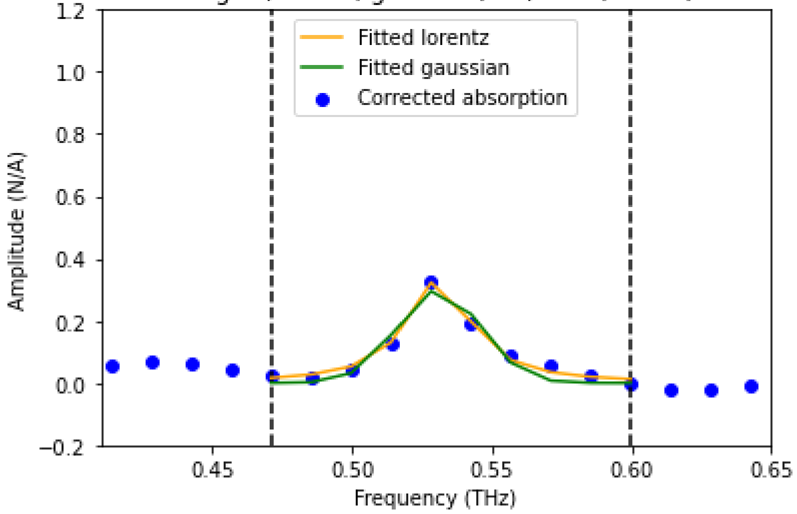

020.csv: range = [1.27, 1.46] THz
Area (numerical, lorentz, gaussian) = (0.028, 0.028, 0.027)
Height (lorentz, gaussian) = (0.394, 0.351)

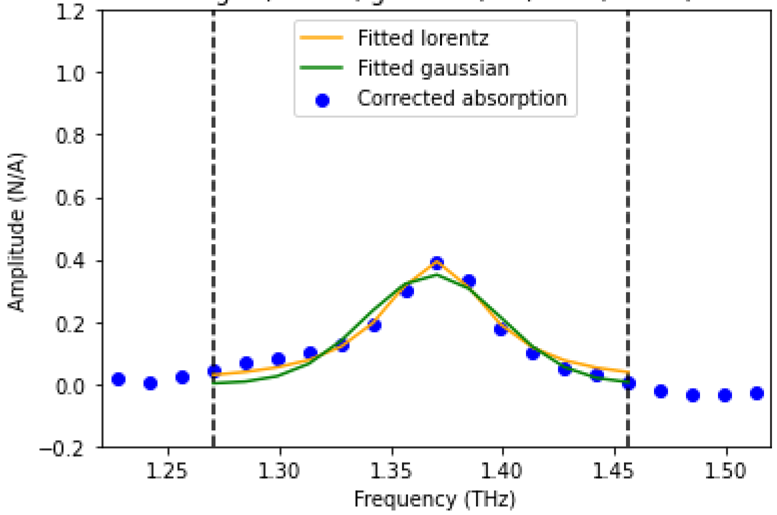

049.csv: range = [0.47, 0.60] THz
Area (numerical, lorentz, gaussian) = (0.024, 0.023, 0.020)
Height (lorentz, gaussian) = (0.604, 0.555)

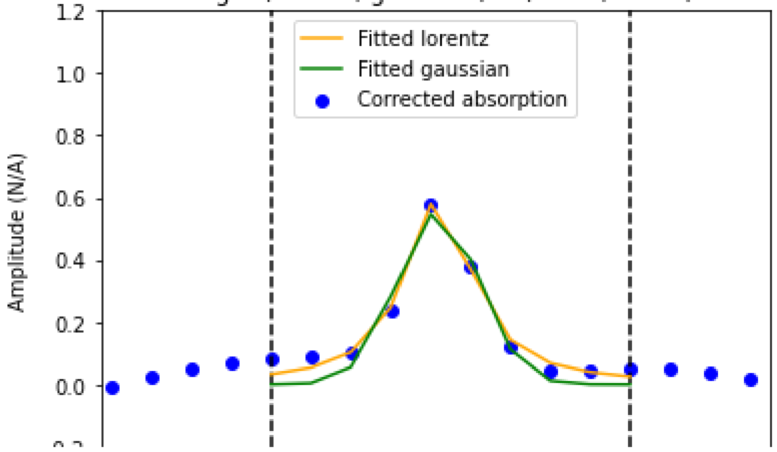

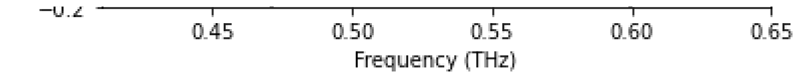

049.csv: range = [1.27, 1.46] THz
Area (numerical, lorentz, gaussian) = (0.049, 0.050, 0.048)
Height (lorentz, gaussian) = (0.631, 0.576)

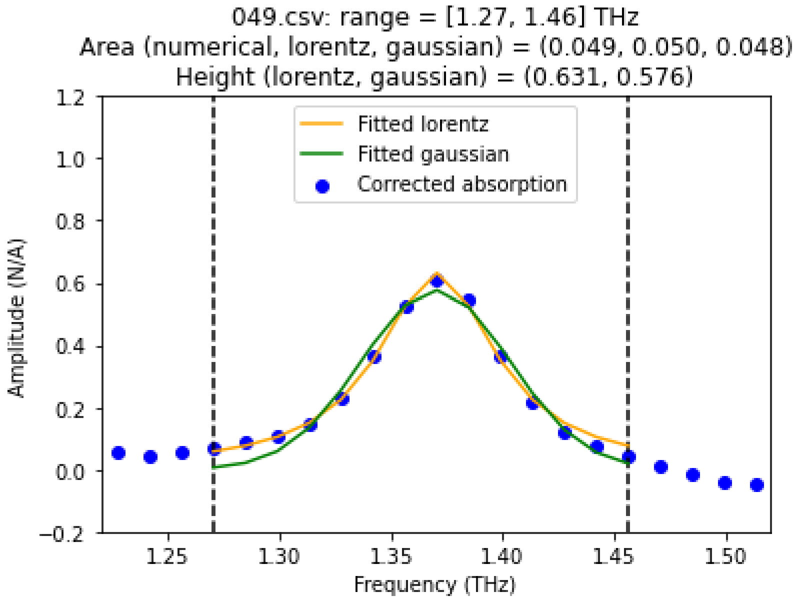

080.csv: range = [0.47, 0.60] THz
Area (numerical, lorentz, gaussian) = (0.032, 0.032, 0.030)
Height (lorentz, gaussian) = (0.703, 0.624)

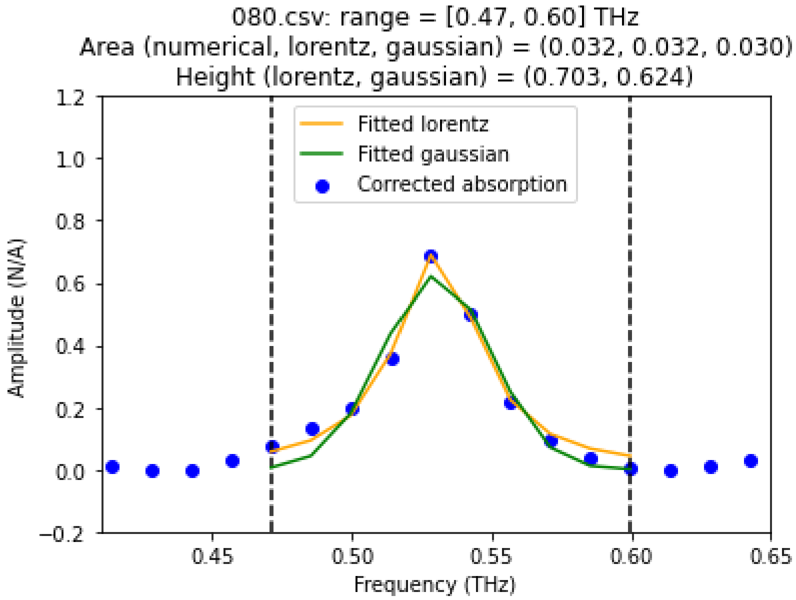

080.csv: range = [1.27, 1.46] THz
Area (numerical, lorentz, gaussian) = (0.064, 0.064, 0.063)
Height (lorentz, gaussian) = (0.702, 0.652)

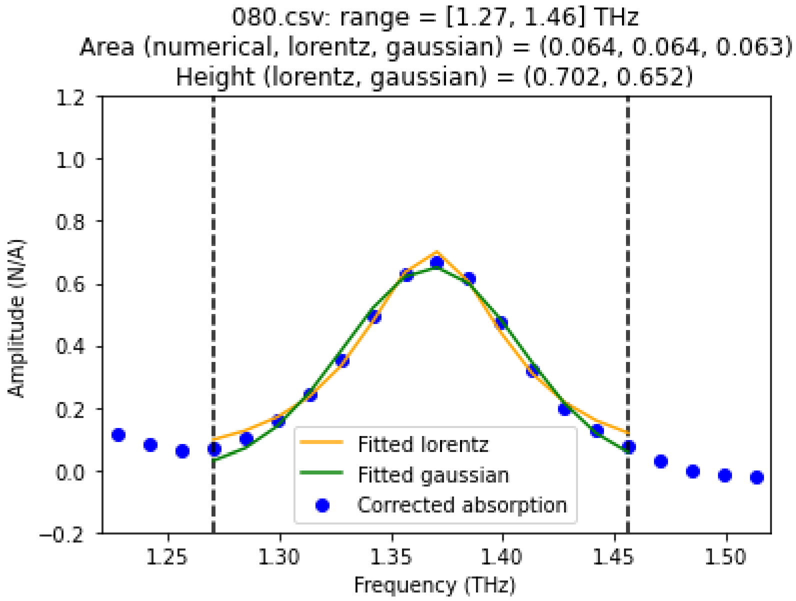

100_1.csv: range = [0.47, 0.60] THz
Area (numerical, lorentz, gaussian) = (0.037, 0.037, 0.035)
Height (lorentz, gaussian) = (0.781, 0.701)

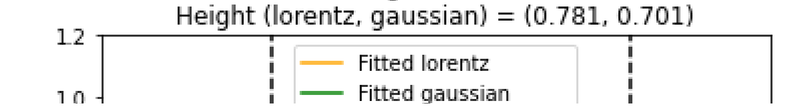

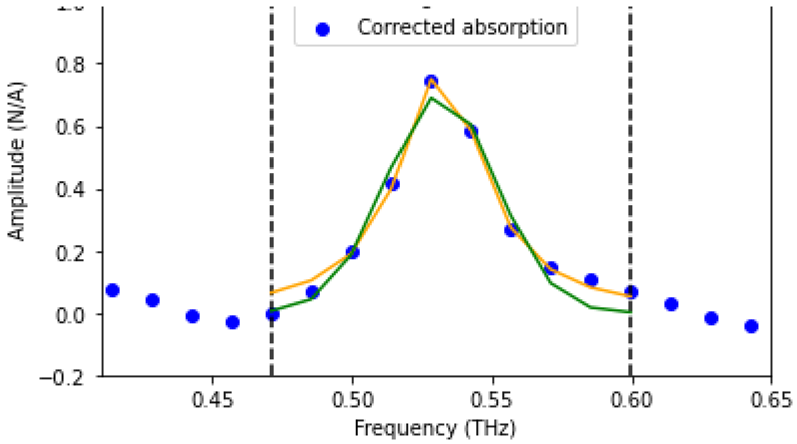

100_1.csv: range = [1.27, 1.46] THz
Area (numerical, lorentz, gaussian) = (0.068, 0.069, 0.068)
Height (lorentz, gaussian) = (0.684, 0.638)

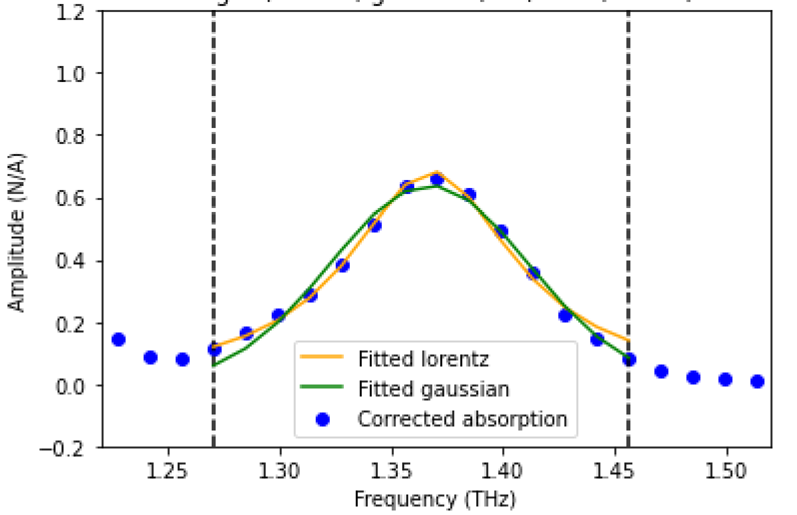

100_2.csv: range = [0.47, 0.60] THz
Area (numerical, lorentz, gaussian) = (0.039, 0.038, 0.036)
Height (lorentz, gaussian) = (0.744, 0.663)

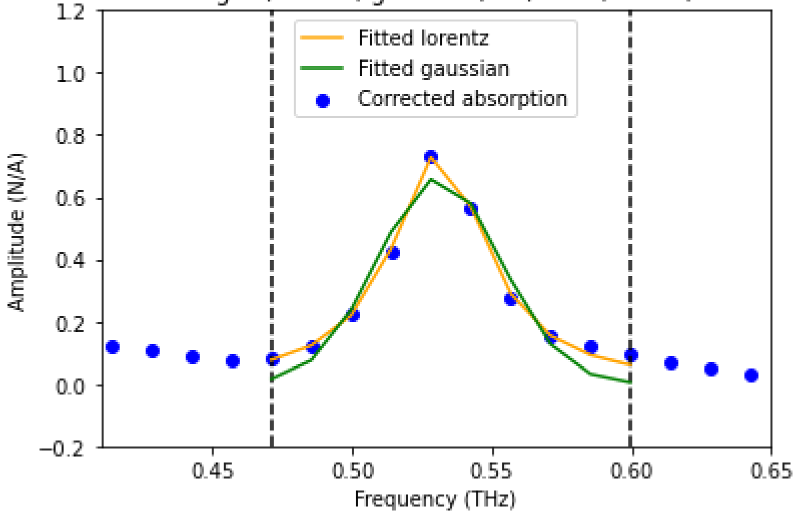

100_2.csv: range = [1.27, 1.46] THz
Area (numerical, lorentz, gaussian) = (0.073, 0.074, 0.073)
Height (lorentz, gaussian) = (0.754, 0.704)

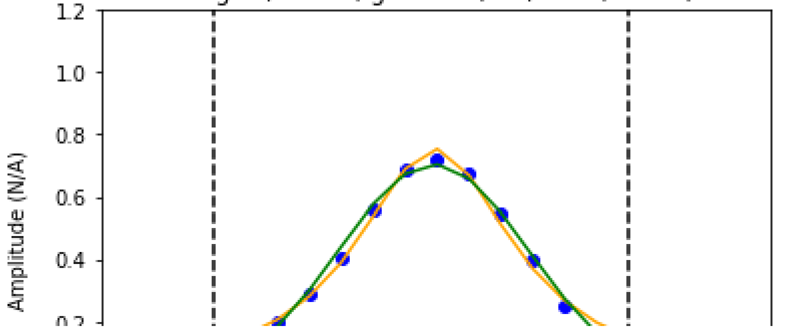

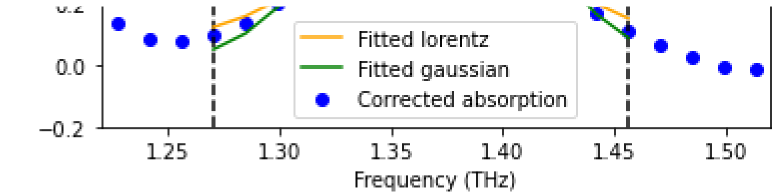

001.csv: range = [0.47, 0.60] THz
Area (numerical, lorentz, gaussian) = (0.018, 0.018, 0.017)
Height (lorentz, gaussian) = (0.448, 0.393)

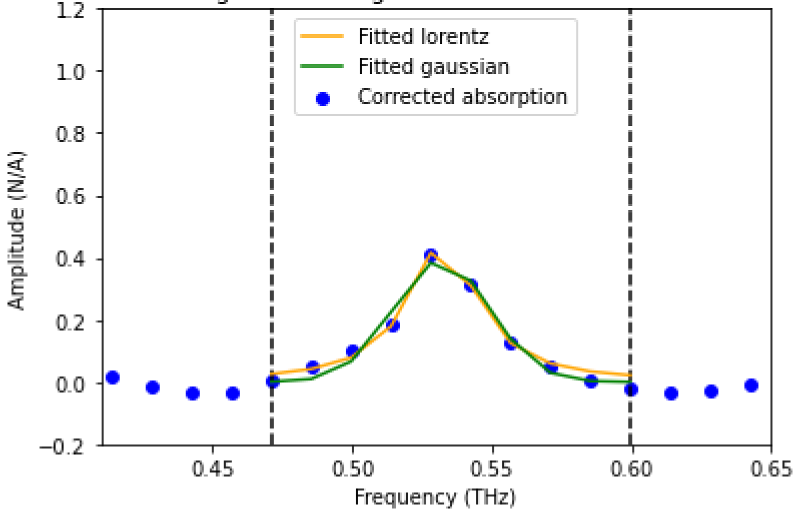

001.csv: range = [1.27, 1.46] THz
Area (numerical, lorentz, gaussian) = (0.040, 0.040, 0.039)
Height (lorentz, gaussian) = (0.428, 0.390)

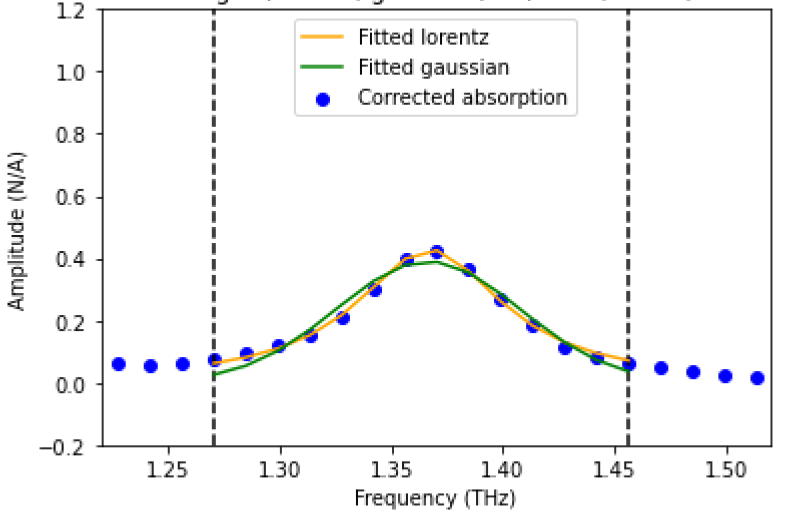

002.csv: range = [0.47, 0.60] THz
Area (numerical, lorentz, gaussian) = (0.001, 0.003, 0.003)
Height (lorentz, gaussian) = (0.186, 0.127)

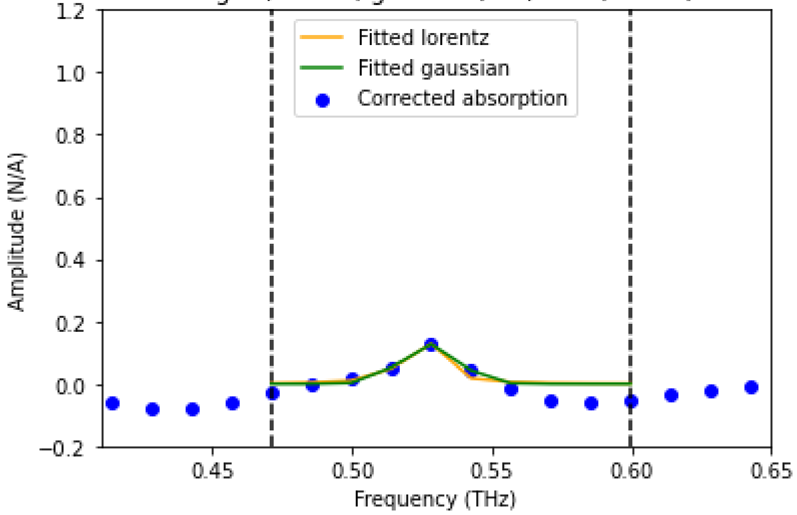

002.csv: range = [1.27, 1.46] THz
[illegible]

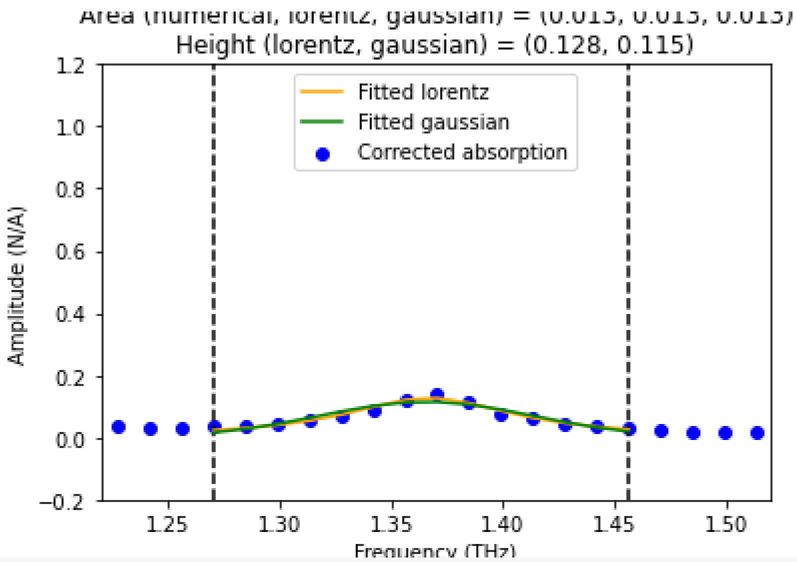


```
#export peak area and height to csv file
df = pd.DataFrame(data = peakvalues, columns = ['Sample', '', 'Area-0.53', 'Area-1.
print(df)
df.to_csv(path + "../peak_area_and_height/absorption_spectra/peakvalues-drpls.csv",
```

	Sample		Area-0.53	Area-1.37	Height-0.53	Height-1.37
0	L	0	0.001113	0.000822	0.035172	0.027639
1	L	0	0.001083	-0.001075	0.033645	-0.032486
2	L	1	0.001415	0.000873	0.039060	0.028448
3	L	3	0.002426	0.003115	0.049609	0.080700
4	L	5	0.003597	0.006893	0.099479	0.098587
5	L	10	0.005580	0.011222	0.163912	0.209963
6	L	15	0.008527	0.018464	0.249142	0.295719
7	L	20	0.011124	0.026568	0.300789	0.351372
8	L	49	0.020147	0.047547	0.555253	0.576063
9	L	80	0.030461	0.063180	0.624083	0.651658
10	L	100	0.034521	0.068109	0.700651	0.637692
11	L	100	0.036290	0.072721	0.662827	0.704488
12	S	1	0.016886	0.039177	0.393013	0.390088
13	S	2	0.003217	0.013022	0.126540	0.114893
14	S	3	-0.004368	0.004410	-0.059387	0.026211

Refractive index and absorption coefficient spectra

```
dataset = 'set2_N2_5' #choose dataset folder here
path = 'C:/Lactose/' + dataset + '/time_domain/'
filelist = ['L000_1','L000_2','L001','L003','L005','L010','L015','L020','L049','L08
```

```
import csv, math

Re = np.zeros([701,18])
Ab = np.zeros([701,18])
k = 1

#Sample Signal
for filename in filelist :

    signal = pd.read_csv(path + filename + '.csv', sep = ',', encoding ='utf-8' , n
```

```
# sample thickness
if filename == 'L001' :
    Sample_thickness = 0.934
elif filename == 'L003' :
    Sample_thickness = 0.982
elif filename == 'L005' :
    Sample_thickness = 0.908
elif filename == 'L010' :
    Sample_thickness = 0.866
elif filename == 'L015' :
    Sample_thickness = 0.885
elif filename == 'L020' :
    Sample_thickness = 0.828
elif filename == 'L049' :
    Sample_thickness = 0.712
elif filename == 'L080' :
    Sample_thickness = 0.625
elif filename == 'L100_1' or 'L100_2' :
    Sample_thickness = 0.553
elif filename == 'S001' :
    Sample_thickness = 0.587
elif filename == 'S002' :
    Sample_thickness = 0.582
elif filename == 'S003' :
    Sample_thickness = 0.585
else:
    Sample_thickness = 0.977

s = []
r = []
t = []
for i in range(len(signal.Time)):
    t.append(float(signal.Time[i]))
    s.append(float(signal.Sam[i]))
    r.append(float(signal.Ref[i]))

#Fourier Transform
freq = np.fft.rfftfreq(len(t), 0.05)
sam_fd = np.fft.rfft(s)
ref_fd = np.fft.rfft(r)

sp = []
rp = []
for i in range(len(freq)):
    # sample and reference phase
    sp.append(np.angle(sam_fd[i]/ref_fd[i]))
    rp.append(np.angle(ref_fd[i]/ref_fd[i]))
    Re[i,0] = freq[i]
    Ab[i,0] = freq[i]

#Phase unwrapping
threshold = 22/7
for i in range(1,len(sp)-1):
    if sp[i+1]-sp[i] > threshold :
```

```
        for j in range (i+1,len(sp)):
            sp[j] = sp[j] - 2*22/7
    elif sp[i+1]-sp[i] < -threshold :
        for j in range (i+1,len(sp)):
            sp[j] = sp[j] + 2*22/7

for i in range(1,len(rp)-1):
    if rp[i+1]-rp[i] > threshold :
        for j in range (i+1,len(rp)):
            rp[j] = rp[j] - 2*22/7
    elif rp[i+1]-rp[i] < -threshold :
        for j in range (i+1,len(rp)):
            rp[j] = rp[j] + 2*22/7

tran = []
absorb = []
Refractive = []
a = []
for i in range(len(freq)):
    # phase difference
    sp[i] = abs(sp[i] - rp[i])
    # transmittance
    tran.append(abs(sam_fd[i])/abs(ref_fd[i]))
    # absorbance
    absorb.append(1 - tran[i])
    # refractive index
    Refractive.append((((3*(10**8))*sp[i])/(2*(22/7)*((freq[i])*10**12)*((Sampl
    Re[i,k] = Refractive[i]
    # absorption coefficient
    a.append((2/((Sample_thickness)/10))*(math.log(((4*(Refractive[i]))/(((Refr
    Ab[i,k] = a[i]
k += 1

plt.figure(figsize = (12, 6))
plt.subplot(121)
plt.title('Refractive Index of ' + filename)
plt.plot(freq, Refractive, 'b')
plt.xlabel('Freq (THz)')
plt.ylabel('Refractive index')
plt.xlim(0.3,2)
plt.ylim(0.8,2)

plt.subplot(122)
plt.title('Absorption Coefficient of ' + filename)
plt.plot(freq, a, label = '' + filename)
plt.xlabel('Freq (THz)')
plt.ylabel('Absorption Coefficient cm$^{-1}$')
plt.xlim(0.3,2)
plt.show()
```

```
C:\Users\ASUS\AppData\Local\Temp/ipykernel_13884/1253449618.py:92: RuntimeWarn
  Refractive.append((((3*(10**8))*sp[i])/(2*(22/7)*((freq[i])*10**12)*((Sample
```

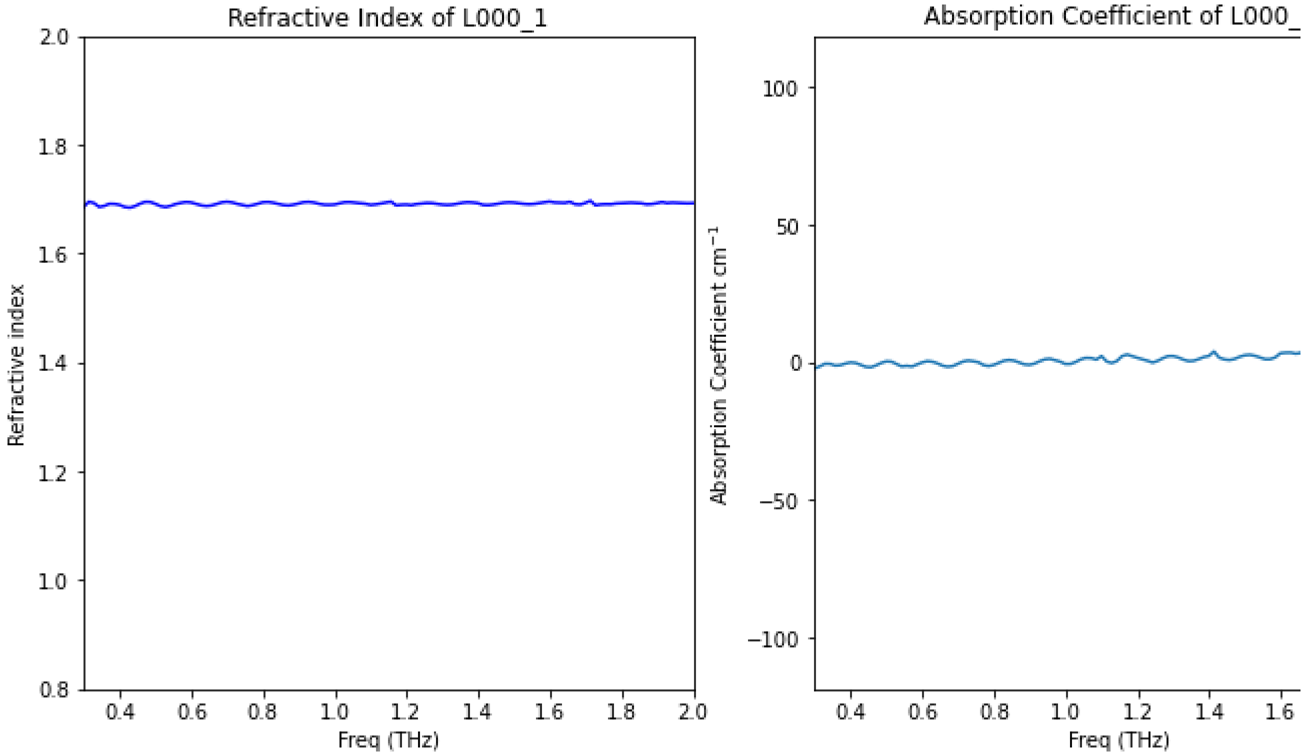


```
C:\Users\ASUS\AppData\Local\Temp/ipykernel_13884/1253449618.py:92: RuntimeWarn
  Refractive.append((((3*(10**8))*sp[i])/(2*(22/7)*((freq[i])*10**12)*((Sample
```

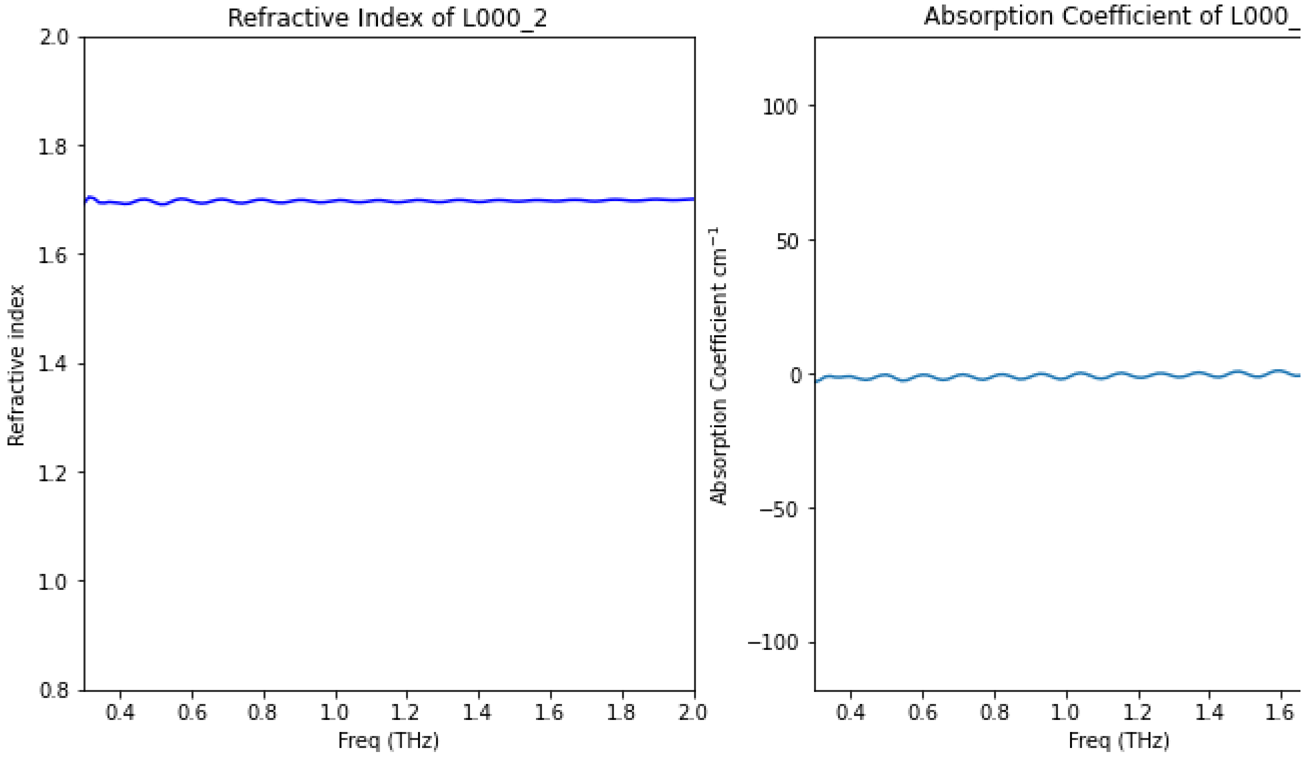


```
C:\Users\ASUS\AppData\Local\Temp/ipykernel_13884/1253449618.py:92: RuntimeWarn
  Refractive.append((((3*(10**8))*sp[i])/(2*(22/7)*((freq[i])*10**12)*((Sample
```

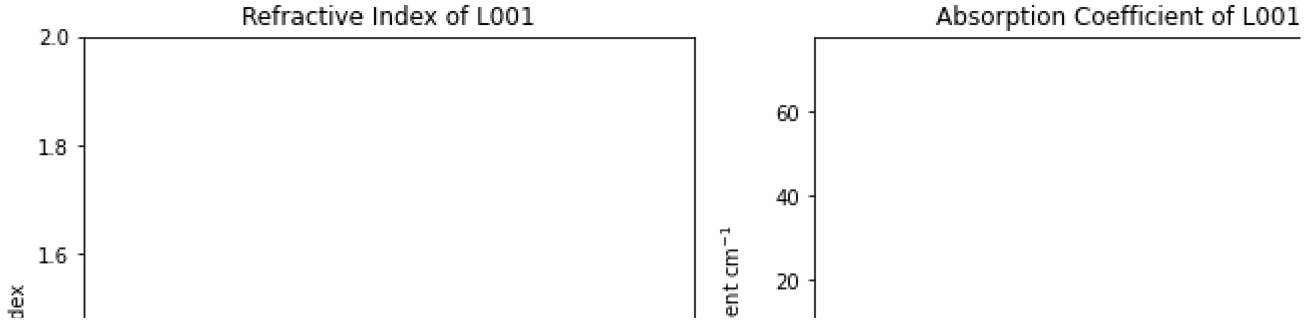

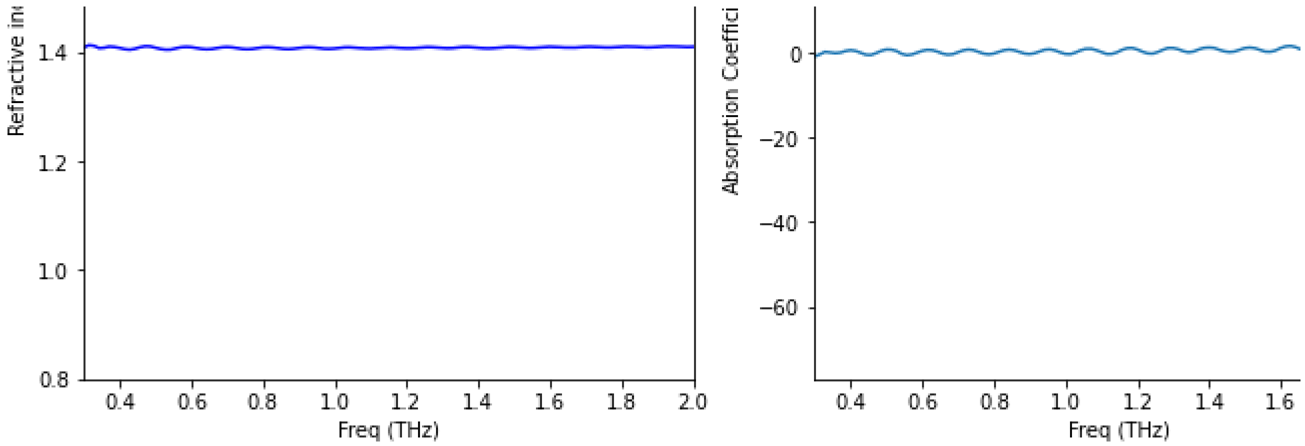


```
C:\Users\ASUS\AppData\Local\Temp/ipykernel_13884/1253449618.py:92: RuntimeWarn
  Refractive.append((((3*(10**8))*sp[i])/(2*(22/7)*((freq[i])*10**12)*((Sample
```

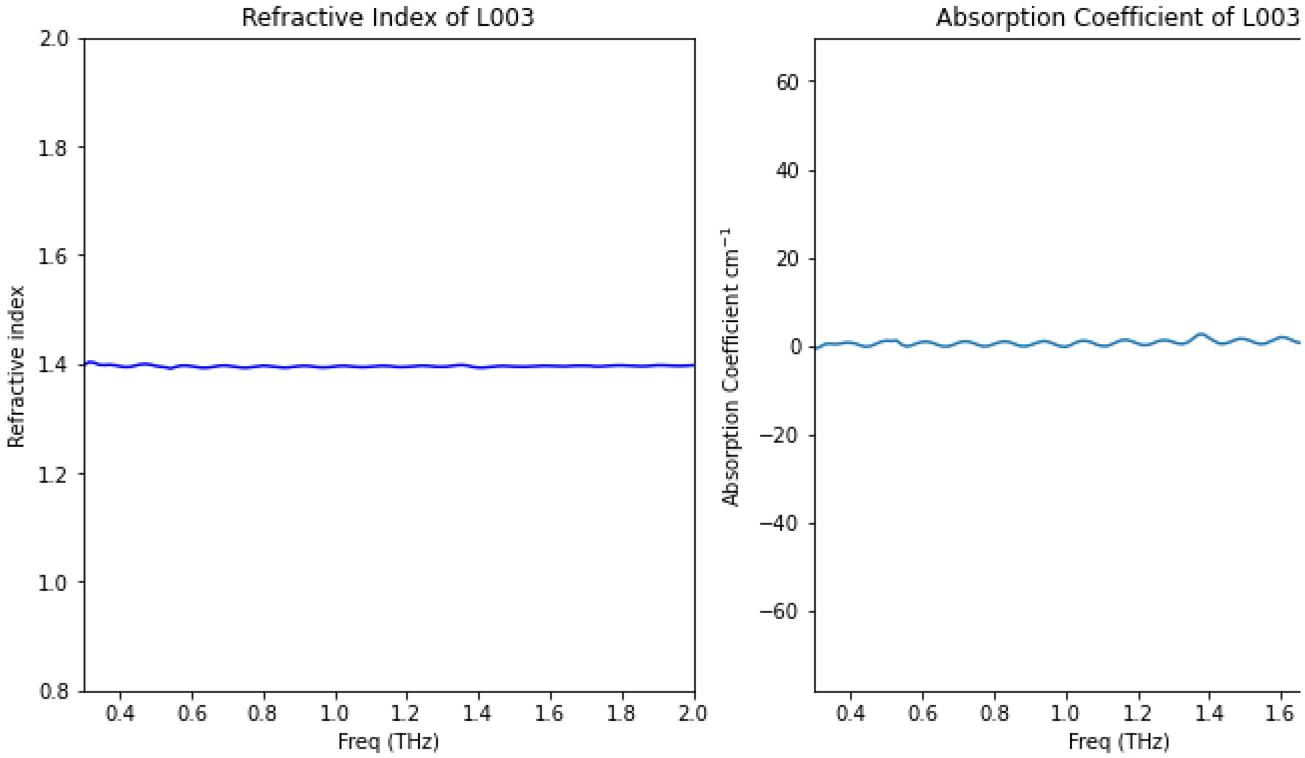


```
C:\Users\ASUS\AppData\Local\Temp/ipykernel_13884/1253449618.py:92: RuntimeWarn
  Refractive.append((((3*(10**8))*sp[i])/(2*(22/7)*((freq[i])*10**12)*((Sample
```

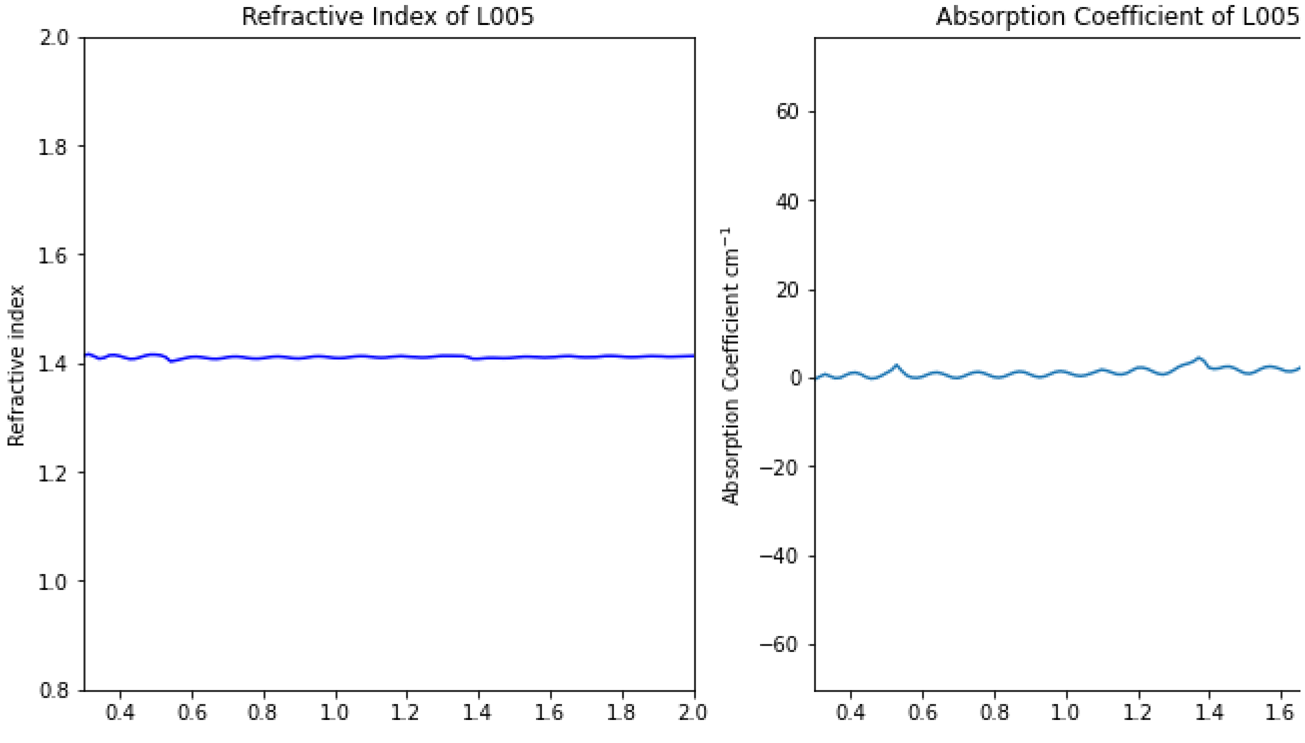

Freq (THz) Freq (THz)

```
C:\Users\ASUS\AppData\Local\Temp/ipykernel_13884/1253449618.py:92: RuntimeWarr
  Refractive.append((((3*(10**8))*sp[i])/(2*(22/7)*((freq[i])*10**12)*((Sample
```

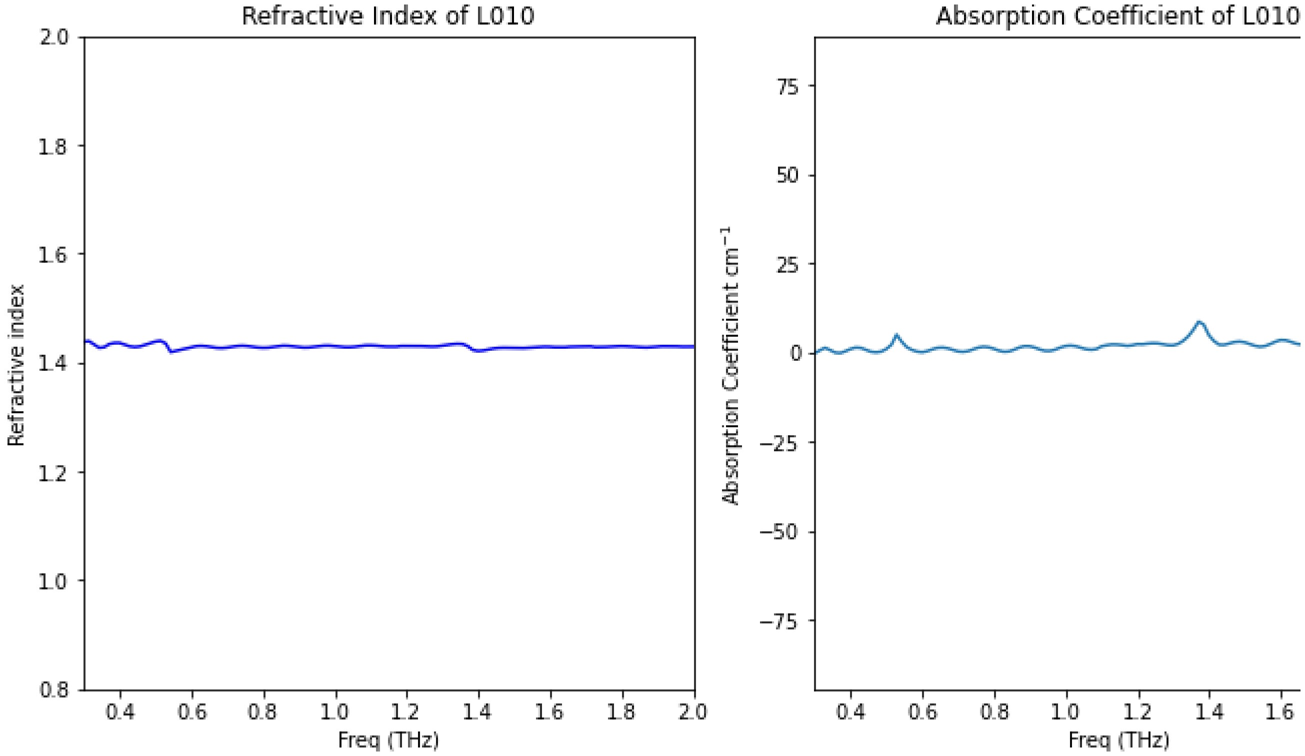


```
C:\Users\ASUS\AppData\Local\Temp/ipykernel_13884/1253449618.py:92: RuntimeWarr
  Refractive.append((((3*(10**8))*sp[i])/(2*(22/7)*((freq[i])*10**12)*((Sample
```

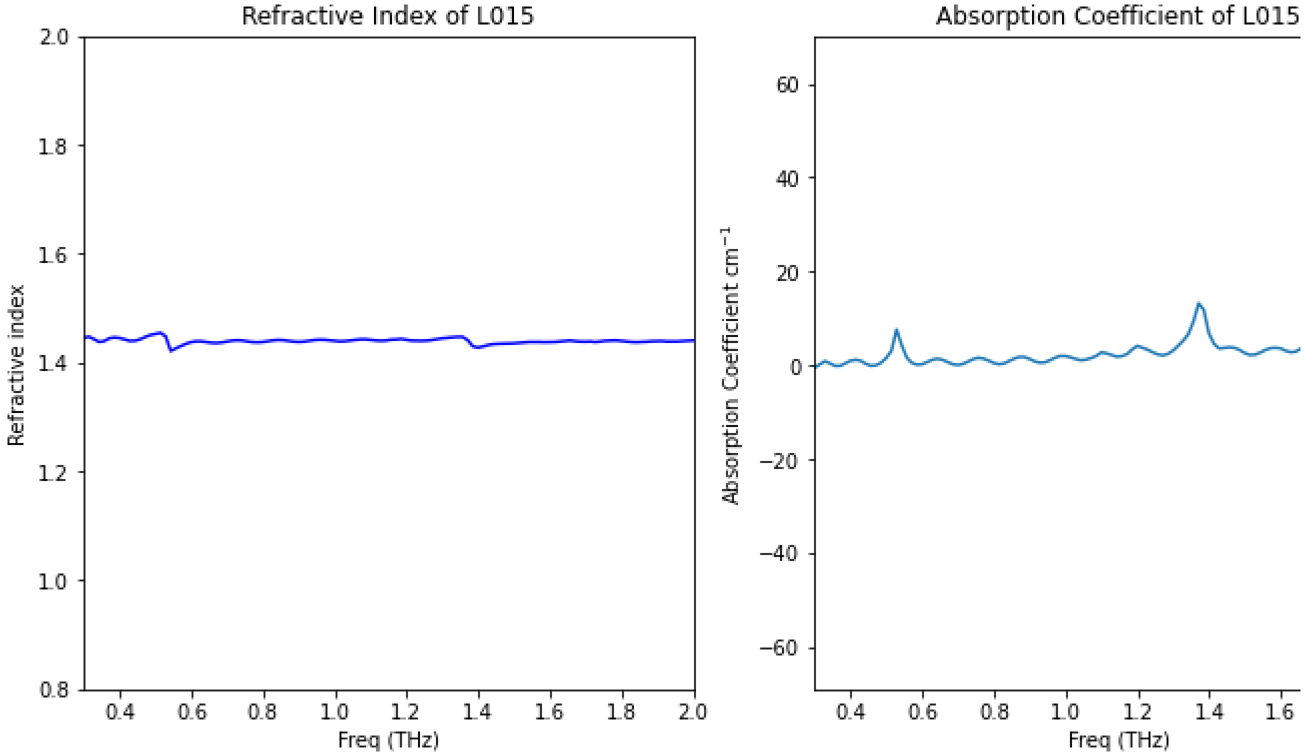


```
C:\Users\ASUS\AppData\Local\Temp/ipykernel_13884/1253449618.py:92: RuntimeWarr
  Refractive.append((((3*(10**8))*sp[i])/(2*(22/7)*((freq[i])*10**12)*((Sample
```

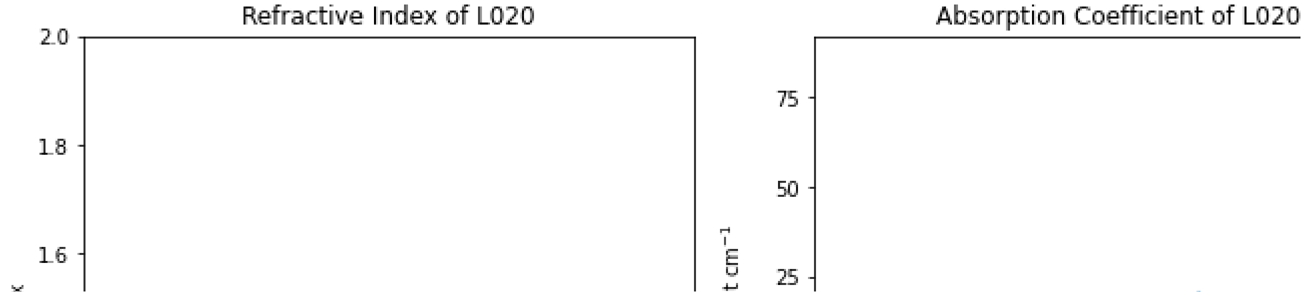

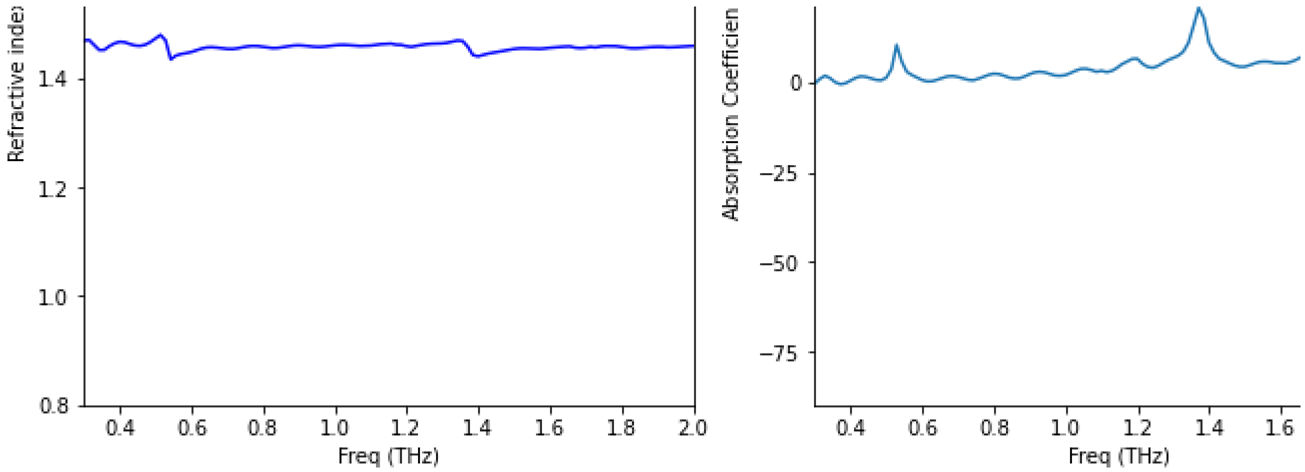


```
C:\Users\ASUS\AppData\Local\Temp/ipykernel_13884/1253449618.py:92: RuntimeWarn
  Refractive.append((((3*(10**8))*sp[i])/(2*(22/7)*((freq[i])*10**12)*((Sample
```

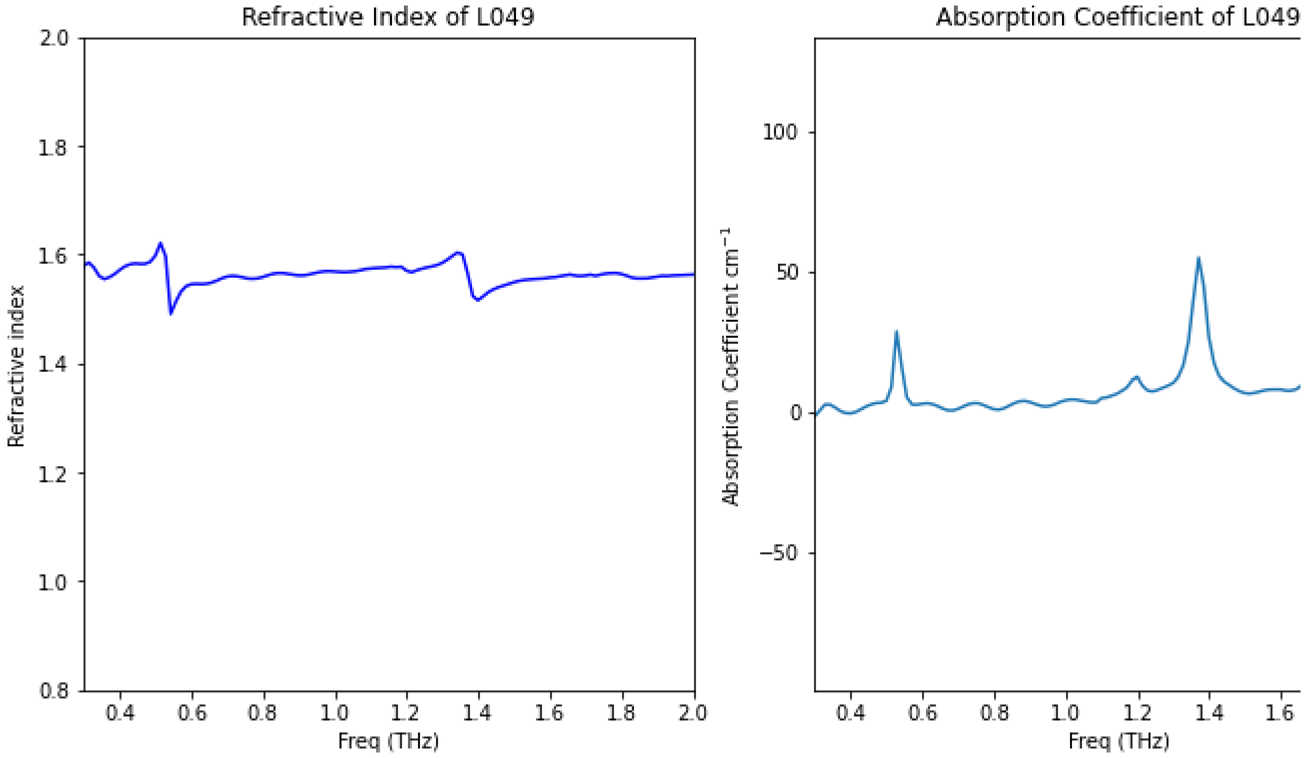


```
C:\Users\ASUS\AppData\Local\Temp/ipykernel_13884/1253449618.py:92: RuntimeWarn
  Refractive.append((((3*(10**8))*sp[i])/(2*(22/7)*((freq[i])*10**12)*((Sample
```

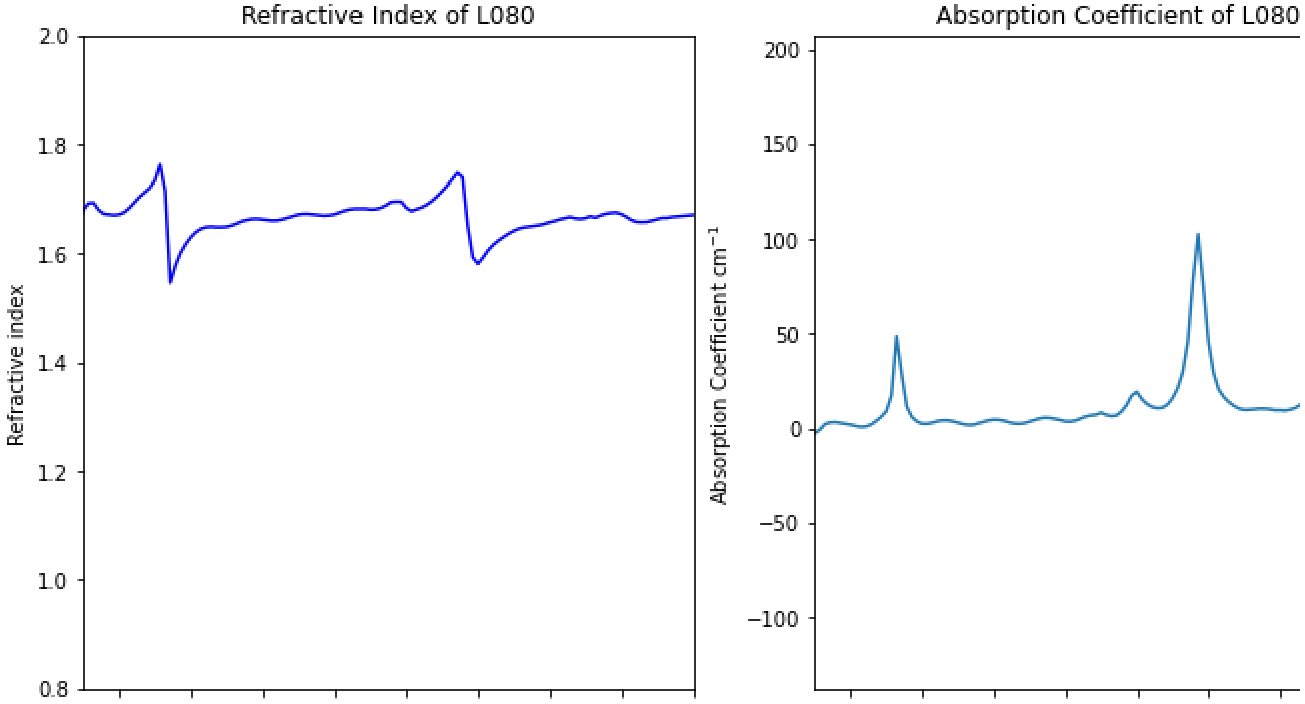

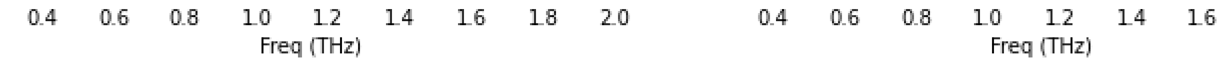


```
C:\Users\ASUS\AppData\Local\Temp/ipykernel_13884/1253449618.py:92: RuntimeWarr
  Refractive.append((((3*(10**8))*sp[i])/(2*(22/7)*((freq[i])*10**12)*((Sample
```

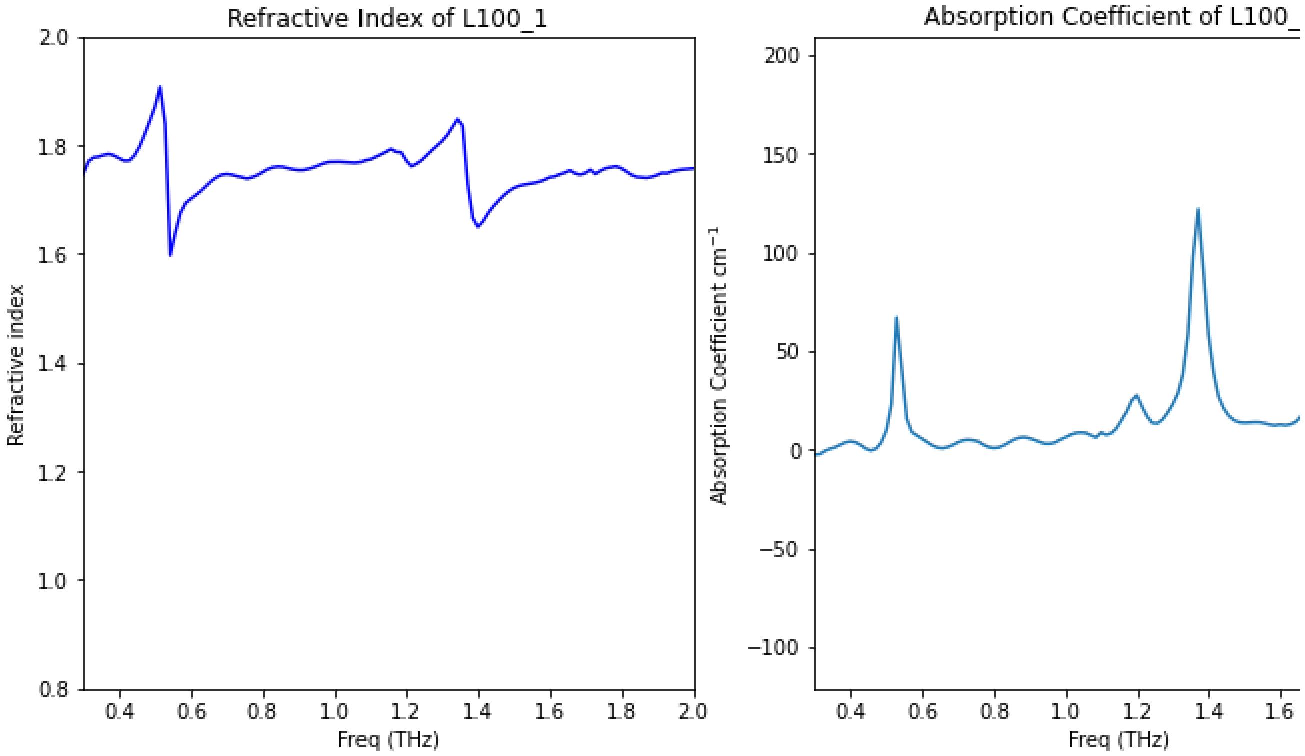


```
C:\Users\ASUS\AppData\Local\Temp/ipykernel_13884/1253449618.py:92: RuntimeWarr
  Refractive.append((((3*(10**8))*sp[i])/(2*(22/7)*((freq[i])*10**12)*((Sample
```

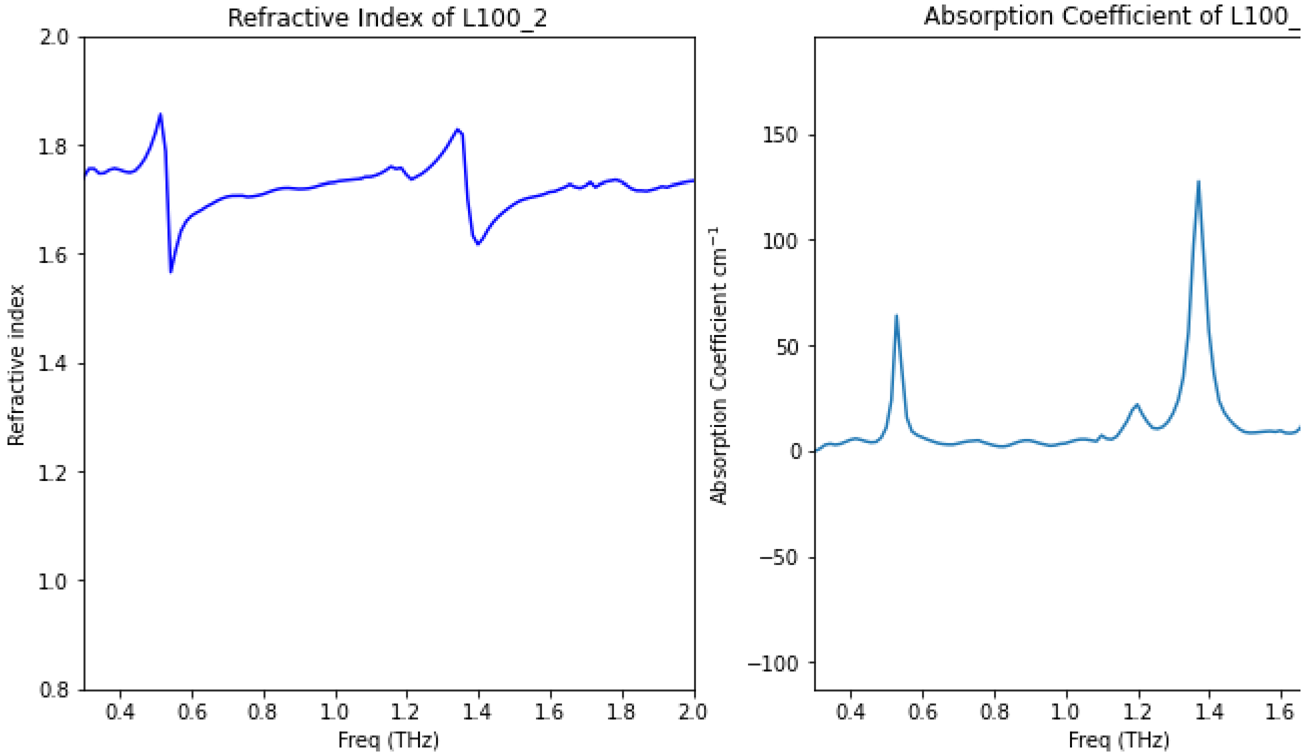


```
C:\Users\ASUS\AppData\Local\Temp/ipykernel_13884/1253449618.py:92: RuntimeWarr
  Refractive.append((((3*(10**8))*sp[i])/(2*(22/7)*((freq[i])*10**12)*((Sample
```

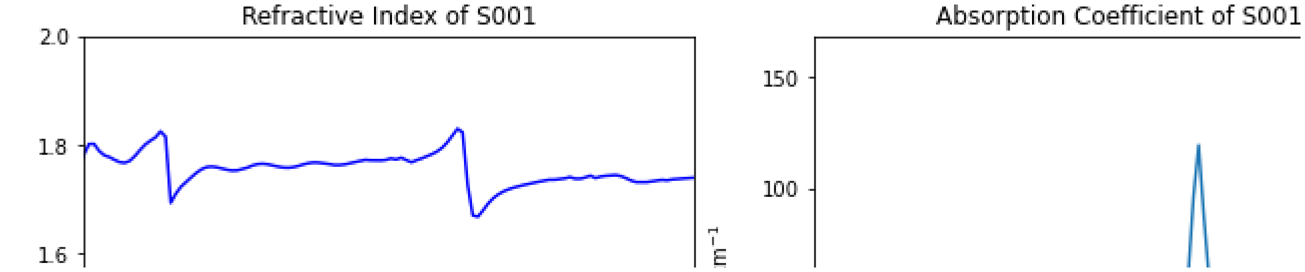

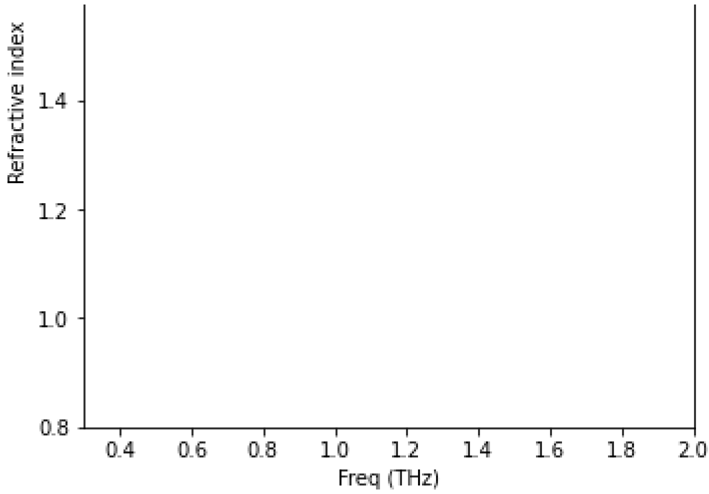

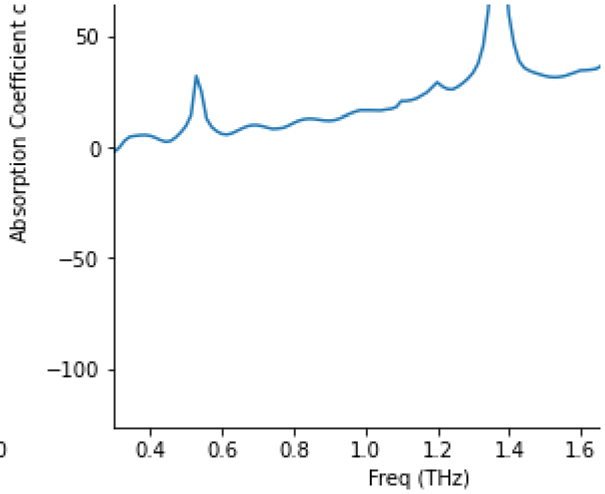


```
C:\Users\ASUS\AppData\Local\Temp/ipykernel_13884/1253449618.py:92: RuntimeWarr
  Refractive.append((((3*(10**8))*sp[i])/(2*(22/7)*((freq[i])*10**12)*((Sample
```

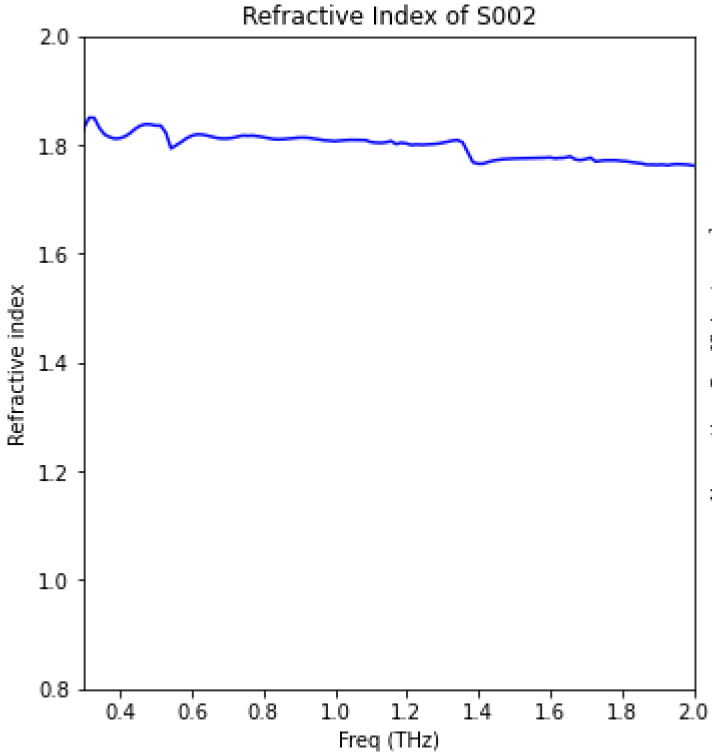

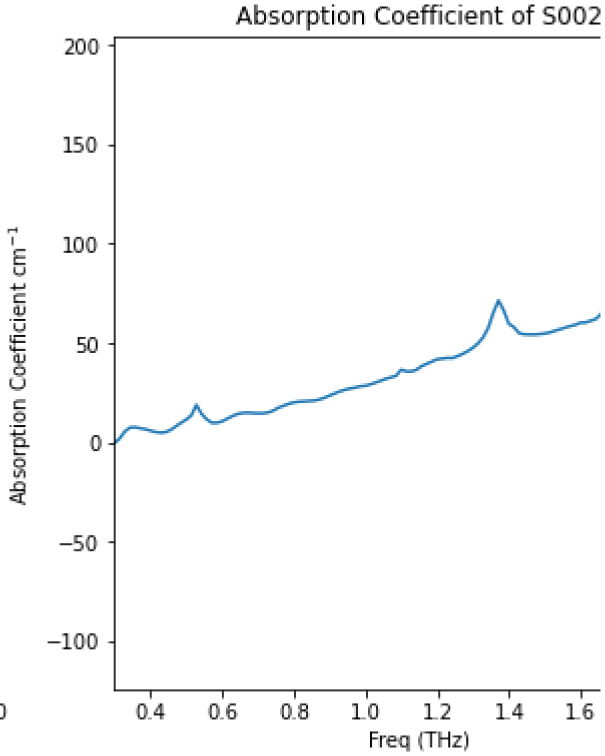


```
C:\Users\ASUS\AppData\Local\Temp/ipykernel_13884/1253449618.py:92: RuntimeWarr
  Refractive.append((((3*(10**8))*sp[i])/(2*(22/7)*((freq[i])*10**12)*((Sample
```

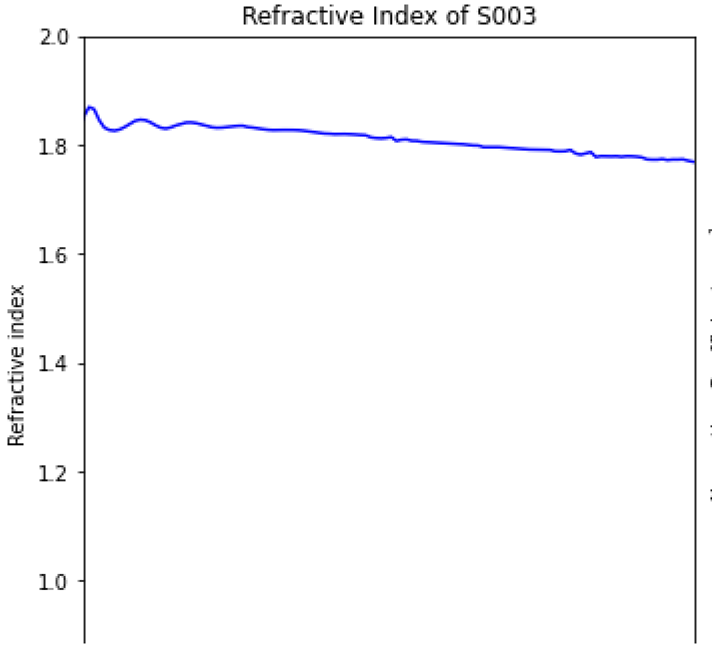

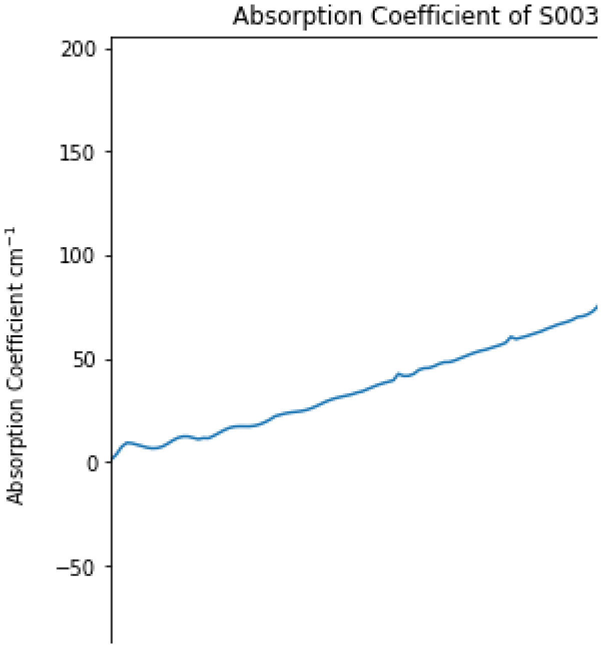

Peak area and peak height calculation

```
%matplotlib inline
import matplotlib.pyplot as plt
import matplotlib as mpl
import glob
import numpy as np
import pandas as pd
import rampy as rp
import scipy.interpolate as spi
from scipy.optimize import curve_fit

mpl.rcParams['figure.figsize'] = [6, 4]
```

```
def lorentz(x, I, gamma, x0):
    return I * gamma**2 / ((x - x0)**2 + gamma**2)

def gaussian(x, I, mu, sig):
    return I * np.exp(-(x - mu)**2 / (2 * sig**2))
```

```
peakvalues = []
for k in range(3,len(filelist)+1):
    freq = np.array(Ab[:,0])
    sig_a = np.array(Ab[:,k])

    begin_f = 0.3
    end_f = 3

    mask = (freq >= begin_f)*(freq <= end_f)
    freq = freq[mask]
    sig_a = sig_a[mask]

    enable_interpolation = False
    if enable_interpolation:
        N = 2000
        f = spi.interp1d(freq, sig_a)
        freq = np.linspace(freq[0], freq[-1], N)
        sig_a = f(freq)
        sig_a = np.array(sig_a)
    sig_a = np.reshape(sig_a, (len(sig_a), 1))

    check = []
    for i in sig_a:
        if i not in check:
            check.append(i[0])
    list=[]

    if check != [0.0]:
        corrected_poly, baseline_poly = rp.baseline(freq, sig_a, np.array([[freq[0]
        corrected_als, baseline_als = rp.baseline(freq, sig_a, np.array([[freq[0],
        corrected_arpls, baseline_arpls = rp.baseline(freq, sig_a, np.array([[freq[
```

```
        corrected_drpls, baseline_drpls = rp.baseline(freq, sig_a, np.array([[freq[

        for (begin_roi, end_roi) in [(0.46, 0.60), (1.27, 1.47)]: # center frequenc

            # remove baseline using arPLS method
            mask = (freq >= begin_roi)*(freq <= end_roi)
            corrected_absorp_fd = np.ravel(sig_a - baseline_drpls)


            popt_l, pcov_l = curve_fit(lorentz, freq[mask], corrected_absorp_fd[mas
                                    p0 = [0.1, (begin_roi + end_roi)/2, 5*(end_roi
            popt_g, pcov_g = curve_fit(gaussian, freq[mask], corrected_absorp_fd[ma
                                    p0 = [0.1, (begin_roi + end_roi)/2, (end_roi -

            lorentz_absorp_fd = lorentz(freq[mask], popt_l[0], popt_l[1], popt_l[2]
            guassian_absorp_fd = gaussian(freq[mask], popt_g[0], popt_g[1], popt_g[
            lorentz_height_fd = lorentz(popt_l[2], popt_l[0], popt_l[1], popt_l[2])
            gaussian_height_fd = gaussian(popt_g[1], popt_g[0], popt_g[1], popt_g[2

            area_numer = np.trapz(corrected_absorp_fd[mask], freq[mask])
            area_lorentz = np.trapz(lorentz_absorp_fd, freq[mask])
            area_gaussian = np.trapz(guassian_absorp_fd, freq[mask])

            list.append(area_gaussian)
            list.append(gaussian_height_fd)

            plt.axvline(x = np.min(freq[mask]), color = 'black', linestyle = '--')
            plt.axvline(x = np.max(freq[mask]), color = 'black', linestyle = '--')
            plt.scatter(freq, corrected_absorp_fd, color = 'blue', label = 'Correct
            plt.plot(freq[mask], lorentz_absorp_fd, color = 'orange', label = 'Fitt
            plt.plot(freq[mask], guassian_absorp_fd, color = 'green', label = 'Fitt
            plt.xlabel('Frequency (THz)')
            plt.ylabel('Absorption coefficient (cm$^{-1}$)')
            plt.title(filelist[k-1] + ': range = [{:.2f}, {:.2f}] THz'.format(np.mi
                        '\nArea (numerical, lorentz, gaussian) = ({:.3f}, {:.3f}, {
                        '\nHeight (lorentz, gaussian) = ({:.3f}, {:.3f})'.format(lo
            plt.xlim(begin_roi - 0.05, end_roi + 0.05)
            plt.legend()
            plt.show()
    else:
        list = ['de', 'de', 'de', 'de']

    peakvalues.append([filelist[k-1],list[0],list[2],list[1],list[3]])
```

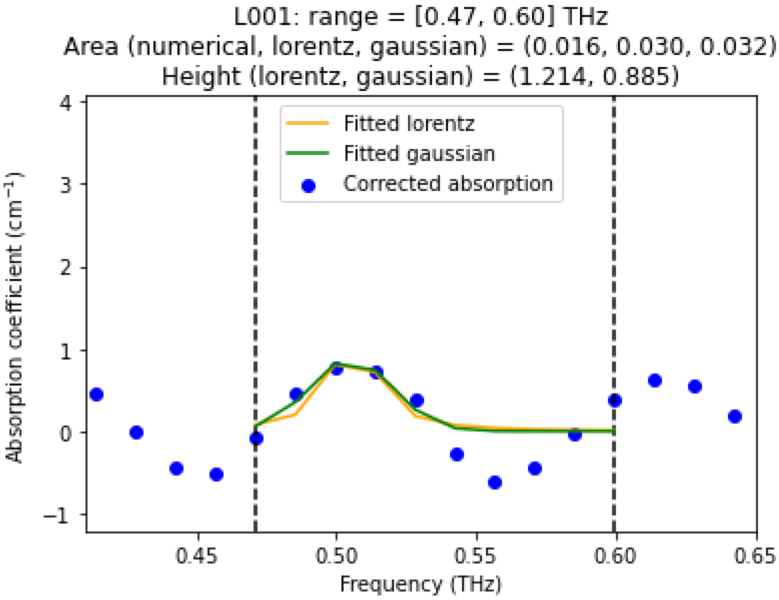
L001: range = [0.47, 0.60] THz
Area (numerical, lorentz, gaussian) = (0.016, 0.030, 0.032)
Height (lorentz, gaussian) = (1.214, 0.885)
Fitted lorentz
Fitted gaussian
Corrected absorption
Absorption coefficient (cm^{-1})
Frequency (THz)

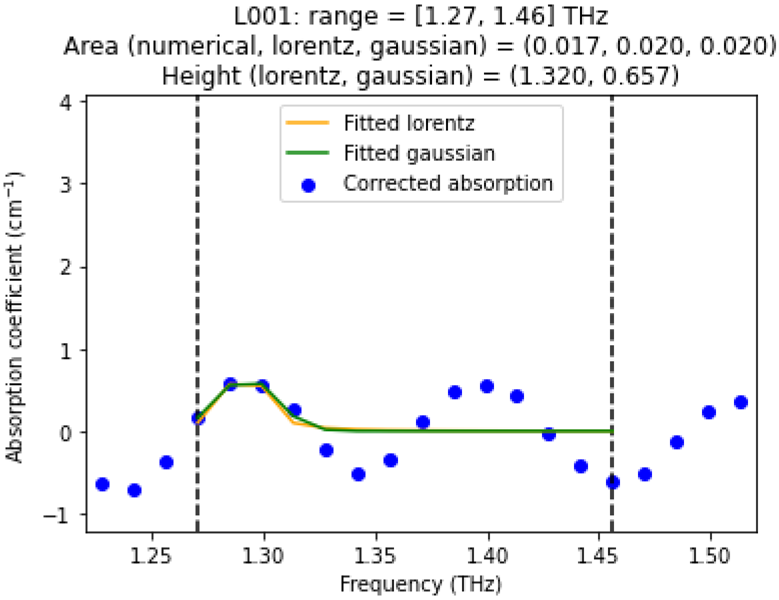
L001: range = [1.27, 1.46] THz
Area (numerical, lorentz, gaussian) = (0.017, 0.020, 0.020)
Height (lorentz, gaussian) = (1.320, 0.657)
Fitted lorentz
Fitted gaussian
Corrected absorption
Absorption coefficient (cm^{-1})
Frequency (THz)

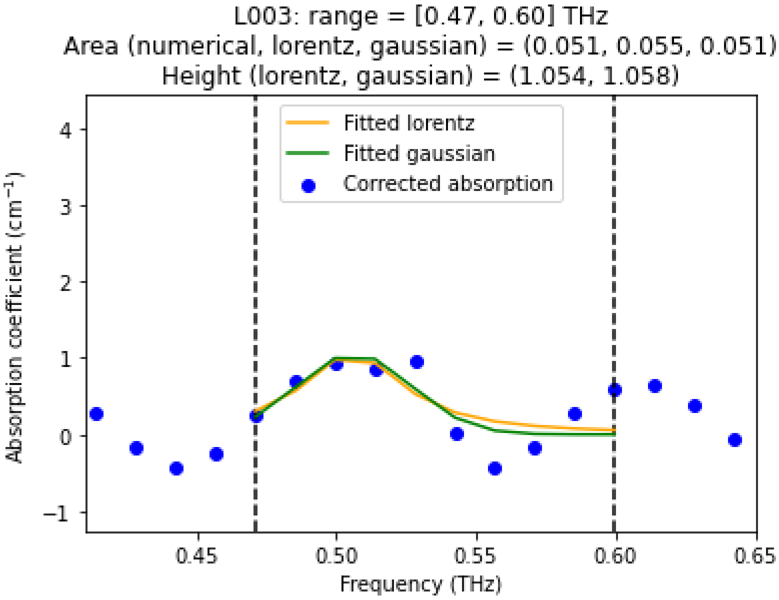
L003: range = [0.47, 0.60] THz
Area (numerical, lorentz, gaussian) = (0.051, 0.055, 0.051)
Height (lorentz, gaussian) = (1.054, 1.058)
Fitted lorentz
Fitted gaussian
Corrected absorption
Absorption coefficient (cm^{-1})
Frequency (THz)

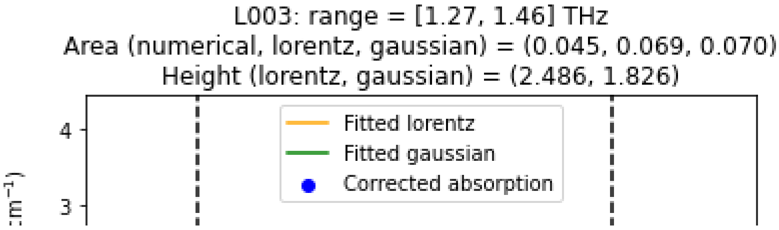
L003: range = [1.27, 1.46] THz
Area (numerical, lorentz, gaussian) = (0.045, 0.069, 0.070)
Height (lorentz, gaussian) = (2.486, 1.826)
Fitted lorentz
Fitted gaussian
Corrected absorption

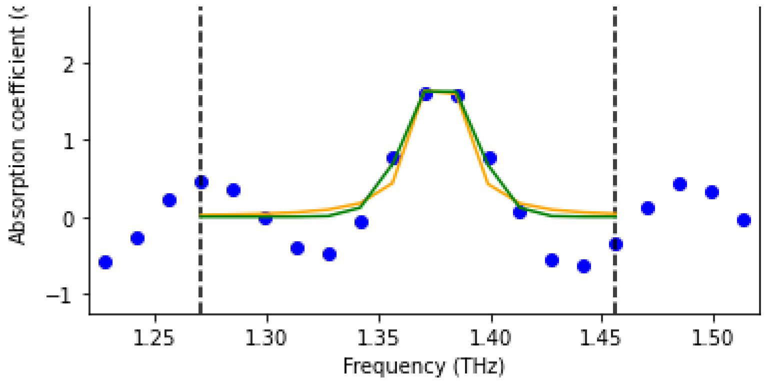

L005: range = [0.47, 0.60] THz
Area (numerical, lorentz, gaussian) = (0.073, 0.090, 0.086)
Height (lorentz, gaussian) = (2.704, 2.420)

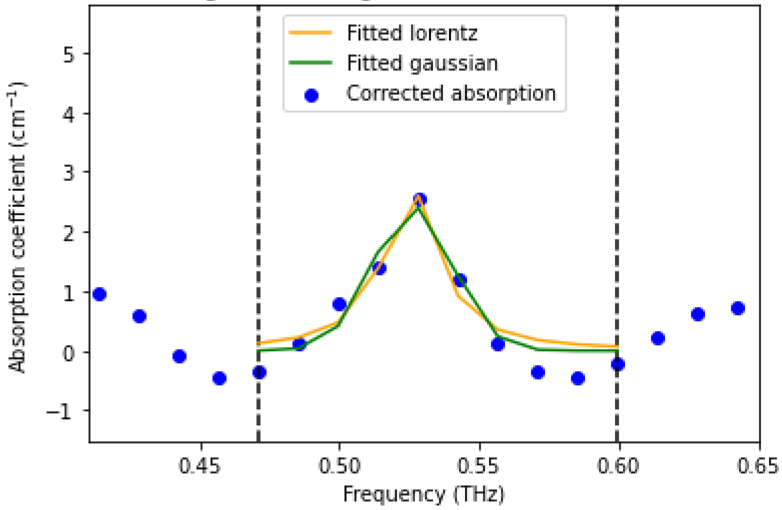

L005: range = [1.27, 1.46] THz
Area (numerical, lorentz, gaussian) = (0.162, 0.171, 0.163)
Height (lorentz, gaussian) = (2.789, 2.482)

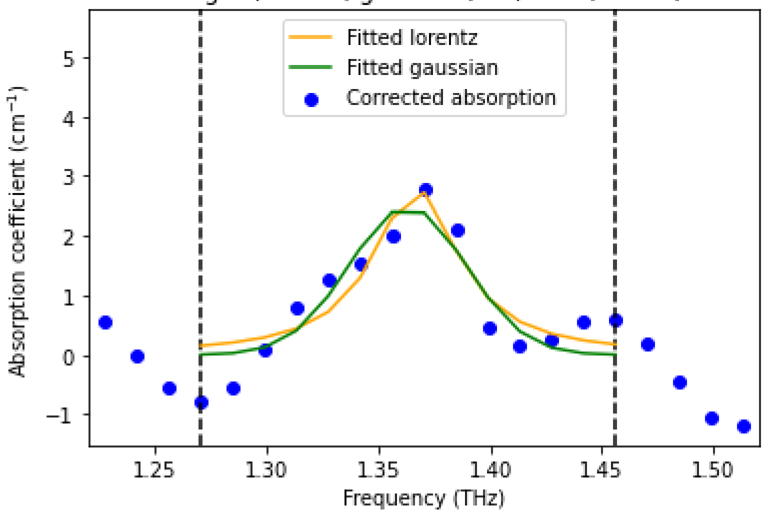

L010: range = [0.47, 0.60] THz
Area (numerical, lorentz, gaussian) = (0.129, 0.150, 0.137)
Height (lorentz, gaussian) = (4.903, 4.349)

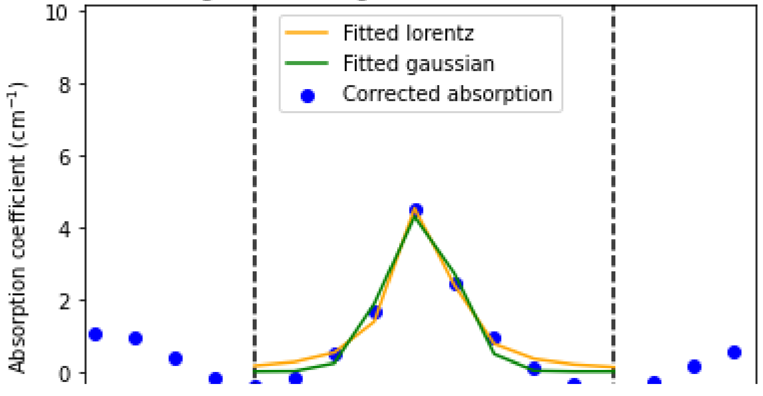

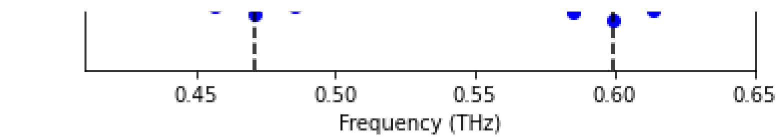

L010: range = [1.27, 1.46] THz
Area (numerical, lorentz, gaussian) = (0.292, 0.336, 0.307)
Height (lorentz, gaussian) = (6.768, 6.170)

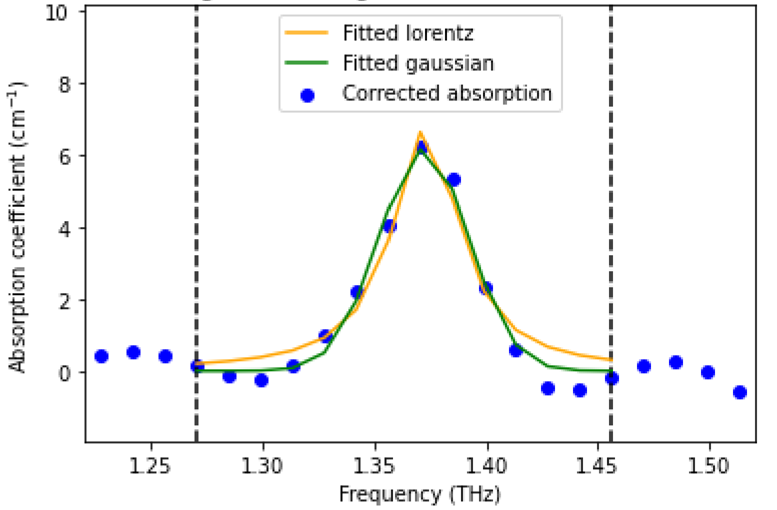

L015: range = [0.47, 0.60] THz
Area (numerical, lorentz, gaussian) = (0.222, 0.245, 0.218)
Height (lorentz, gaussian) = (7.559, 6.993)

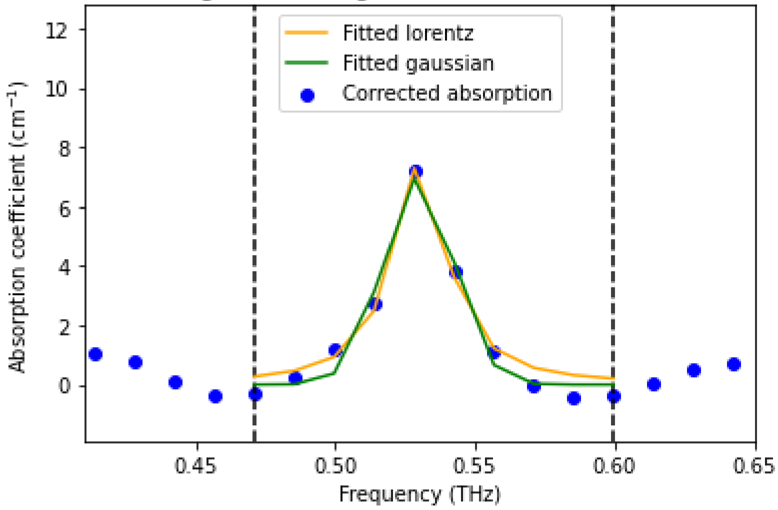

L015: range = [1.27, 1.46] THz
Area (numerical, lorentz, gaussian) = (0.576, 0.601, 0.544)
Height (lorentz, gaussian) = (10.897, 9.789)

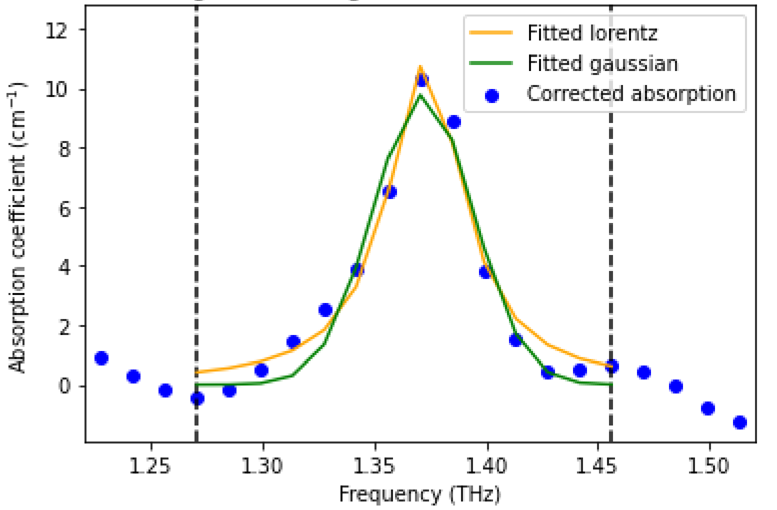

L020: range = [0.47, 0.60] THz
Area (numerical, lorentz, gaussian) = (0.354, 0.350, 0.302)
Height (lorentz, gaussian) = (10.589, 9.606)

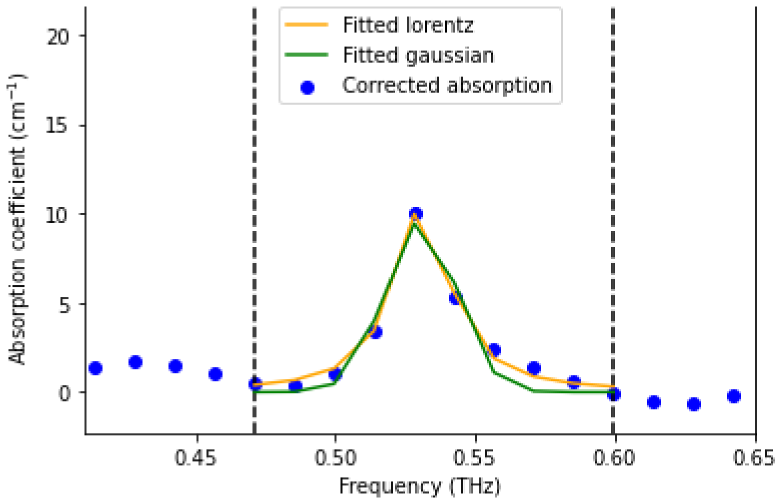

L020: range = [1.27, 1.46] THz
Area (numerical, lorentz, gaussian) = (1.000, 0.995, 0.903)
Height (lorentz, gaussian) = (16.296, 14.643)

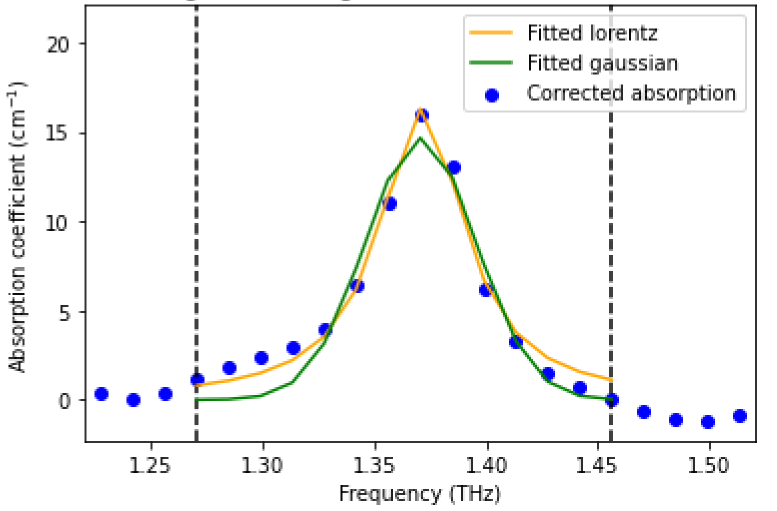

L049: range = [0.47, 0.60] THz
Area (numerical, lorentz, gaussian) = (0.950, 0.930, 0.787)
Height (lorentz, gaussian) = (30.176, 27.674)

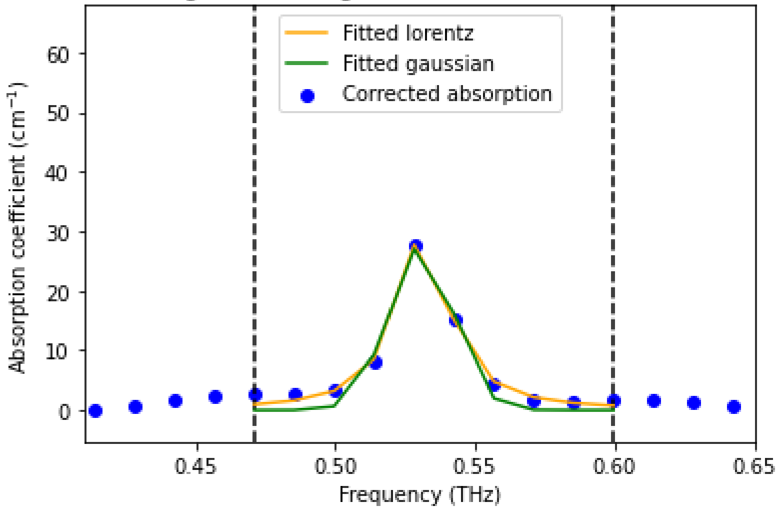

L049: range = [1.27, 1.46] THz
Area (numerical, lorentz, gaussian) = (2.827, 2.874, 2.598)
Height (lorentz, gaussian) = (48.943, 44.250)

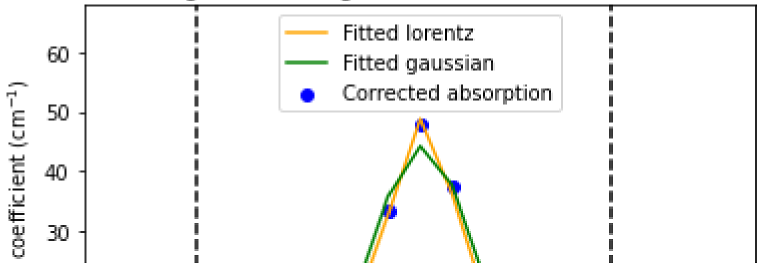

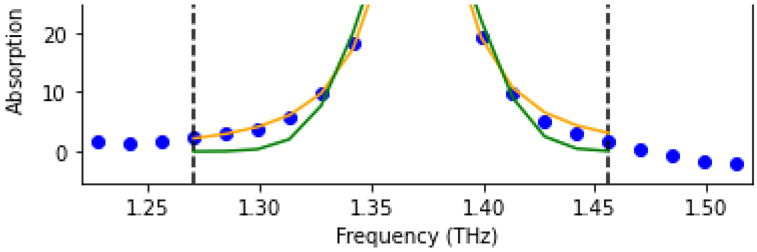

L080: range = [0.47, 0.60] THz
Area (numerical, lorentz, gaussian) = (1.687, 1.681, 1.455)
Height (lorentz, gaussian) = (49.763, 45.232)

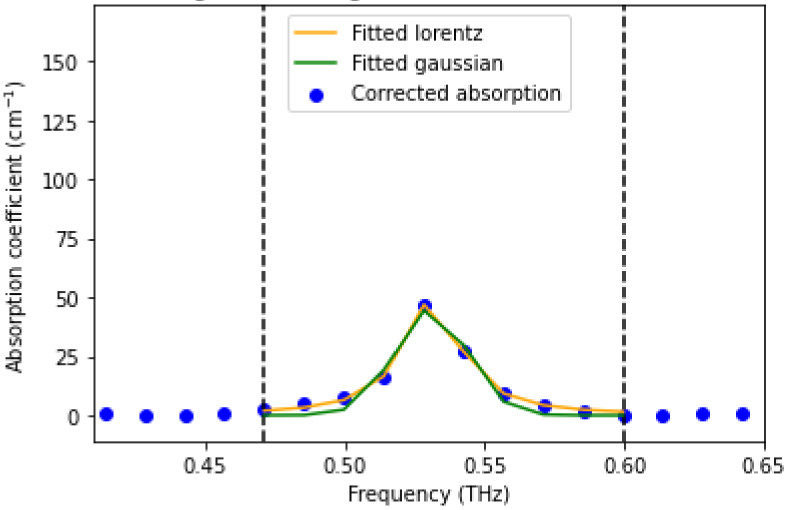

L080: range = [1.27, 1.46] THz
Area (numerical, lorentz, gaussian) = (5.541, 5.628, 5.135)
Height (lorentz, gaussian) = (94.403, 84.603)

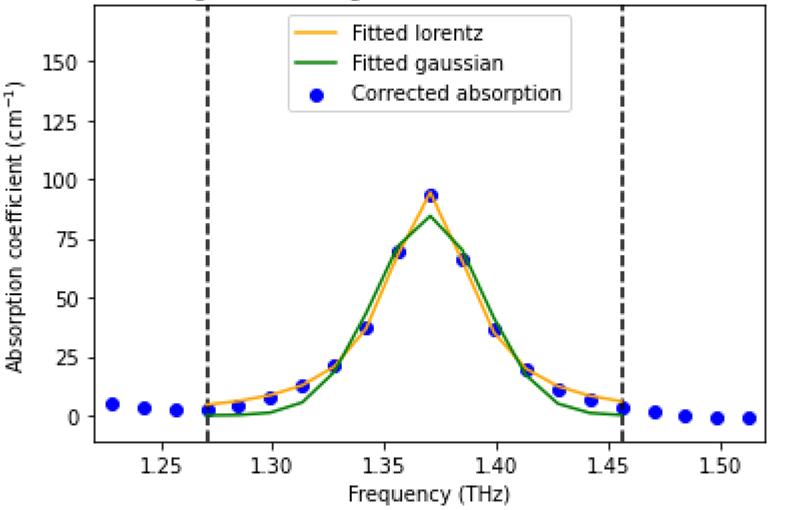

L100_1: range = [0.47, 0.60] THz
Area (numerical, lorentz, gaussian) = (2.463, 2.441, 2.137)
Height (lorentz, gaussian) = (71.978, 64.045)

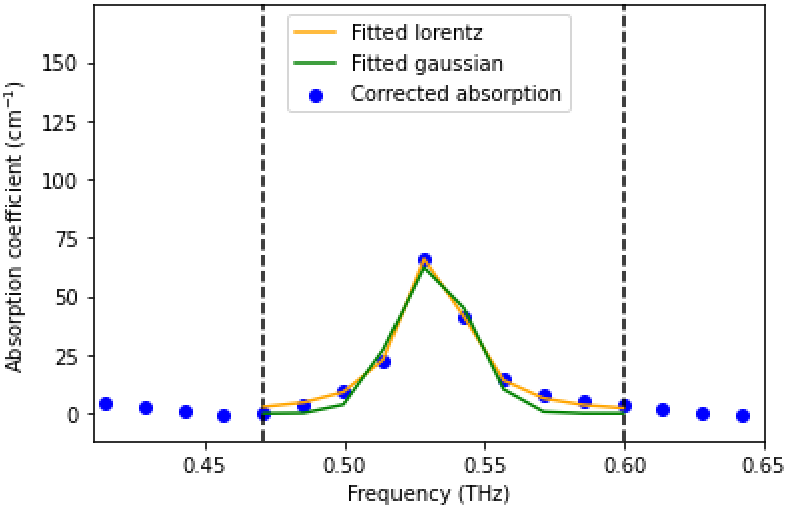

L100_1: range = [1.27, 1.46] THz
Area (numerical, lorentz, gaussian) = (7.019, 7.066, 6.512)
Height (lorentz, gaussian) = (111.559, 99.855)

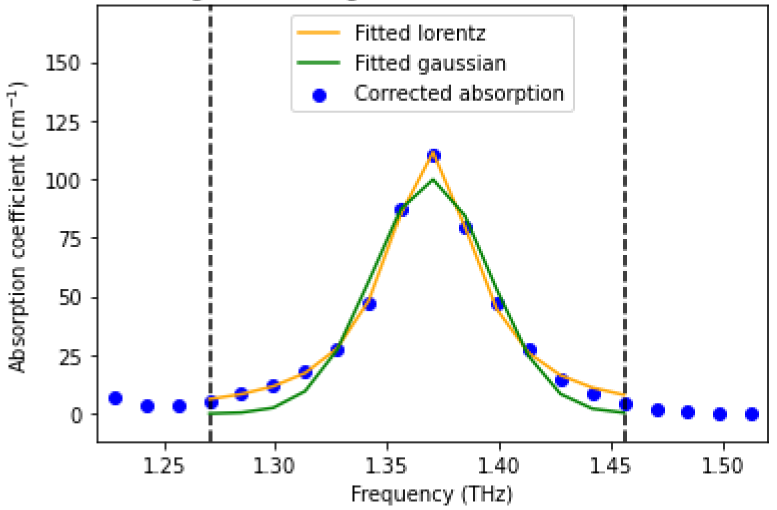

L100_2: range = [0.47, 0.60] THz
Area (numerical, lorentz, gaussian) = (2.259, 2.242, 1.950)
Height (lorentz, gaussian) = (65.850, 59.378)

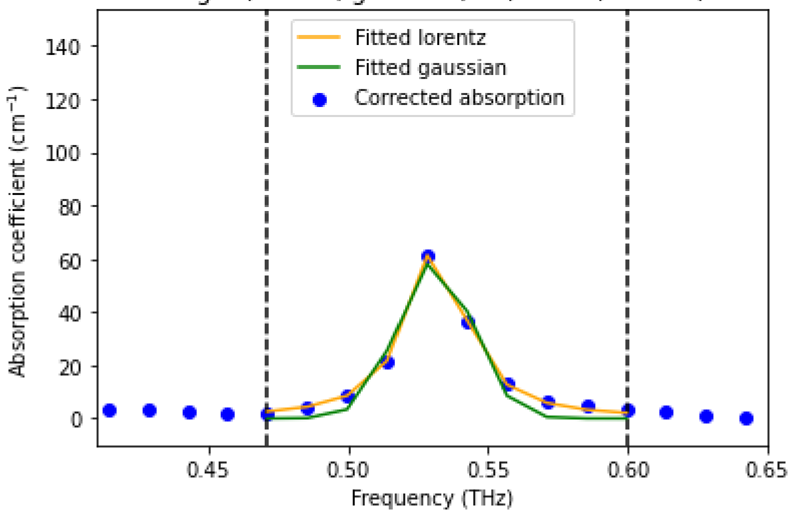

L100_2: range = [1.27, 1.46] THz
Area (numerical, lorentz, gaussian) = (7.254, 7.326, 6.722)
Height (lorentz, gaussian) = (119.969, 107.143)

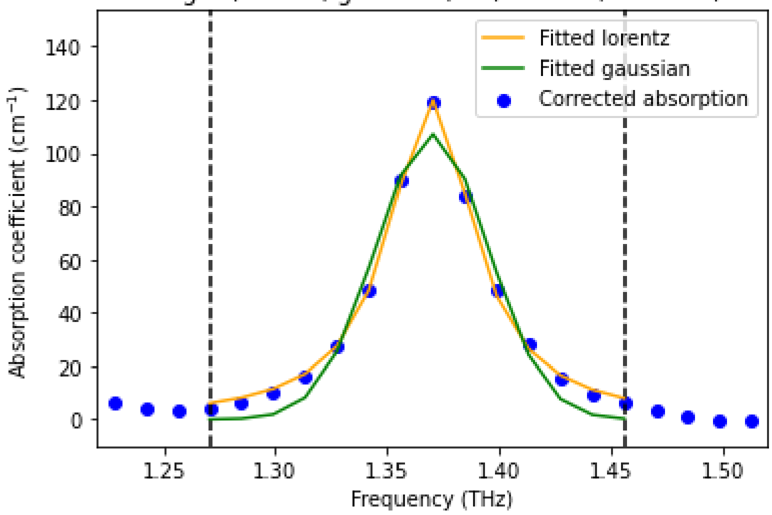

S001: range = [0.47, 0.60] THz
Area (numerical, lorentz, gaussian) = (1.413, 1.378, 1.289)
Height (lorentz, gaussian) = (30.466, 26.383)

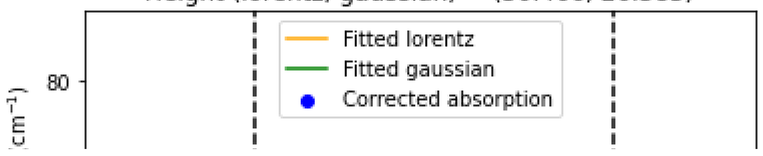

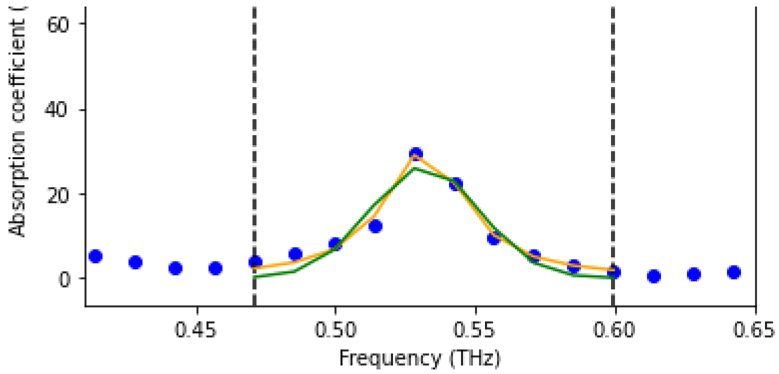

S001: range = [1.27, 1.46] THz
Area (numerical, lorentz, gaussian) = (5.254, 5.286, 4.757)
Height (lorentz, gaussian) = (92.723, 83.045)

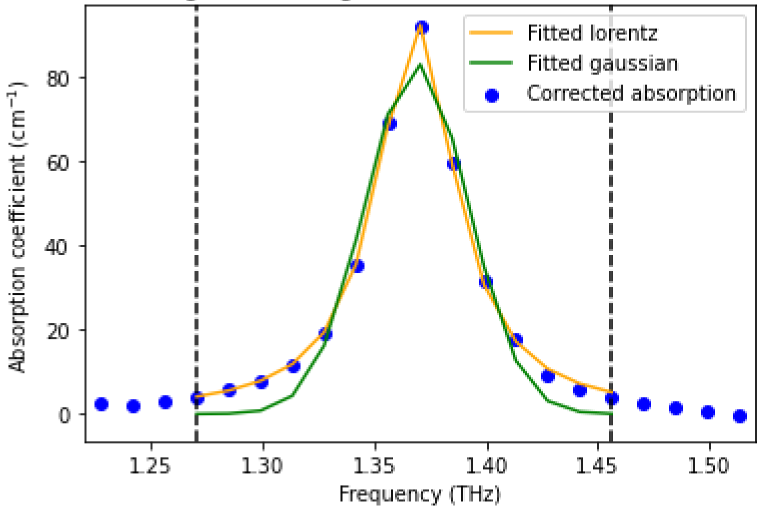

S002: range = [0.47, 0.60] THz
Area (numerical, lorentz, gaussian) = (0.575, 0.589, 0.593)
Height (lorentz, gaussian) = (10.762, 9.209)

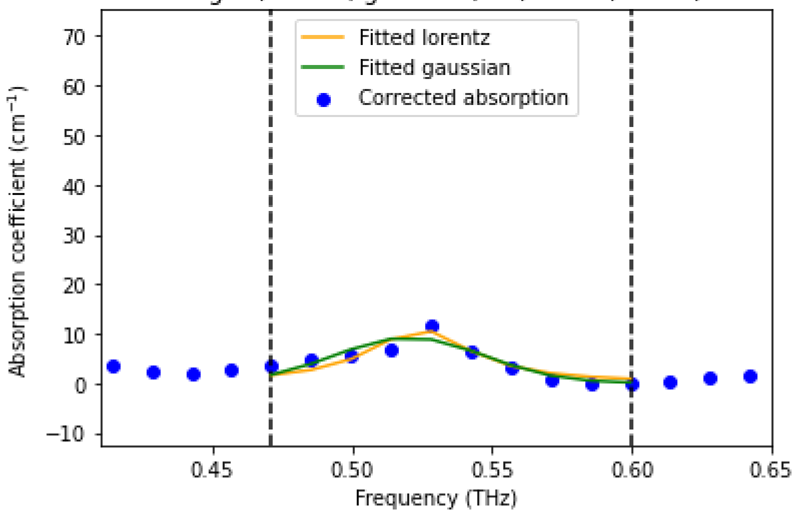

S002: range = [1.27, 1.46] THz
Area (numerical, lorentz, gaussian) = (1.349, 1.381, 1.286)
Height (lorentz, gaussian) = (22.184, 19.767)

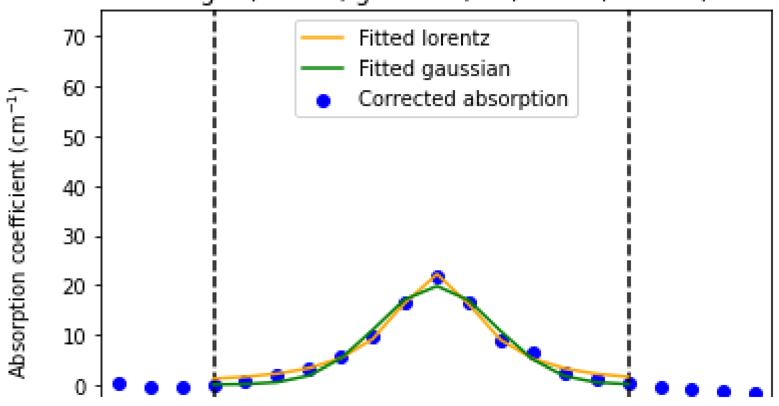

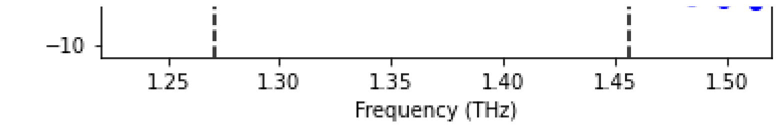

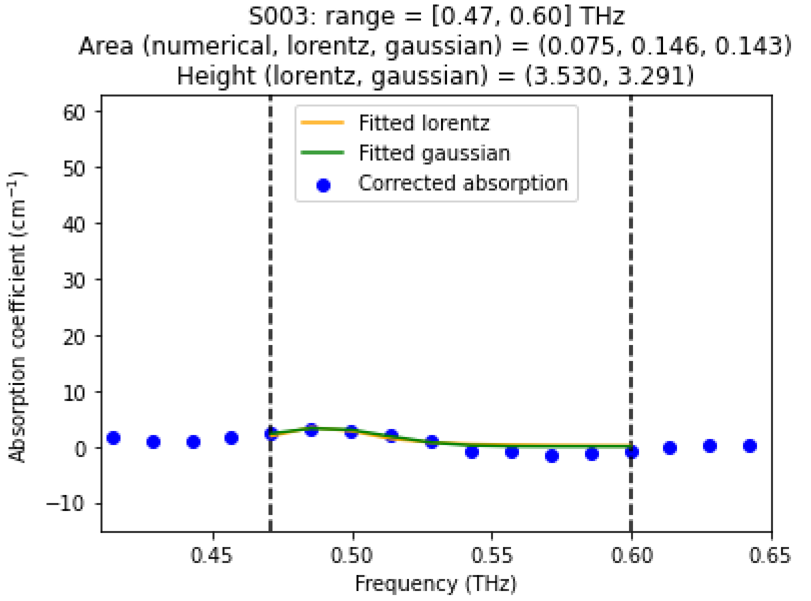

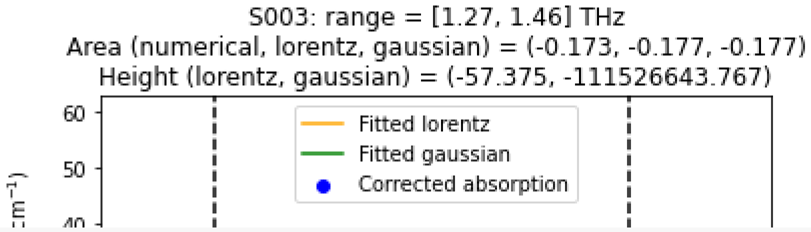


```
peakvalues1 = peakvalues[:]
df = pd.DataFrame(data = peakvalues1, columns = ['Sample','Area-0.53', 'Area-1.37',
print(df)
df.to_csv(path + "../peak_area_and_height/absorption_coefficient_spectra/peakvalues
```

```
     Sample  Area-0.53  Area-1.37  Height-0.53   Height-1.37
0      L001   0.032110   0.020167     0.884922  6.570547e-01
1      L003   0.051276   0.069635     1.057669  1.826293e+00
2      L005   0.085871   0.162569     2.420323  2.481565e+00
3      L010   0.136923   0.307355     4.349296  6.169584e+00
4      L015   0.218177   0.544224     6.992519  9.789217e+00
5      L020   0.302188   0.903281     9.606243  1.464307e+01
6      L049   0.786859   2.598000    27.673627  4.424968e+01
7      L080   1.454954   5.135207    45.231833  8.460326e+01
8    L100_1   2.136950   6.512174    64.044660  9.985540e+01
9    L100_2   1.950264   6.721744    59.378452  1.071430e+02
10     S001   1.289299   4.757484    26.383394  8.304509e+01
11     S002   0.592599   1.285798     9.209290  1.976741e+01
12     S003   0.143274  -0.176724     3.291015 -1.115266e+08
```
