# Supplementary material for: Terahertz Spectroscopic Analysis of Lactose in Infant Formula: Implications for Detection and Quantification
Source: Molecules. 2022 Aug 8;27(15):5040. doi: 10.3390/molecules27155040 (PMC9370465; doi:10.3390/molecules27155040)
Supplement: Supplementary file 1 [file molecules-27-05040-s001.zip › codes/regression.pdf]

DOI: 10.3390/molecules27155040

Prediction by absorption spectra

Plots of concentration *vs* peak area/height

In[ ]:=
```
dataset = "set2_N2_5"(*choose dataset folder here*)
path = NotebookDirectory[] <> "../data/" <>
   dataset <> "/peak_area_and_height/absorption_spectra/";
data = Import[path <> "peakvalues-drpls.csv"][[
   {1, 2, 3, 4, 5, 6, 7, 8, 9, 10, 11, 12}]];
 (*import peak area and height values*)
a053 = {}; a137 = {}; h053 = {}; h137 = {};
For[i = 1, i ≤ Length[data], i++, row = {data[[i]][[1]], data[[i]][[2]]};
 For[j = 3, j ≤ Length[data[[i]]], j++,
  (*filter out overrange values, only values in range (-0.1, 1) are included,
  values in range (-0.1, 0) are treated as 0*)
  If[0 ≤ data[[i]][[j]] < 1, AppendTo[row, data[[i]][[j]]],
   If[-0.1 < data[[i]][[j]] < 0, AppendTo[row, 0],
    AppendTo[row, "Overrange"]]]];
 If[row[[3]] ≠ "Overrange", AppendTo[a053, row[[{2, 3}]]]];
 If[row[[4]] ≠ "Overrange", AppendTo[a137, row[[{2, 4}]]]];
 If[row[[5]] ≠ "Overrange", AppendTo[h053, row[[{2, 5}]]]];
 If[row[[6]] ≠ "Overrange", AppendTo[h137, row[[{2, 6}]]]];
]
{Length[a053], Length[a137], Length[h053], Length[h137]}
```

Out[ ]= set2_N2_5

Out[ ]= {12, 12, 12, 12}

In[ ]:=
```
(*linear regression model using 0.53 THz peak area as the predictor*)
lma053 = LinearModelFit[a053, x, x]
residuals = lma053["FitResiduals"];
rmse = √(Mean[residuals^2]);
{lma053[{"ParameterTable", "RSquared", "AdjustedRSquared"}], rmse}
```

Out[ ]= FittedModel[0.00207004 + 0.000343197 x]

Out[ ]=

	Estimate	Standard Error	t-Statistic	P-Value
1	0.00207004	0.000474658	4.36112	0.00141866
x	0.000343197	9.56339×10^{-6}	35.8866	6.70476×10^{-12}

, 0.992295, 0.991524}, 0.00114949}

In[ ]:=
```
(*linear regression model using 1.37 THz peak area as the predictor*)
lma137 = LinearModelFit[a137, x, x]
residuals = lma137["FitResiduals"];
rmse = Sqrt[Mean[residuals^2]];
{lma137[{"ParameterTable", "RSquared", "AdjustedRSquared"}], rmse}
```

Out[ ]= FittedModel[0.0041026 + 0.000705698 x]

Out[ ]= {{

	Estimate	Standard Error	t-Statistic	P-Value
1	0.0041026	0.00195725	2.09611	0.0624846
x	0.000705698	0.0000394345	17.8954	6.34541×10^{-9}

, 0.96972, 0.966692}, 0.00473993}

In[ ]:=
```
(*linear regression model using 0.53 THz peak height as the predictor*)
lmh053 = LinearModelFit[h053, x, x]
residuals = lmh053["FitResiduals"];
rmse = Sqrt[Mean[residuals^2]];
{lmh053[{"ParameterTable", "RSquared", "AdjustedRSquared"}], rmse}
```

Out[ ]= FittedModel[0.084007 + 0.00654188 x]

Out[ ]= {{

	Estimate	Standard Error	t-Statistic	P-Value
1	0.084007	0.0274299	3.0626	0.0119912
x	0.00654188	0.000552657	11.8371	3.32168×10^{-7}

, 0.933386, 0.926724}, 0.066428}

In[ ]:=
```
(*linear regression model using 1.37 THz peak height as the predictor*)
lmh137 = LinearModelFit[h137, x, x]
residuals = lmh137["FitResiduals"];
rmse = Sqrt[Mean[residuals^2]];
{lmh137[{"ParameterTable", "RSquared", "AdjustedRSquared"}], rmse}
```

Out[ ]= FittedModel[0.0989789 + 0.00646105 x]

Out[ ]= {{

	Estimate	Standard Error	t-Statistic	P-Value
1	0.0989789	0.0359208	2.75548	0.0202867
x	0.00646105	0.000723731	8.92742	4.45028×10^{-6}

, 0.888516, 0.877367}, 0.0869906}

In[ ]:=
```
(*concentration vs peak area/height plots;
red for 0.53 THz peak, blue for 1.37 THz peak*)
plota053 = ListPlot[a053, PlotStyle → ■, PlotRange → {0, 0.1}];
linea053 = Plot[lma053[x], {x, 0, 100},
   PlotStyle → ■, LabelStyle → Directive[FontSize → 16, ■]];

plota137 = ListPlot[a137, PlotStyle → ■, PlotRange → {0, 0.1}];
linea137 = Plot[lma137[x], {x, 0, 100},
   PlotStyle → ■, LabelStyle → Directive[FontSize → 16, ■]];

ploth053 = ListPlot[h053, PlotStyle → ■];
lineh053 = Plot[lmh053[x], {x, 0, 100},
   PlotStyle → ■, LabelStyle → Directive[FontSize → 16, ■]];

ploth137 = ListPlot[h137, PlotStyle → ■];
lineh137 = Plot[lmh137[x], {x, 0, 100},
   PlotStyle → ■, LabelStyle → Directive[FontSize → 16, ■]];

Show[plota053, linea053, plota137,
 linea137, Frame → {{True, False}, {True, False}},
 FrameLabel → {{"Peak area (arbitrary unit)", None},
   {"Lactose concentration in % (w/w)", None}},
 FrameTicks → All, LabelStyle → Directive[FontSize → 16]]
Show[ploth053, lineh053, ploth137, lineh137,
 Frame → {{True, False}, {True, False}},
 FrameLabel → {{"Peak height (arbitrary unit)", None},
   {"Lactose concentration in % (w/w)", None}},
 FrameTicks → All, LabelStyle → Directive[FontSize → 16]]
```

Out[ ]=

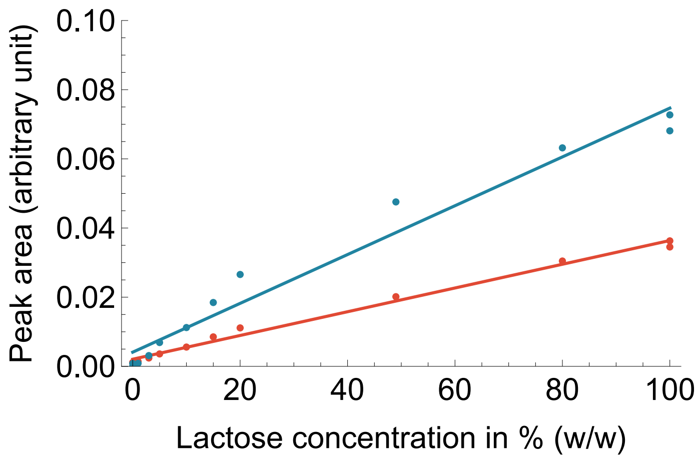

Out[•]=

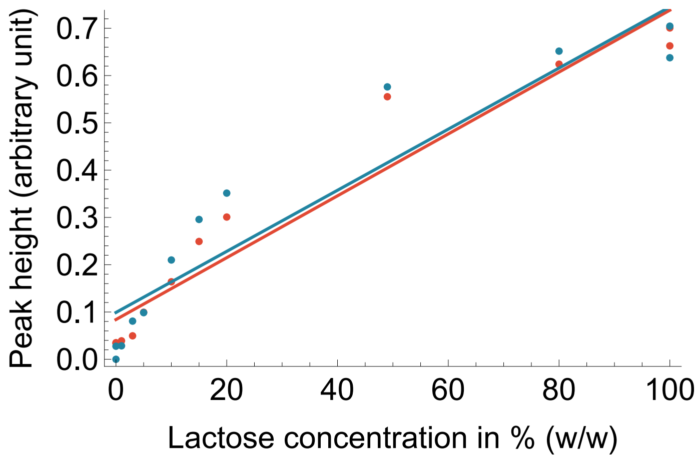

r^2, RMSE and predicted concentration

```
peak = "area-0.53";
(*choose predictor here
  ("area" or "height" of "0.53" THz or "1.37" THz peak): "area-0.53",
"area-1.37","height-0.53","height-1.37"*)
dataset = {"set1_ambient_1", "set1_N2_1", "set1_N2_2", "set2_ambient_1",
   "set2_N2_1", "set2_N2_2", "set2_N2_3", "set2_N2_4", "set2_N2_5"};
col = <|{"area-0.53" → 3, "area-1.37" → 4, "height-0.53" → 5, "height-1.37" → 6}|>;
(*column number of each predictor*)
r2tab = {};
adjr2tab = {};
rmsetab = {};
s1tab = {};
s2tab = {};
s3tab = {};
overrange = {};
For[i = 1, i ≤ Length[dataset], i++,
 path = NotebookDirectory[] <> "../data/" <> dataset[[i]] <> "/";
 method = {"poly", "als", "arpls", "drpls"};
 (*baseline correction methods, performed in data-processing.ipynb*)
 result = {}; s1 = {}; s2 = {}; s3 = {};
 For[j = 1, j ≤ Length[method], j++,
  data0 = Import[path <> "peak_area_and_height/absorption_spectra/peakvalues-" <>
     method[[j]] <> ".csv"];
  (*import peak area and peak height values*)
  cdata = data0[[1 ;; 12]];
  (*calibration data of pure lactose standards*)
  (*filter out overrange values, only values in range (-0.1, 1) are included,
  values in range (-0.1, 0) are treated as 0*)
  peakvalue = {};
  For[k = 1, k ≤ Length[cdata], k++, y = cdata[[k]][[col[peak]]];
   If[0 ≤ y < 1, AppendTo[peakvalue, {cdata[[k]][[2]], y}],
    If[-0.1 < y < 0, AppendTo[peakvalue, {cdata[[k]][[2]], 0}],
```

```
      AppendTo[overrange, {dataset⟦i⟧, method⟦j⟧, cdata⟦k⟧}]]]];
    lm = LinearModelFit[peakvalue, x, x];
    (*linear regression model*)
    trendline = Normal[lm]; (*equation of trendline*)
    residuals = lm["FitResiduals"];
    r2 = lm["RSquared"];
    adjr2 = lm["AdjustedRSquared"];
    AppendTo[result, {trendline, NumberForm[r2, {5, 4}],
      NumberForm[adjr2, {5, 4}], NumberForm[rmse = Sqrt[Mean[residuals^2]], {6, 5}]}];
    sdata = data0⟦{13, 14, 15}⟧;
    prediction = {};
    (*data of milk powder samples*)
    For[l = 1, l ≤ Length[sdata], l++, peakval = sdata⟦l⟧⟦col[peak]⟧;
     sol = Solve[trendline == peakval, x]⟦1⟧;
     (*solve for concentration from peak area or height*)
     AppendTo[prediction, If[-0.1 < peakval < 1, NumberForm[conc = x /. sol, {3, 1}],
       "nd"](*overrange peak values are reported as "nd" for not detected*)
     ]];
    AppendTo[s1, prediction⟦1⟧];
    AppendTo[s2, prediction⟦2⟧];
    AppendTo[s3, prediction⟦3⟧];
   ];
  AppendTo[r2tab, result⟦All, 2⟧];
  AppendTo[adjr2tab, result⟦All, 3⟧];
  AppendTo[rmsetab, result⟦All, 4⟧];
  AppendTo[s1tab, s1];
  AppendTo[s2tab, s2];
  AppendTo[s3tab, s3];
 ]
overrange(*excluded overrange values*)
TableForm[r2tab, TableHeadings → {dataset, method}](*table of r^2 values*)
TableForm[rmsetab, TableHeadings → {dataset, method}](*table of RMSE values*)
TableForm[s1tab, TableHeadings → {dataset, method}]
(*table of predicted concentration of sample 1*)
TableForm[s2tab, TableHeadings → {dataset, method}]
(*table of predicted concentration of sample 2*)
TableForm[s3tab, TableHeadings → {dataset, method}]
(*table of predicted concentration of sample 3*)
```

Out[•]= { }

Out[ ]//TableForm=

	poly	als	arpls	drpls
set1_ambient_1	0.9846	0.9921	0.9905	0.9892
set1_N2_1	0.9776	0.9757	0.9881	0.9861
set1_N2_2	0.9701	0.9809	0.9655	0.9744
set2_ambient_1	0.9803	0.9979	0.9958	0.9960
set2_N2_1	0.9719	0.9659	0.9778	0.9666
set2_N2_2	0.9733	0.9797	0.9735	0.9716
set2_N2_3	0.9766	0.9636	0.9462	0.9482
set2_N2_4	0.9813	0.9766	0.9794	0.9827
set2_N2_5	0.9773	0.9913	0.9883	0.9923

Out[ ]//TableForm=

	poly	als	arpls	drpls
set1_ambient_1	0.00112	0.00116	0.00107	0.00116
set1_N2_1	0.00138	0.00213	0.00133	0.00152
set1_N2_2	0.00162	0.00197	0.00247	0.00213
set2_ambient_1	0.00139	0.00066	0.00072	0.00071
set2_N2_1	0.00143	0.00232	0.00169	0.00218
set2_N2_2	0.00152	0.00201	0.00209	0.00225
set2_N2_3	0.00143	0.00246	0.00288	0.00283
set2_N2_4	0.00126	0.00201	0.00178	0.00167
set2_N2_5	0.00140	0.00126	0.00133	0.00115

Out[ ]//TableForm=

	poly	als	arpls	drpls
set1_ambient_1	44.7	41.3	43.4	40.3
set1_N2_1	48.2	34.8	49.3	38.8
set1_N2_2	48.7	35.3	47.9	37.3
set2_ambient_1	50.3	43.6	43.1	38.5
set2_N2_1	47.6	32.9	49.3	41.2
set2_N2_2	49.1	36.1	48.3	38.5
set2_N2_3	49.9	35.2	50.0	40.8
set2_N2_4	49.4	36.9	50.2	41.5
set2_N2_5	49.0	36.2	52.5	43.2

Out[ ]//TableForm=

	poly	als	arpls	drpls
set1_ambient_1	6.8	12.1	7.6	-12.8
set1_N2_1	11.4	4.9	8.8	2.6
set1_N2_2	11.7	3.3	10.1	0.5
set2_ambient_1	14.3	13.6	8.9	5.2
set2_N2_1	11.4	2.0	9.3	1.9
set2_N2_2	11.9	3.6	8.5	1.1
set2_N2_3	13.5	-1.0	8.0	0.8
set2_N2_4	12.3	4.4	9.8	2.6
set2_N2_5	12.9	5.3	11.7	3.3

Out[ ]//TableForm=

	poly	als	arpls	drpls
set1_ambient_1	-5.0	1.9	-14.5	-20.8
set1_N2_1	-3.0	-8.4	-12.4	-19.1
set1_N2_2	-2.5	-9.0	-12.0	-20.2
set2_ambient_1	-10.6	2.8	-12.8	-19.4
set2_N2_1	-3.3	-11.2	-12.9	-19.8
set2_N2_2	-3.3	-11.3	-14.8	-20.3
set2_N2_3	-3.1	-15.0	-13.7	-23.0
set2_N2_4	-3.6	-8.8	-13.0	-20.3
set2_N2_5	-2.9	-8.8	-12.3	-18.8

Prediction by absorption coefficient spectra

Plots of concentration *vs* peak area/height

In[ ]:=
```
dataset = "set2_N2_5"(*choose dataset folder here*)
path = NotebookDirectory[] <> "../data/" <> dataset <>
   "/peak_area_and_height/absorption_coefficient_spectra/";
data = Import[path <> "peakvalues-drpls.csv"][[
   {1, 2, 3, 4, 5, 6, 7, 8, 9, 10, 11, 12}]];
 (*import peak area and height values*)
a053 = {}; a137 = {}; h053 = {}; h137 = {};
For[i = 1, i ≤ Length[data], i++, row = {data[[i]][[1]], data[[i]][[2]]};
 For[j = 3, j ≤ Length[data[[i]]], j++,
  (*filter out overrange values, only values ≥ 0 are included,
  values in range (-0.1, 0) are treated as 0*)
  If[data[[i]][[j]] ≥ 0, AppendTo[row, data[[i]][[j]]],
   If[data[[i]][[j]] < -0.1, AppendTo[row, 0],
    AppendTo[row, "Overrange"]]]];
 If[row[[3]] ≠ "Overrange", AppendTo[a053, row[[{2, 3}]]]];
 If[row[[4]] ≠ "Overrange", AppendTo[a137, row[[{2, 4}]]]];
 If[row[[5]] ≠ "Overrange", AppendTo[h053, row[[{2, 5}]]]];
 If[row[[6]] ≠ "Overrange", AppendTo[h137, row[[{2, 6}]]]];
]
{Length[a053], Length[a137], Length[h053], Length[h137]}
```

Out[ ]= set2_N2_5

Out[ ]= {12, 11, 12, 12}

In[∘]:=
```
(*linear regression model using 0.53 THz peak area as the predictor*)
lma053 = LinearModelFit[a053, x, x]
residuals = lma053["FitResiduals"];
rmse = Sqrt[Mean[residuals^2]];
{lma053[{"ParameterTable", "RSquared", "AdjustedRSquared"}], rmse}
```

Out[∘]= FittedModel[$-0.0288061 + 0.0197523\, x$]

Out[∘]=

	Estimate	Standard Error	t-Statistic	P-Value
1	−0.0288061	0.0346739	−0.830771	0.425494
x	0.0197523	0.000698609	28.2737	7.12066×10^{-11}

, 0.987645, 0.98641}, 0.083971}

In[∘]:=
```
(*linear regression model using 1.37 THz peak area as the predictor*)
lma137 = LinearModelFit[a137, x, x]
residuals = lma137["FitResiduals"];
rmse = Sqrt[Mean[residuals^2]];
{lma137[{"ParameterTable", "RSquared", "AdjustedRSquared"}], rmse}
```

Out[∘]= FittedModel[$-0.246804 + 0.0670845\, x$]

Out[∘]=

	Estimate	Standard Error	t-Statistic	P-Value
1	−0.246804	0.0957133	−2.57858	0.0297659
x	0.0670845	0.00184633	36.334	4.48727×10^{-11}

, 0.993229, 0.992476}, 0.212733}

In[∘]:=
```
(*linear regression model using 0.53 THz peak height as the predictor*)
lmh053 = LinearModelFit[h053, x, x]
residuals = lmh053["FitResiduals"];
rmse = Sqrt[Mean[residuals^2]];
{lmh053[{"ParameterTable", "RSquared", "AdjustedRSquared"}], rmse}
```

Out[∘]= FittedModel[$-0.679353 + 0.607991\, x$]

Out[∘]=

	Estimate	Standard Error	t-Statistic	P-Value
1	−0.679353	0.777951	−0.873259	0.403005
x	0.607991	0.0156741	38.7894	3.09585×10^{-12}

, 0.993398, 0.992737}, 1.88399}

In[∘]:=
```
(*linear regression model using 1.37 THz peak height as the predictor*)
lmh137 = LinearModelFit[h137, x, x]
residuals = lmh137["FitResiduals"];
rmse = Sqrt[Mean[residuals^2]];
{lmh137[{"ParameterTable", "RSquared", "AdjustedRSquared"}], rmse}
```

Out[∘]= FittedModel[$-2.6018 + 1.05637\, x$]

Out[∘]=

	Estimate	Standard Error	t-Statistic	P-Value
1	−2.6018	1.29429	−2.01022	0.0721482
x	1.05637	0.0260772	40.5094	2.01116×10^{-12}

, 0.993943, 0.993337}, 3.13442}

In[∘]:=
```
Max[a053⟦All, 2⟧]
Max[a137⟦All, 2⟧]
Max[h053⟦All, 2⟧]
Max[h137⟦All, 2⟧]
```

Out[∘]= 2.12538

Out[∘]= 6.71754

Out[∘]= 64.2413

Out[∘]= 107.558

In[∘]:=
```
(*concentration vs peak area/height plots;
red for 0.53 THz peak, blue for 1.37 THz peak*)
plota053 = ListPlot[a053, PlotStyle → ■, PlotRange → {0, 7}];
linea053 = Plot[lma053[x], {x, 0, 100},
   PlotStyle → ■, LabelStyle → Directive[FontSize → 16, ■]];

plota137 = ListPlot[a137, PlotStyle → ■, PlotRange → {0, 7}];
linea137 = Plot[lma137[x], {x, 0, 100},
   PlotStyle → ■, LabelStyle → Directive[FontSize → 16, ■]];

ploth053 = ListPlot[h053, PlotStyle → ■, PlotRange → {0, 110}];
lineh053 = Plot[lmh053[x], {x, 0, 100},
   PlotStyle → ■, LabelStyle → Directive[FontSize → 16, ■]];

ploth137 = ListPlot[h137, PlotStyle → ■, PlotRange → {0, 110}];
lineh137 = Plot[lmh137[x], {x, 0, 100},
   PlotStyle → ■, LabelStyle → Directive[FontSize → 16, ■]];

Show[plota053, linea053, plota137,
 linea137, Frame → {{True, False}, {True, False}},
 FrameLabel → {{"Peak area (arbitrary unit)", None},
   {"Lactose concentration in % (w/w)", None}},
 FrameTicks → All, LabelStyle → Directive[FontSize → 16]]
Show[ploth053, lineh053, ploth137, lineh137,
 Frame → {{True, False}, {True, False}},
 FrameLabel → {{"Peak height (arbitrary unit)", None},
   {"Lactose concentration in % (w/w)", None}},
 FrameTicks → All, LabelStyle → Directive[FontSize → 16]]
```

Out[ ]=

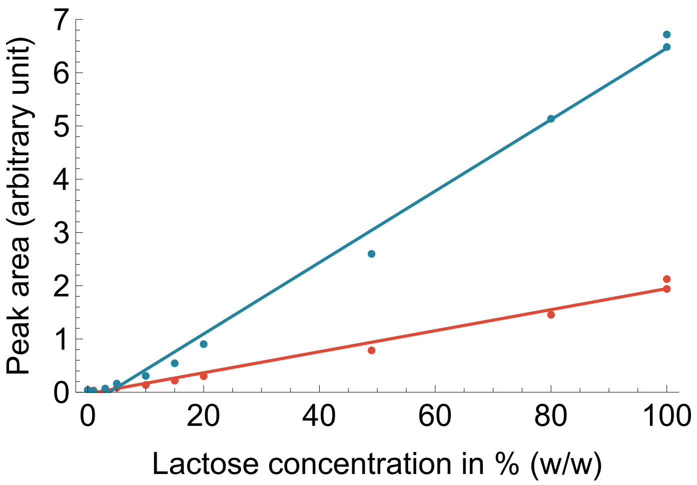

Out[ ]=

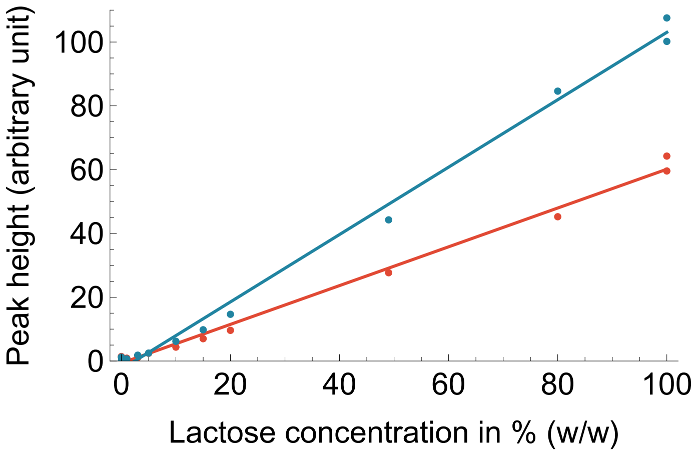

r^2, RMSE and predicted concentration

In[ ]:=

```
peak = "area-0.53";
(*choose predictor here
  ("area" or "height" of "0.53" THz or "1.37" THz peak): "area-0.53",
"area-1.37","height-0.53" or "height-1.37"*)
dataset = {"set1_ambient_1", "set1_N2_1", "set1_N2_2", "set2_ambient_1",
   "set2_N2_1", "set2_N2_2", "set2_N2_3", "set2_N2_4", "set2_N2_5"};
col = <|{"area-0.53" → 3, "area-1.37" → 4, "height-0.53" → 5, "height-1.37" → 6}|>;
(*column number of each predictor*)
r2tab = {};
adjr2tab = {};
rmsetab = {};
s1tab = {};
s2tab = {};
s3tab = {};
overrange = {};
For[i = 1, i ≤ Length[dataset], i++,
 path = NotebookDirectory[] <> "../data/" <> dataset⟦i⟧ <> "/";
 method = {"poly", "als", "arpls", "drpls"};
 (*baseline correction methods; please refer to analysis.ipynb*)
```

```
result = {}; s1 = {}; s2 = {}; s3 = {};
For[j = 1, j ≤ Length[method], j++,
  data0 = Import[
    path <> "peak_area_and_height/absorption_coefficient_spectra/peakvalues-" <>
     method⟦j⟧ <> ".csv"];
  (*import peak are and peak height values*)
  cdata = data0⟦1 ;; 12⟧;
  (*calibration data of pure lactose standards*)
  (*filter out overrange values, only values ≥ 0 are included,
  values in range (-0.1, 0) are treated as 0*)
  peakvalue = {};
  For[k = 1, k ≤ Length[cdata], k++, y = cdata⟦k⟧⟦col[peak]⟧;
   If[y ≥ 0, AppendTo[peakvalue, {cdata⟦k⟧⟦2⟧, y}],
    If[-0.1 < y < 0, AppendTo[peakvalue, {cdata⟦k⟧⟦2⟧, 0}],
     AppendTo[overrange, {dataset⟦i⟧, method⟦j⟧, cdata⟦k⟧}]]]];
  lm = LinearModelFit[peakvalue, x, x];
  (*linear regression model*)
  trendline = Normal[lm]; (*equation of trendline*)
  residuals = lm["FitResiduals"];
  r2 = lm["RSquared"];
  adjr2 = lm["AdjustedRSquared"];
  AppendTo[result, {trendline, NumberForm[r2, {5, 4}],
    NumberForm[adjr2, {5, 4}], NumberForm[rmse = √(Mean[residuals^2]), {6, 5}]}];
  sdata = data0⟦{13, 14, 15}⟧;
  prediction = {};
  (*data of milk powder samples*)
  For[l = 1, l ≤ Length[sdata], l++, peakval = sdata⟦l⟧⟦col[peak]⟧;
   sol = Solve[trendline == peakval, x]⟦1⟧;
   (*solve for concentration from peak area or height*)
   AppendTo[prediction, If[peakval > -0.1, NumberForm[conc = x /. sol, {3, 1}],
     "nd"](*overrange peak values are reported as "nd" for not detected*)
   ]];
  AppendTo[s1, prediction⟦1⟧];
  AppendTo[s2, prediction⟦2⟧];
  AppendTo[s3, prediction⟦3⟧];
 ];
 AppendTo[r2tab, result⟦All, 2⟧];
 AppendTo[adjr2tab, result⟦All, 3⟧];
 AppendTo[rmsetab, result⟦All, 4⟧];
 AppendTo[s1tab, s1];
 AppendTo[s2tab, s2];
 AppendTo[s3tab, s3];
]
overrange(*excluded overrange values*)
TableForm[r2tab, TableHeadings → {dataset, method}](*table of r^2 values*)
```

```
TableForm[rmsetab, TableHeadings → {dataset, method}] (*table of RMSE values*)
TableForm[s1tab, TableHeadings → {dataset, method}]
(*table of predicted concentration of sample 1*)
TableForm[s2tab, TableHeadings → {dataset, method}]
(*table of predicted concentration of sample 2*)
TableForm[s3tab, TableHeadings → {dataset, method}]
(*table of predicted concentration of sample 3*)
```

Out[•]= { }

Out[•]//TableForm=

	poly	als	arpls	drpls
set1_ambient_1	0.9866	0.9880	0.9878	0.9877
set1_N2_1	0.9931	0.9971	0.9893	0.9922
set1_N2_2	0.9923	0.9933	0.9925	0.9916
set2_ambient_1	0.9923	0.9874	0.9850	0.9847
set2_N2_1	0.9941	0.9962	0.9901	0.9923
set2_N2_2	0.9942	0.9906	0.9928	0.9916
set2_N2_3	0.9924	0.9925	0.9845	0.9854
set2_N2_4	0.9881	0.9986	0.9875	0.9878
set2_N2_5	0.9865	0.9948	0.9876	0.9876

Out[•]//TableForm=

	poly	als	arpls	drpls
set1_ambient_1	0.06935	0.08339	0.07898	0.07878
set1_N2_1	0.04818	0.04995	0.07662	0.06722
set1_N2_2	0.05077	0.07767	0.06517	0.07035
set2_ambient_1	0.05348	0.09438	0.08873	0.08977
set2_N2_1	0.04424	0.05634	0.07384	0.06660
set2_N2_2	0.04435	0.09167	0.06387	0.07025
set2_N2_3	0.04652	0.07606	0.09018	0.08819
set2_N2_4	0.06228	0.03440	0.08298	0.08337
set2_N2_5	0.06693	0.06752	0.08233	0.08397

Out[•]//TableForm=

	poly	als	arpls	drpls
set1_ambient_1	50.9	47.6	48.9	44.9
set1_N2_1	53.5	56.8	55.1	58.5
set1_N2_2	50.2	52.7	56.5	62.3
set2_ambient_1	51.7	48.0	45.0	45.4
set2_N2_1	58.3	58.9	56.0	58.7
set2_N2_2	58.2	52.8	59.0	71.4
set2_N2_3	62.2	63.3	61.4	64.9
set2_N2_4	54.8	55.8	60.7	67.5
set2_N2_5	57.0	57.4	57.5	66.7

Out[ ]//TableForm=

	poly	als	arpls	drpls
set1_ambient_1	20.0	13.3	16.2	16.6
set1_N2_1	20.5	20.2	15.6	17.0
set1_N2_2	18.2	24.4	16.0	17.4
set2_ambient_1	18.8	15.6	16.5	17.8
set2_N2_1	19.5	24.2	15.9	17.5
set2_N2_2	18.4	28.5	40.2	37.6
set2_N2_3	18.3	25.5	17.2	18.1
set2_N2_4	18.9	28.0	26.3	33.2
set2_N2_5	21.9	22.8	16.6	18.0

Out[ ]//TableForm=

	poly	als	arpls	drpls
set1_ambient_1	7.5	nd	nd	nd
set1_N2_1	7.5	4.8	7.0	nd
set1_N2_2	5.1	0.8	nd	6.3
set2_ambient_1	-1.8	nd	nd	7.9
set2_N2_1	-4.3	3.1	nd	6.2
set2_N2_2	-5.7	3.2	-4.0	6.5
set2_N2_3	5.9	3.5	1.8	5.5
set2_N2_4	4.9	4.6	6.2	8.2
set2_N2_5	-4.2	4.8	6.8	nd
