# Supplementary material for: Terahertz Spectroscopic Analysis of Lactose in Infant Formula: Implications for Detection and Quantification
Source: Molecules. 2022 Aug 8;27(15):5040. doi: 10.3390/molecules27155040 (PMC9370465; doi:10.3390/molecules27155040)

DOI: 10.3390/molecules27155040

Transmission & absorption spectra

In[◦]:=

```
dataset = "set2_N2_5";(*choose dataset folder here*)
col = 3;(*choose column number: 2 for transmission, 3 for absorption*)
path = NotebookDirectory[] <> "../data/" <> dataset <> "/frequency_domain/";
(*path for importing files containing frequency domain data*)
colour = Reverse[{■, ■, ■, ■, ■, ■, ■, ■, ■, ■, ■, ■, ■, ■}];
data0 = Import[path <> "Ref.csv"]⟦All, {1, col}⟧;
(*import transmittance/absorbance values for each sample*)
Ref = ListPlot[Table[{data0⟦i⟧⟦1⟧, data⟦i⟧⟦2⟧ - data0⟦i⟧⟦2⟧}, {i, Length[data]}],
   Joined → True, PlotStyle → colour⟦1⟧];
data = Import[path <> "L000_1.csv"]⟦All, {1, col}⟧;
L0a = ListPlot[data, Joined → True, PlotStyle → colour⟦2⟧];
data = Import[path <> "L000_2.csv"]⟦All, {1, col}⟧;
L0b = ListPlot[data, Joined → True, PlotStyle → colour⟦2⟧];
data = Import[path <> "L001.csv"]⟦All, {1, col}⟧;
L1 = ListPlot[data, Joined → True, PlotStyle → colour⟦3⟧];
data = Import[path <> "L003.csv"]⟦All, {1, col}⟧;
L3 = ListPlot[data, Joined → True, PlotStyle → colour⟦4⟧];
data = Import[path <> "L005.csv"]⟦All, {1, col}⟧;
L5 = ListPlot[data, Joined → True, PlotStyle → colour⟦5⟧];
data = Import[path <> "L010.csv"]⟦All, {1, col}⟧;
L10 = ListPlot[data, Joined → True, PlotStyle → colour⟦6⟧];
data = Import[path <> "L015.csv"]⟦All, {1, col}⟧;
L15 = ListPlot[data, Joined → True, PlotStyle → colour⟦7⟧];
data = Import[path <> "L020.csv"]⟦All, {1, col}⟧;
L20 = ListPlot[data, Joined → True, PlotStyle → colour⟦8⟧];
data = Import[path <> "L049.csv"]⟦All, {1, col}⟧;
L49 = ListPlot[data, Joined → True, PlotStyle → colour⟦9⟧];
data = Import[path <> "L080.csv"]⟦All, {1, col}⟧;
L80 = ListPlot[data, Joined → True, PlotStyle → colour⟦10⟧];
data = Import[path <> "L100_1.csv"]⟦All, {1, col}⟧;
L100a = ListPlot[data, Joined → True, PlotStyle → colour⟦11⟧];
data = Import[path <> "L100_2.csv"]⟦All, {1, col}⟧;
L100b = ListPlot[data, Joined → True, PlotStyle → colour⟦11⟧];
data = Import[path <> "S001.csv"]⟦All, {1, col}⟧;
S1 = ListPlot[data, Joined → True, PlotStyle → colour⟦12⟧];
data = Import[path <> "S002.csv"]⟦All, {1, col}⟧;
S2 = ListPlot[data, Joined → True, PlotStyle → colour⟦13⟧];
data = Import[path <> "S003.csv"]⟦All, {1, col}⟧;
S3 = ListPlot[data, Joined → True, PlotStyle → colour⟦14⟧];
```

In[•]:=
```
(*show selected samples on the same plot - all pure standard samples*)
Show[Ref, L0b, L1, L3, L5, L10, L15, L20, L49, L80, L100b,
 PlotRange → {{0.2, 2}, {0, 1.5}}, Frame → {{True, False}, {True, False}},
 GridLines → {{{0.53, Directive[Dashed, Thickness[0.005]]},
    {1.20, Directive[Dashed, Thickness[0.005]]},
    {1.37, Directive[Dashed, Thickness[0.005]]},
    {1.82, Directive[Dashed, Thickness[0.005]]}}, None},
 FrameLabel → {{"absorbance (arbitrary unit)", None}, {"frequency (THz)", None}},
 FrameTicks → All, LabelStyle → Directive[FontSize → 16]]
```

Out[•]=

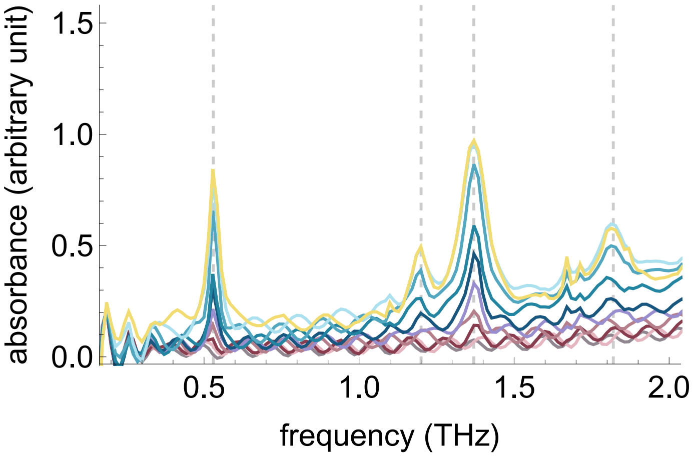

In[•]:=
```
(*show selected samples on the same plot -
 pure standard and all real samples*)
Show[L0b, L15, S1, S2, S3, PlotRange → {{0.2, 2}, {-0.5, 1.5}},
 Frame → {{True, False}, {True, False}},
 FrameLabel → {{"absorbance (arbitrary unit)", None}, {"frequency (THz)", None}},
 FrameTicks → All, GridLines → {{{0.53, Directive[Dashed, Thickness[0.005]]},
    {1.37, Directive[Dashed, Thickness[0.005]]}}, None},
 LabelStyle → Directive[FontSize → 14]]
```

Out[•]=

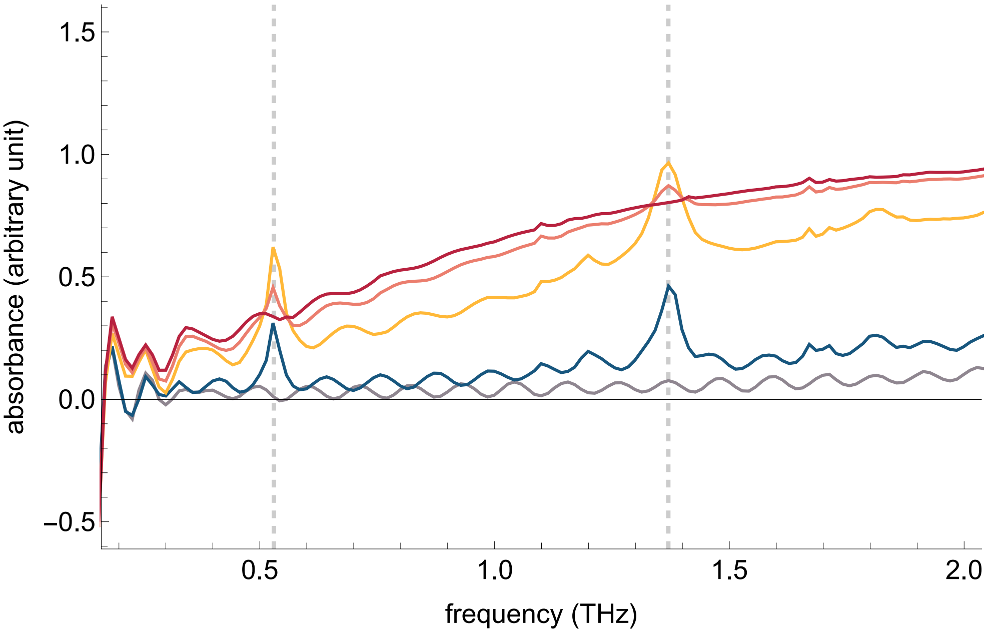

Refractive index spectra

```
path = "../data/set2_N2_5/frequency_domain/";
ri = Import[NotebookDirectory[] <> path <> "refractive-index.csv"];
Length[ri]
colour = Reverse[{■, ■, ■, ■, ■, ■, ■, ■, ■, ■, ■, ■, ■, ■}];
list = {3, 4, 5, 6, 7, 8, 9, 10, 11, 17};
(*choose columns of data - only pure standards are chosen here*)
ListPlot[Table[ri⟦All, {1, i}⟧, {i, list}],
 PlotStyle → colour⟦2 ;; 11⟧, Joined → True,
 PlotRange → {{0.3, 2}, {1.35, 1.9}}, Frame → {{True, False}, {True, False}},
 FrameLabel → {{"refractive index (n)", None}, {"frequency (THz)", None}},
 FrameTicks → All, GridLines → {{{0.53, Directive[Dashed, Thickness[0.006]]},
    {1.20, Directive[Dashed, Thickness[0.006]]}, {1.37, Directive[Dashed,
      Thickness[0.006]]}, {1.82, Directive[Dashed, Thickness[0.006]]}},
   None}, LabelStyle → Directive[FontSize → 14]]
```

Out[∘]= 702

Out[∘]=

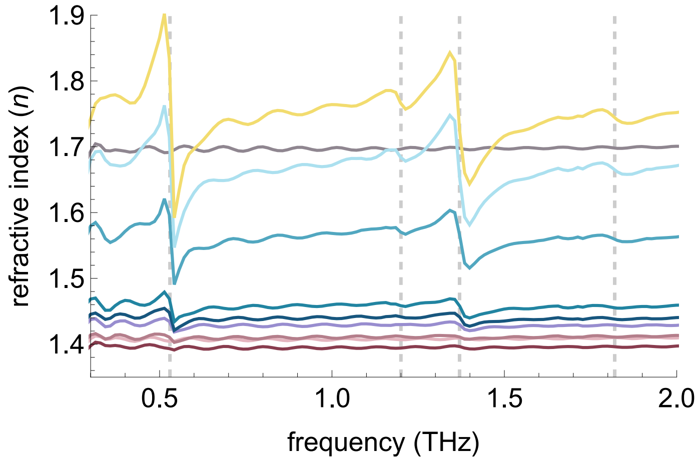

Absorption coefficient spectra

```
path = "../data/set2_N2_5/frequency_domain/";
ac = Import[NotebookDirectory[] <> path <> "absorption-coefficient.csv"];
Length[ac]
colour = Reverse[{■, ■, ■, ■, ■, ■, ■, ■, ■, ■, ■, ■, ■, ■}];
list = {3, 4, 5, 6, 7, 8, 9, 10, 11, 17};
(*choose columns of data - only pure standards are chosen here*)
ListPlot[Table[ac[[All, {1, i}]], {i, list}],
 PlotStyle → colour[[2 ;; 11]], Joined → True, PlotRange → {{0.3, 2}, {0, 120}},
 Frame → {{True, False}, {True, False}}, FrameLabel →
  {{"absorption coefficient (α) in cm^-1", None}, {"frequency (THz)", None}},
 FrameTicks → All, GridLines → {{{0.53, Directive[Dashed, Thickness[0.006]]},
    {1.20, Directive[Dashed, Thickness[0.006]]}, {1.37, Directive[Dashed,
      Thickness[0.006]]}, {1.82, Directive[Dashed, Thickness[0.006]]}},
   None}, LabelStyle → Directive[FontSize → 14]]
```

Out[∘]= 702

Out[∘]=

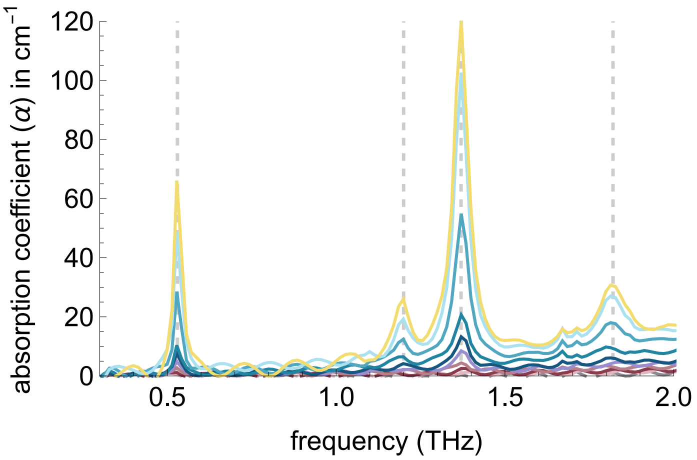

```
list = {3, 8, 12, 15, 16};(*choose columns of data - real samples,
0% and 15% (w/w) pure standards are chosen here*)
ListPlot[Table[ac⟦All, {1, i}⟧, {i, list}], PlotStyle → colour⟦{2, 7, 12, 13, 14}⟧,
 Joined → True, PlotRange → {{0.3, 2}, {0, 120}},
 Frame → {{True, False}, {True, False}}, FrameLabel →
  {{"absorption coefficient (α) in cm^-1", None}, {"frequency (THz)", None}},
 FrameTicks → All, GridLines → {{{0.53, Directive[Dashed, Thickness[0.006]]},
    {1.20, Directive[Dashed, Thickness[0.006]]},
    {1.37, Directive[Dashed, Thickness[0.006]]},
    {1.82, Directive[Dashed, Thickness[0.006]]}}, None},
 LabelStyle → Directive[FontSize → 14]]
```

Out[ ]=

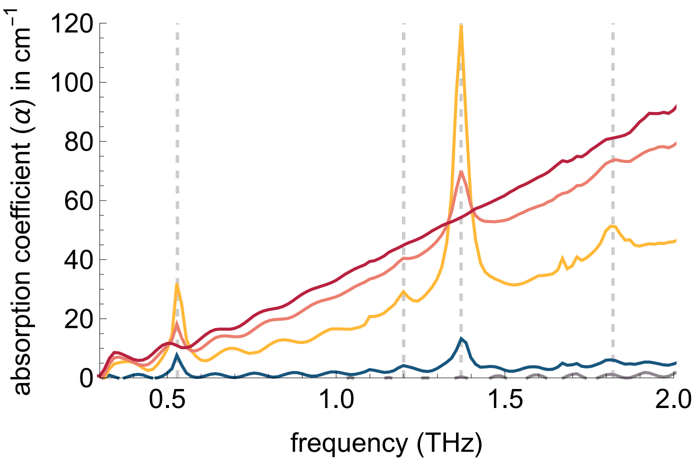

Supplement: Supplementary file 1 [file molecules-27-05040-s001.zip › codes/spectra-visualization.pdf]
