# Supplementary material for: Terahertz Spectroscopic Analysis of Lactose in Infant Formula: Implications for Detection and Quantification
Source: Molecules. 2022 Aug 8;27(15):5040. doi: 10.3390/molecules27155040 (PMC9370465; doi:10.3390/molecules27155040)
Supplement: Supplementary file 1 [file molecules-27-05040-s001.zip › data/absorption_coefficient_spectra_prediction.pdf]

DOI: 10.3390/molecules27155040

***r^2* values**

Predictor: **0.53 THz peak area**

Dataset \ Method	poly	als	arpls	drpls
220311	0.9866	0.9880	0.9878	0.9877
220311_N	0.9931	0.9971	0.9893	0.9922
220311_N2	0.9923	0.9933	0.9925	0.9916
220315	0.9923	0.9874	0.9850	0.9847
220315_N1	0.9941	0.9962	0.9901	0.9923
220315_N2	0.9942	0.9906	0.9928	0.9916
220315_N3	0.9924	0.9925	0.9845	0.9854
220315_N4	0.9881	0.9986	0.9875	0.9878
220315_N5	0.9865	0.9948	0.9876	0.9876

Predictor: **0.53 THz peak height**

Dataset \ Method	poly	als	arpls	drpls
220311	0.0641	0.0641	0.9943	0.9938
220311_N	0.9950	0.0641	0.9944	0.9945
220311_N2	0.9954	0.0641	0.9956	0.9954
220315	0.9966	0.0526	0.9939	0.9934
220315_N1	0.9956	0.0641	0.9948	0.9948
220315_N2	0.9959	0.0641	0.9957	0.9955
220315_N3	0.0526	0.0641	0.0526	0.0526
220315_N4	0.0526	0.0641	0.0526	0.0526
220315_N5	0.9927	0.0641	0.9935	0.9934

Predictor: **1.37 THz peak area**

Dataset \ Method	poly	als	arpls	drpls
220311	0.9856	0.9809	0.9852	0.9847
220311_N	0.9919	0.9893	0.9892	0.9886
220311_N2	0.9948	0.9928	0.9932	0.9923
220315	0.9877	0.9750	0.9774	0.9778
220315_N1	0.9904	0.9904	0.9895	0.9881
220315_N2	0.9945	0.9933	0.9933	0.9923
220315_N3	0.9817	0.9826	0.9813	0.9793
220315_N4	0.9868	0.9864	0.9853	0.9837
220315_N5	0.9949	0.9931	0.9939	0.9929

Predictor: **1.37 THz peak height**

Dataset \ Method	poly	als	arpls	drpls
220311	0.9879	0.0634	0.9894	0.9893
220311_N	0.0601	0.0641	0.0004	0.9909
220311_N2	0.9945	0.0641	0.9941	0.9943
220315	0.9909	0.0641	0.9893	0.9903
220315_N1	0.0601	0.0693	0.0601	0.9908
220315_N2	0.9943	0.0641	0.9916	0.0775
220315_N3	0.0641	0.0643	0.9847	0.9839
220315_N4	0.9880	0.0641	0.9877	0.9875
220315_N5	0.0601	0.0641	0.0775	0.9940

DOI: 10.3390/molecules27155040

RMSE values

Predictor: **0.53 THz peak area**

Method / Dataset	poly	als	arpls	drpls
220311	0.0694	0.0834	0.0790	0.0788
220311_N	0.0482	0.0500	0.0766	0.0672
220311_N2	0.0508	0.0777	0.0652	0.0704
220315	0.0535	0.0944	0.0887	0.0898
220315_N1	0.0442	0.0563	0.0738	0.0666
220315_N2	0.0444	0.0917	0.0639	0.0703
220315_N3	0.0465	0.0761	0.0902	0.0882
220315_N4	0.0623	0.0344	0.0830	0.0834
220315_N5	0.0669	0.0675	0.0823	0.0840

Predictor: **0.53 THz peak height**

Method / Dataset	poly	als	arpls	drpls
220311	2.59E+35	5.57E+39	1.7495	1.7993
220311_N	1.5387	5.91E+27	1.7263	1.7207
220311_N2	1.4828	6.95E+24	1.5442	1.5823
220315	1.3139	5.51E+26	1.8451	1.8724
220315_N1	1.4583	2.39E+27	1.6791	1.6810
220315_N2	1.4189	4.52E+27	1.5326	1.5688
220315_N3	2.28E+18	1.99E+29	7.63E+14	4.01E+15
220315_N4	2.80E+20	5.91E+30	1.15E+19	8.39E+21
220315_N5	1.8678	1.10E+30	1.8682	1.8840

Predictor: **1.37 THz peak area**

Method / Dataset	poly	als	arpls	drpls
220311	0.2251	0.3654	0.2995	0.3002
220311_N	0.1688	0.2967	0.2760	0.2798
220311_N2	0.1347	0.2427	0.2179	0.2289
220315	0.2088	0.3690	0.3732	0.3638
220315_N1	0.1828	0.2816	0.2729	0.2865
220315_N2	0.1385	0.2351	0.2174	0.2292
220315_N3	0.2625	0.3911	0.3762	0.3920
220315_N4	0.2168	0.3412	0.3246	0.3388
220315_N5	0.1282	0.2343	0.2006	0.2141

Predictor: **1.37 THz peak height**

Method / Dataset	poly	als	arpls	drpls
220311	4.0619	2.29E+04	4.2074	4.2585
220311_N	9.00E+06	1.11E+32	1.78E+02	3.9957
220311_N2	2.8071	1.54E+25	3.1698	3.1084
220315	3.7216	1.77E+18	4.2598	4.1022
220315_N1	3.54E+43	7.15E+26	2.44E+43	4.0695
220315_N2	2.8794	1.37E+20	3.7740	5.33E+22
220315_N3	2.37E+21	5.08E+16	5.3428	5.5538
220315_N4	4.3399	8.26E+31	4.6836	4.7988
220315_N5	2.36E+15	6.34E+34	2.01E+11	3.1623

DOI: 10.3390/molecules27155040

Concentration of α-lactose monohydrate

Predicted concentration by absorption coefficient spectra

*nd = not detected (absorption coefficient < -0.1)

Predictor: **0.53 THz peak area**

Sample: 1

Method / Dataset	poly	als	arpls	drpls
set1_ambient_1	50.9	47.6	48.9	44.9
set1_N2_1	53.5	56.8	55.1	58.5
set1_N2_2	50.2	52.7	56.5	62.3
**Mean (set1_N2)**	51.9	54.8	55.8	60.4
**SD (set1_N2)**	2.3	2.9	1.0	2.7
**CV (set1_N2)**	5%	5%	2%	4%
set2_ambient_1	51.7	48.0	45.0	45.4
set2_N2_1	58.3	58.9	56.0	58.7
set2_N2_2	58.2	52.8	59.0	71.4
set2_N2_3	62.2	63.3	61.4	64.9
set2_N2_4	54.8	55.8	60.7	67.5
set2_N2_5	57.0	57.4	57.5	66.7
**Mean (set2_N2)**	58.1	57.6	58.9	65.8
**SD (set2_N2)**	2.7	3.9	2.2	4.6
**CV (set2_N2)**	5%	7%	4%	7%

Sample: 2

Method / Dataset	poly	als	arpls	drpls
set1_ambient_1	20.0	13.3	16.2	16.6
set1_N2_1	20.5	20.2	15.6	17.0
set1_N2_2	18.2	24.4	16.0	17.4
**Mean (set1_N2)**	19.4	22.3	15.8	17.2
**SD (set1_N2)**	1.6	3.0	0.3	0.3
**CV (set1_N2)**	8%	13%	2%	2%
set2_ambient_1	18.8	15.6	16.5	17.8
set2_N2_1	19.5	24.2	15.9	17.5
set2_N2_2	18.4	28.5	40.2	37.6
set2_N2_3	18.3	25.5	17.2	18.1
set2_N2_4	18.9	28.0	26.3	33.2
set2_N2_5	21.9	22.8	16.6	18.0
**Mean (set2_N2)**	19.4	25.8	23.2	24.9
**SD (set2_N2)**	1.5	2.4	10.4	9.7
**CV (set2_N2)**	8%	9%	45%	39%

Sample: 3

Method / Dataset	poly	als	arpls	drpls
set1_ambient_1	7.5	nd	nd	nd
set1_N2_1	7.5	4.8	7.0	nd
set1_N2_2	5.1	0.8	nd	6.3
**Mean (set1_N2)**	6.3	2.8	7.0	6.3
**SD (set1_N2)**	1.7	2.8	#DIV/0!	#DIV/0!
**CV (set1_N2)**	27%	101%	#DIV/0!	#DIV/0!
set2_ambient_1	-1.8	nd	nd	7.9
set2_N2_1	-4.3	3.1	nd	6.2
set2_N2_2	-5.7	3.2	-4.0	6.5
set2_N2_3	5.9	3.5	1.8	5.5
set2_N2_4	4.9	4.6	6.2	8.2
set2_N2_5	-4.2	4.8	6.8	nd
**Mean (set2_N2)**	-0.7	3.8	2.7	6.6
**SD (set2_N2)**	5.6	0.8	5.0	1.1
**CV (set2_N2)**	-823%	21%	185%	17%

Predictor: **1.37 THz peak area**

Sample: 1

Method / Dataset	poly	als	arpls	drpls
set1_ambient_1	73.3	77.1	68.6	68.7
set1_N2_1	75.4	69.1	72.2	71.9
set1_N2_2	73.0	65.1	70.4	70.4
**Mean (set1_N2)**	74.2	67.1	71.3	71.2
**SD (set1_N2)**	1.7	2.8	1.3	1.1
**CV (set1_N2)**	2%	4%	2%	1%
set2_ambient_1	74.0	78.0	69.0	70.5
set2_N2_1	76.8	69.6	72.3	72.0
set2_N2_2	75.5	66.2	71.4	71.2
set2_N2_3	74.5	68.2	70.2	70.2
set2_N2_4	75.2	68.0	71.8	71.9
set2_N2_5	78.7	70.5	74.6	74.6
**Mean (set2_N2)**	76.1	68.5	72.1	72.0
**SD (set2_N2)**	1.7	1.6	1.6	1.6
**CV (set2_N2)**	2%	2%	2%	2%

Sample: 2

Method / Dataset	poly	als	arpls	drpls
set1_ambient_1	24.0	54.9	18.3	16.9
set1_N2_1	23.2	28.0	21.2	21.0
set1_N2_2	22.9	23.0	22.1	21.4
**Mean (set1_N2)**	23.1	25.5	21.7	21.2
**SD (set1_N2)**	0.2	3.5	0.6	0.3
**CV (set1_N2)**	1%	14%	3%	1%
set2_ambient_1	23.1	48.8	19.5	18.2
set2_N2_1	23.3	24.2	21.9	21.9
set2_N2_2	23.4	21.3	22.6	22.4
set2_N2_3	21.0	24.8	21.4	21.0
set2_N2_4	22.7	21.7	22.5	22.2
set2_N2_5	24.8	27.1	22.6	22.0
**Mean (set2_N2)**	23.0	23.8	22.2	21.9
**SD (set2_N2)**	1.4	2.4	0.5	0.5
**CV (set2_N2)**	6%	10%	2%	2%

Sample: 3

Method / Dataset	poly	als	arpls	drpls
set1_ambient_1	5.9	41.1	3.4	nd
set1_N2_1	3.5	9.6	1.8	nd
set1_N2_2	1.7	16.9	2.4	nd
**Mean (set1_N2)**	2.6	13.3	2.1	#DIV/0!
**SD (set1_N2)**	1.3	5.2	0.4	#DIV/0!
**CV (set1_N2)**	49%	39%	20%	#DIV/0!
set2_ambient_1	2.7	31.5	2.2	nd
set2_N2_1	1.3	11.4	2.0	nd
set2_N2_2	1.1	10.5	2.6	nd
set2_N2_3	2.7	9.5	2.4	nd
set2_N2_4	2.5	8.7	2.3	nd
set2_N2_5	1.7	10.1	2.9	nd
**Mean (set2_N2)**	1.9	10.0	2.4	#DIV/0!
**SD (set2_N2)**	0.7	1.0	0.3	#DIV/0!
**CV (set2_N2)**	38%	10%	14%	#DIV/0!

Predictor: **0.53 THz peak height**

Sample: 1

Method / Dataset	poly	als	arpls	drpls
set1_ambient_1	77.2	77.2	44.9	43.9
set1_N2_1	45.5	77.2	45.0	45.0
set1_N2_2	44.5	77.2	44.6	44.5
**Mean (set1_N2)**	45.0	77.2	44.8	44.8
**SD (set1_N2)**	0.7	0.0	0.3	0.4
**CV (set1_N2)**	2%	0%	1%	1%
set2_ambient_1	52.3	47.7	47.3	45.8
set2_N2_1	46.4	81.9	43.6	43.4
set2_N2_2	46.1	77.2	44.8	44.8
set2_N2_3	45.7	77.2	44.5	44.1
set2_N2_4	81.9	77.2	81.9	81.9
set2_N2_5	81.9	77.2	81.9	81.9
**Mean (set2_N2)**	45.4	77.2	44.5	44.5
**SD (set2_N2)**	19.6	2.1	20.6	20.7
**CV (set2_N2)**	43%	3%	46%	47%

Sample: 2

Method / Dataset	poly	als	arpls	drpls
set1_ambient_1	77.2	77.2	15.5	14.8
set1_N2_1	16.3	77.2	15.3	15.2
set1_N2_2	15.9	77.2	15.1	14.8
**Mean (set1_N2)**	16.1	77.2	15.2	15.0
**SD (set1_N2)**	0.3	0.0	0.1	0.3
**CV (set1_N2)**	2%	0%	1%	2%
set2_ambient_1	19.1	81.9	15.9	15.3
set2_N2_1	16.2	77.2	15.3	15.2
set2_N2_2	15.9	77.2	17.9	17.5
set2_N2_3	81.9	77.2	81.9	81.9
set2_N2_4	81.9	77.2	81.9	81.9
set2_N2_5	16.4	77.2	15.3	15.0
**Mean (set2_N2)**	42.5	77.2	42.5	42.3
**SD (set2_N2)**	36.0	0.0	36.0	36.2
**CV (set2_N2)**	85%	0%	85%	85%

Sample: 3

Method / Dataset	poly	als	arpls	drpls
set1_ambient_1	77.2	nd	nd	nd
set1_N2_1	5.0	77.2	5.4	nd
set1_N2_2	4.1	77.2	nd	5.4
**Mean (set1_N2)**	4.6	77.2	5.4	5.4
**SD (set1_N2)**	0.6	0.0	#DIV/0!	#DIV/0!
**CV (set1_N2)**	14%	0%	#DIV/0!	#DIV/0!
set2_ambient_1	nd	nd	nd	6.1
set2_N2_1	nd	77.2	nd	5.3
set2_N2_2	nd	77.2	nd	5.4
set2_N2_3	81.9	77.2	81.9	81.9
set2_N2_4	81.9	77.2	81.9	81.9
set2_N2_5	nd	77.2	5.3	nd
**Mean (set2_N2)**	81.9	77.2	56.4	43.6
**SD (set2_N2)**	0.0	0.0	44.2	44.2
**CV (set2_N2)**	0%	0%	78%	101%

Predictor: **1.37 THz peak height**

Sample: 1

Method / Dataset	poly	als	arpls	drpls
set1_ambient_1	79.3	77.2	78.0	78.0
set1_N2_1	78.6	77.2	80.5	78.4
set1_N2_2	78.8	77.2	77.8	77.8
**Mean (set1_N2)**	78.7	77.2	79.2	78.1
**SD (set1_N2)**	0.1	0.0	1.9	0.4
**CV (set1_N2)**	0%	0%	2%	1%
set2_ambient_1	79.8	77.2	78.6	78.9
set2_N2_1	78.6	77.2	78.6	78.4
set2_N2_2	79.8	77.2	78.2	78.4
set2_N2_3	77.2	77.2	76.2	76.2
set2_N2_4	79.7	77.2	78.3	78.3
set2_N2_5	78.6	77.2	78.4	81.1
**Mean (set2_N2)**	78.8	77.2	77.9	78.5
**SD (set2_N2)**	1.1	0.0	1.0	1.7
**CV (set2_N2)**	1%	0%	1%	2%

Sample: 2

Method / Dataset	poly	als	arpls	drpls
set1_ambient_1	22.3	77.5	21.6	21.4
set1_N2_1	78.6	77.2	714.0	21.0
set1_N2_2	21.1	77.2	21.0	20.9
**Mean (set1_N2)**	49.9	77.2	367.5	21.0
**SD (set1_N2)**	40.7	0.0	490.0	0.1
**CV (set1_N2)**	82%	0%	133%	0%
set2_ambient_1	23.9	77.2	21.3	21.4
set2_N2_1	78.6	77.2	78.6	21.3
set2_N2_2	21.4	77.2	20.8	78.4
set2_N2_3	77.2	77.2	19.8	20.0
set2_N2_4	21.2	77.2	20.8	21.1
set2_N2_5	78.6	77.2	78.4	21.8
**Mean (set2_N2)**	55.4	77.2	43.7	32.5
**SD (set2_N2)**	31.1	0.0	31.8	25.7
**CV (set2_N2)**	56%	0%	73%	79%

Sample: 3

Method / Dataset	poly	als	arpls	drpls
set1_ambient_1	2.9	7.39E+01	nd	nd
set1_N2_1	78.5	-2.20E+18	nd	nd
set1_N2_2	nd	77.2	nd	nd
**Mean (set1_N2)**	78.5	-1.10E+18	#DIV/0!	#DIV/0!
**SD (set1_N2)**	#DIV/0!	1.56E+18	#DIV/0!	#DIV/0!
**CV (set1_N2)**	#DIV/0!	-141%	#DIV/0!	#DIV/0!
set2_ambient_1	nd	7.72E+01	nd	nd
set2_N2_1	nd	-2.20E+08	nd	nd
set2_N2_2	nd	77.2	nd	nd
set2_N2_3	76.5	-1.30E+07	nd	nd
set2_N2_4	nd	77.2	nd	nd
set2_N2_5	nd	77.2	nd	nd
**Mean (set2_N2)**	76.5	-4.66E+07	#DIV/0!	#DIV/0!
**SD (set2_N2)**	#DIV/0!	9.71E+07	#DIV/0!	#DIV/0!
**CV (set2_N2)**	#DIV/0!	-208%	#DIV/0!	#DIV/0!

DOI: 10.3390/molecules27155040

Concentration of lactose anhydrous (≈95% of α-lactose monohydrate)

HPLC quantification (by external accredited laboratories)

		Sample 1	Sample 2
AOAC (2019) 980.13	1	**62.70**	**60.46**
	2	**61.24**	**62.52**
		Ref. No. SFC 1020/2565	Ref No. SFC 0823/2565
		27-Jun-22	11-Apr-22
AOAC (2019) 982.14	3	**not done**	**56.92**
			Ref No. 6501.1911/651949
			1-Apr-22
Mean		**61.97**	**59.97**

Predicted concentration by absorption coefficient spectra

*nd = not detected (absorption coefficient < -0.1)

Predictor: 0.53 THz peak area

Sample: 1

Dataset \ Method	poly	als	arpls	drpls
set1_ambient_1	48.4	45.2	46.5	42.7
set1_N2_1	50.8	54.0	52.3	55.6
set1_N2_2	47.7	50.1	53.7	59.2
**Mean (set1_N2)**	49.3	52.0	53.0	57.4
**SD (set1_N2)**	2.2	2.8	0.9	2.6
**CV (set1_N2)**	5%	5%	2%	4%
set2_ambient_1	49.1	45.6	42.8	43.1
set2_N2_1	55.4	56.0	53.2	55.8
set2_N2_2	55.3	50.2	56.1	67.8
set2_N2_3	59.1	60.1	58.3	61.7
set2_N2_4	52.1	53.0	57.7	64.1
set2_N2_5	54.2	54.5	54.6	63.4
**Mean (set2_N2)**	55.2	54.8	56.0	62.5
**SD (set2_N2)**	2.6	3.7	2.1	4.4
**CV (set2_N2)**	5%	7%	4%	7%

Sample: 2

Dataset \ Method	poly	als	arpls	drpls
set1_ambient_1	19.0	12.6	15.4	15.8
set1_N2_1	19.5	19.2	14.8	16.2
set1_N2_2	17.3	23.2	15.2	16.5
**Mean (set1_N2)**	18.4	21.2	15.0	16.3
**SD (set1_N2)**	1.5	2.8	0.3	0.3
**CV (set1_N2)**	8%	13%	2%	2%
set2_ambient_1	17.9	14.8	15.7	16.9
set2_N2_1	18.5	23.0	15.1	16.6
set2_N2_2	17.5	27.1	38.2	35.7
set2_N2_3	17.4	24.2	16.3	17.2
set2_N2_4	18.0	26.6	25.0	31.5
set2_N2_5	20.8	21.7	15.8	17.1
**Mean (set2_N2)**	18.4	24.5	22.1	23.6
**SD (set2_N2)**	1.4	2.3	9.9	9.2
**CV (set2_N2)**	8%	9%	45%	39%

Sample: 3

Dataset \ Method	poly	als	arpls	drpls
set1_ambient_1	7.1	nd	nd	nd
set1_N2_1	7.1	4.6	6.7	nd
set1_N2_2	4.8	0.8	nd	6.0
**Mean (set1_N2)**	6.0	2.7	6.7	6.0
**SD (set1_N2)**	1.6	2.7	#DIV/0!	#DIV/0!
**CV (set1_N2)**	27%	101%	#DIV/0!	#DIV/0!
set2_ambient_1	-1.7	nd	nd	7.5
set2_N2_1	-4.1	2.9	nd	5.9
set2_N2_2	-5.4	3.0	-3.8	6.2
set2_N2_3	5.6	3.3	1.7	5.2
set2_N2_4	4.7	4.4	5.9	7.8
set2_N2_5	-4.0	4.6	6.5	nd
**Mean (set2_N2)**	-0.6	3.6	2.6	6.3
**SD (set2_N2)**	5.3	0.8	4.7	1.1
**CV (set2_N2)**	-823%	21%	185%	17%

Predictor: 1.37 THz peak area

Sample: 1

Dataset \ Method	poly	als	arpls	drpls
set1_ambient_1	69.6	73.2	65.2	65.3
set1_N2_1	71.6	65.6	68.6	68.3
set1_N2_2	69.4	61.8	66.9	66.9
**Mean (set1_N2)**	70.5	63.7	67.7	67.6
**SD (set1_N2)**	1.6	2.7	1.2	1.0
**CV (set1_N2)**	2%	4%	2%	1%
set2_ambient_1	70.3	74.1	65.6	67.0
set2_N2_1	73.0	66.1	68.7	68.4
set2_N2_2	71.7	62.9	67.8	67.6
set2_N2_3	70.8	64.8	66.7	66.7
set2_N2_4	71.4	64.6	68.2	68.3
set2_N2_5	74.8	67.0	70.9	70.9
**Mean (set2_N2)**	72.3	65.1	68.5	68.4
**SD (set2_N2)**	1.6	1.6	1.5	1.6
**CV (set2_N2)**	2%	2%	2%	2%

Sample: 2

Dataset \ Method	poly	als	arpls	drpls
set1_ambient_1	22.8	52.2	17.4	16.1
set1_N2_1	22.0	26.6	20.1	20.0
set1_N2_2	21.8	21.9	21.0	20.3
**Mean (set1_N2)**	21.9	24.2	20.6	20.1
**SD (set1_N2)**	0.2	3.4	0.6	0.3
**CV (set1_N2)**	1%	14%	3%	1%
set2_ambient_1	21.9	46.4	18.5	17.3
set2_N2_1	22.1	23.0	20.8	20.8
set2_N2_2	22.2	20.2	21.5	21.3
set2_N2_3	20.0	23.6	20.3	20.0
set2_N2_4	21.6	20.6	21.4	21.1
set2_N2_5	23.6	25.7	21.5	20.9
**Mean (set2_N2)**	21.9	22.6	21.1	20.8
**SD (set2_N2)**	1.3	2.3	0.5	0.5
**CV (set2_N2)**	6%	10%	2%	2%

Sample: 3

Dataset \ Method	poly	als	arpls	drpls
set1_ambient_1	5.6	39.0	3.2	nd
set1_N2_1	3.3	9.1	1.7	nd
set1_N2_2	1.6	16.1	2.3	nd
**Mean (set1_N2)**	2.5	12.6	2.0	#DIV/0!
**SD (set1_N2)**	1.2	4.9	0.4	#DIV/0!
**CV (set1_N2)**	49%	39%	20%	#DIV/0!
set2_ambient_1	2.6	29.9	2.1	nd
set2_N2_1	1.2	10.8	1.9	nd
set2_N2_2	1.0	10.0	2.5	nd
set2_N2_3	2.6	9.0	2.3	nd
set2_N2_4	2.4	8.3	2.2	nd
set2_N2_5	1.6	9.6	2.8	nd
**Mean (set2_N2)**	1.8	9.5	2.3	#DIV/0!
**SD (set2_N2)**	0.7	1.0	0.3	#DIV/0!
**CV (set2_N2)**	38%	10%	14%	#DIV/0!

Predictor: 0.53 THz peak height

Sample: 1

Dataset \ Method	poly	als	arpls	drpls
set1_ambient_1	73.3	73.3	42.7	41.7
set1_N2_1	43.2	73.3	42.8	42.8
set1_N2_2	42.3	73.3	42.4	42.3
**Mean (set1_N2)**	42.8	73.3	42.6	42.5
**SD (set1_N2)**	0.7	0.0	0.3	0.3
**CV (set1_N2)**	2%	0%	1%	1%
set2_ambient_1	49.7	45.3	44.9	43.5
set2_N2_1	44.1	77.8	41.4	41.2
set2_N2_2	43.8	73.3	42.6	42.6
set2_N2_3	43.4	73.3	42.3	41.9
set2_N2_4	77.8	73.3	77.8	77.8
set2_N2_5	77.8	73.3	77.8	77.8
**Mean (set2_N2)**	57.4	74.2	56.4	56.3
**SD (set2_N2)**	18.6	2.0	19.6	19.7
**CV (set2_N2)**	32%	3%	35%	35%

Sample: 2

Dataset \ Method	poly	als	arpls	drpls
set1_ambient_1	73.3	73.3	14.7	14.1
set1_N2_1	15.5	73.3	14.5	14.4
set1_N2_2	15.1	73.3	14.3	14.1
**Mean (set1_N2)**	15.3	73.3	14.4	14.3
**SD (set1_N2)**	0.3	0.0	0.1	0.3
**CV (set1_N2)**	2%	0%	1%	2%
set2_ambient_1	18.1	77.8	15.1	14.5
set2_N2_1	15.4	73.3	14.5	14.4
set2_N2_2	15.1	73.3	17.0	16.6
set2_N2_3	77.8	73.3	77.8	77.8
set2_N2_4	77.8	73.3	77.8	77.8
set2_N2_5	15.6	73.3	14.5	14.3
**Mean (set2_N2)**	40.3	73.3	40.3	40.2
**SD (set2_N2)**	34.2	0.0	34.2	34.4
**CV (set2_N2)**	85%	0%	85%	85%

Sample: 3

Dataset \ Method	poly	als	arpls	drpls
set1_ambient_1	73.3	nd	nd	nd
set1_N2_1	4.8	73.3	5.1	nd
set1_N2_2	3.9	73.3	nd	5.1
**Mean (set1_N2)**	4.3	73.3	5.1	5.1
**SD (set1_N2)**	0.6	0.0	#DIV/0!	#DIV/0!
**CV (set1_N2)**	14%	0%	#DIV/0!	#DIV/0!
set2_ambient_1	nd	nd	nd	5.8
set2_N2_1	nd	73.3	nd	5.0
set2_N2_2	nd	73.3	nd	5.1
set2_N2_3	77.8	73.3	77.8	77.8
set2_N2_4	77.8	73.3	77.8	77.8
set2_N2_5	nd	73.3	5.0	nd
**Mean (set2_N2)**	77.8	73.3	53.5	41.4
**SD (set2_N2)**	0.0	0.0	42.0	42.0
**CV (set2_N2)**	0%	0%	78%	101%

Predictor: 1.37 THz peak height

Sample: 1

Dataset \ Method	poly	als	arpls	drpls
set1_ambient_1	75.3	73.3	74.1	74.1
set1_N2_1	74.7	73.3	76.5	74.5
set1_N2_2	74.9	73.3	73.9	73.9
**Mean (set1_N2)**	74.8	73.3	75.2	74.2
**SD (set1_N2)**	0.1	0.0	1.8	0.4
**CV (set1_N2)**	0%	0%	2%	1%
set2_ambient_1	75.8	73.3	74.7	75.0
set2_N2_1	74.7	73.3	74.7	74.5
set2_N2_2	75.8	73.3	74.3	74.5
set2_N2_3	73.3	73.3	72.4	72.4
set2_N2_4	75.7	73.3	74.4	74.4
set2_N2_5	74.7	73.3	74.5	77.0
**Mean (set2_N2)**	74.8	73.3	74.0	74.6
**SD (set2_N2)**	1.0	0.0	0.9	1.7
**CV (set2_N2)**	1%	0%	1%	2%

Sample: 2

Dataset \ Method	poly	als	arpls	drpls
set1_ambient_1	21.2	73.6	20.5	20.3
set1_N2_1	74.7	73.3	678.3	20.0
set1_N2_2	20.0	73.3	20.0	19.9
**Mean (set1_N2)**	47.4	73.3	349.1	19.9
**SD (set1_N2)**	38.6	0.0	465.5	0.1
**CV (set1_N2)**	82%	0%	133%	0%
set2_ambient_1	22.7	73.3	20.2	20.3
set2_N2_1	74.7	73.3	74.7	20.2
set2_N2_2	20.3	73.3	19.8	74.5
set2_N2_3	73.3	73.3	18.8	19.0
set2_N2_4	20.1	73.3	19.8	20.0
set2_N2_5	74.7	73.3	74.5	20.7
**Mean (set2_N2)**	52.6	73.3	41.5	30.9
**SD (set2_N2)**	29.6	0.0	30.2	24.4
**CV (set2_N2)**	56%	0%	73%	79%

Sample: 3

Dataset \ Method	poly	als	arpls	drpls
set1_ambient_1	2.8	70.2	nd	nd
set1_N2_1	74.6	-2.09E+18	nd	nd
set1_N2_2	nd	73.3	nd	nd
**Mean (set1_N2)**	74.6	-1.05E+18	#DIV/0!	#DIV/0!
**SD (set1_N2)**	#DIV/0!	1.48E+18	#DIV/0!	#DIV/0!
**CV (set1_N2)**	#DIV/0!	-141%	#DIV/0!	#DIV/0!
set2_ambient_1	nd	73.3	nd	nd
set2_N2_1	nd	-2.09E+08	nd	nd
set2_N2_2	nd	73.3	nd	nd
set2_N2_3	72.7	-1.24E+07	nd	nd
set2_N2_4	nd	73.3	nd	nd
set2_N2_5	nd	73.3	nd	nd
**Mean (set2_N2)**	72.7	-4.43E+07	#DIV/0!	#DIV/0!
**SD (set2_N2)**	#DIV/0!	9.22E+07	#DIV/0!	#DIV/0!
**CV (set2_N2)**	#DIV/0!	-208%	#DIV/0!	#DIV/0!
