# Supplementary material for: Terahertz Spectroscopic Analysis of Lactose in Infant Formula: Implications for Detection and Quantification
Source: Molecules. 2022 Aug 8;27(15):5040. doi: 10.3390/molecules27155040 (PMC9370465; doi:10.3390/molecules27155040)
Supplement: Supplementary file 1 [file molecules-27-05040-s001.zip › data/absorption_spectra_prediction.pdf]

DOI: 10.3390/molecules27155040

***r*^{2} values**

Predictor: **0.53 THz peak area**

Dataset \ Method	poly	als	arpls	drpls
220311	0.9846	0.9921	0.9905	0.9892
220311_N	0.9776	0.9757	0.9881	0.9861
220311_N2	0.9701	0.9809	0.9655	0.9744
220315	0.9803	0.9979	0.9958	0.9960
220315_N1	0.9719	0.9659	0.9778	0.9666
220315_N2	0.9733	0.9797	0.9735	0.9716
220315_N3	0.9766	0.9636	0.9462	0.9482
220315_N4	0.9813	0.9766	0.9794	0.9827
220315_N5	0.9773	0.9913	0.9883	0.9923

Predictor: **0.53 THz peak height**

Dataset \ Method	poly	als	arpls	drpls
220311	0.9222	0.9299	0.9282	0.9260
220311_N	0.9199	0.9435	0.9283	0.9300
220311_N2	0.9231	0.9527	0.9305	0.9402
220315	0.9236	0.9361	0.9364	0.9389
220315_N1	0.9000	0.9318	0.9079	0.9092
220315_N2	0.9254	0.9511	0.9357	0.9385
220315_N3	0.9296	0.9568	0.9399	0.9444
220315_N4	0.9251	0.9441	0.9310	0.9329
220315_N5	0.9214	0.9459	0.9302	0.9334

Predictor: **1.37 THz peak area**

Dataset \ Method	poly	als	arpls	drpls
220311	0.9518	0.9713	0.9660	0.9647
220311_N	0.9468	0.9658	0.9619	0.9658
220311_N2	0.9595	0.9812	0.9747	0.9754
220315	0.9661	0.1631	0.9742	0.9785
220315_N1	0.9603	0.9743	0.9720	0.9705
220315_N2	0.9569	0.9790	0.9680	0.9722
220315_N3	0.9341	0.9570	0.9542	0.9570
220315_N4	0.9451	0.9660	0.9650	0.9714
220315_N5	0.9423	0.9671	0.9626	0.9697

Predictor: **1.37 THz peak height**

Dataset \ Method	poly	als	arpls	drpls
220311	0.8444	0.9424	0.8617	0.8476
220311_N	0.8444	0.9008	0.8906	0.8863
220311_N2	0.8734	0.9153	0.9143	0.9071
220315	0.8692	0.8452	0.8936	0.8870
220315_N1	0.8578	0.9187	0.9029	0.8969
220315_N2	0.8697	0.9200	0.7965	0.9021
220315_N3	0.8057	0.8629	0.8708	0.8616
220315_N4	0.8256	0.8836	0.8768	0.8777
220315_N5	0.8459	0.8967	0.8886	0.8885

DOI: 10.3390/molecules27155040

RMSE values

Predictor: **0.53 THz peak area**

Dataset \ Method	poly	als	arpls	drpls
220311	0.0011	0.0012	0.0011	0.0012
220311_N	0.0014	0.0021	0.0013	0.0015
220311_N2	0.0016	0.0020	0.0025	0.0021
220315	0.0014	0.0007	0.0007	0.0007
220315_N1	0.0014	0.0023	0.0017	0.0022
220315_N2	0.0015	0.0020	0.0021	0.0023
220315_N3	0.0014	0.0025	0.0029	0.0028
220315_N4	0.0013	0.0020	0.0018	0.0017
220315_N5	0.0014	0.0013	0.0013	0.0012

Predictor: **0.53 THz peak height**

Dataset \ Method	poly	als	arpls	drpls
220311	0.0662	0.0671	0.0690	0.0706
220311_N	0.0687	0.0594	0.0689	0.0684
220311_N2	0.0682	0.0549	0.0699	0.0633
220315	0.0772	0.0675	0.0648	0.0631
220315_N1	0.0725	0.0598	0.0734	0.0729
220315_N2	0.0671	0.0552	0.0662	0.0647
220315_N3	0.0653	0.0520	0.0641	0.0609
220315_N4	0.0660	0.0591	0.0675	0.0666
220315_N5	0.0677	0.0578	0.0678	0.0664

Predictor: **1.37 THz peak area**

Dataset \ Method	poly	als	arpls	drpls
220311	0.0039	0.0056	0.0042	0.0044
220311_N	0.0040	0.0051	0.0055	0.0050
220311_N2	0.0037	0.0039	0.0047	0.0044
220315	0.0033	0.0053	0.0036	0.0035
220315_N1	0.0034	0.0044	0.0047	0.0046
220315_N2	0.0038	0.0042	0.0052	0.0047
220315_N3	0.0047	0.0061	0.0063	0.0059
220315_N4	0.0041	0.0051	0.0053	0.0046
220315_N5	0.0042	0.0051	0.0054	0.0047

Predictor: **1.37 THz peak height**

Dataset \ Method	poly	als	arpls	drpls
220311	0.0807	0.0610	0.0832	0.0925
220311_N	0.0805	0.0698	0.0814	0.0842
220311_N2	0.0787	0.0749	0.0778	0.0804
220315	0.0857	0.0433	0.0757	0.0833
220315_N1	0.0793	0.0677	0.0784	0.0796
220315_N2	0.0762	0.0719	0.1135	0.0835
220315_N3	0.0965	0.0950	0.0958	0.0994
220315_N4	0.0908	0.0826	0.0909	0.0890
220315_N5	0.0850	0.0800	0.0860	0.0870

DOI: 10.3390/molecules27155040

Concentration of α-lactose monohydrate

Predicted concentration by absorption spectra

*nd = not detected (absorbance ≤ -0.1 or absorbance ≥ 1)

Predictor: **0.53 THz peak area**

Sample: 1

Method / Dataset	poly	als	arpls	drpls
set1_ambient_1	44.7	41.3	43.4	40.3
set1_N2_1	48.2	34.8	49.3	38.8
set1_N2_2	48.7	35.3	47.9	37.3
**Mean (set1_N2)**	48.5	35.1	48.6	38.1
**SD (set1_N2)**	0.4	0.4	1.0	1.1
**CV (set1_N2)**	1%	1%	2%	3%
set2_ambient_1	50.3	43.6	43.1	38.5
set2_N2_1	47.6	32.9	49.3	41.2
set2_N2_2	49.1	36.1	48.3	38.5
set2_N2_3	49.9	35.2	50.0	40.8
set2_N2_4	49.4	36.9	50.2	41.5
set2_N2_5	49.0	36.2	52.5	43.2
**Mean (set2_N2)**	49.0	35.5	50.1	41.0
**SD (set2_N2)**	0.9	1.6	1.6	1.7
**CV (set2_N2)**	2%	4%	3%	4%

Sample: 2

Method / Dataset	poly	als	arpls	drpls
set1_ambient	6.8	12.1	7.6	-12.8
set1_N2_1	11.4	4.9	8.8	2.6
set1_N2_2	11.7	3.3	10.1	0.5
**Mean (set1_N**	11.6	4.1	9.5	1.6
**SD (set1_N2)**	0.2	1.1	0.9	1.5
**CV (set1_N2)**	2%	28%	10%	96%
set2_ambient	14.3	13.6	8.9	5.2
set2_N2_1	11.4	2.0	9.3	1.9
set2_N2_2	11.9	3.6	8.5	1.1
set2_N2_3	13.5	-1.0	8.0	0.8
set2_N2_4	12.3	4.4	9.8	2.6
set2_N2_5	12.9	5.3	11.7	3.3
**Mean (set2_N**	12.4	2.9	9.5	1.9
**SD (set2_N2)**	0.8	2.5	1.4	1.0
**CV (set2_N2)**	7%	87%	15%	53%

Sample: 3

Method / Dataset	poly	als	arpls	drpls
set1_ambient	-5.0	1.9	-14.5	-20.8
set1_N2_1	-3.0	-8.4	-12.4	-19.1
set1_N2_2	-2.5	-9.0	-12.0	-20.2
**Mean (set1_N**	-2.8	-8.7	-12.2	-19.7
**SD (set1_N2)**	0.4	0.4	0.3	0.8
**CV (set1_N2)**	-13%	-5%	-2%	-4%
set2_ambient	-10.6	2.8	-12.8	-19.4
set2_N2_1	-3.3	-11.2	-12.9	-19.8
set2_N2_2	-3.3	-11.3	-14.8	-20.3
set2_N2_3	-3.1	-15.0	-13.7	-23.0
set2_N2_4	-3.6	-8.8	-13.0	-20.3
set2_N2_5	-2.9	-8.8	-12.3	-18.8
**Mean (set2_N**	-3.2	-11.0	-13.3	-20.4
**SD (set2_N2)**	0.3	2.5	1.0	1.6
**CV (set2_N2)**	-8%	-23%	-7%	-8%

Predictor: **1.37 THz peak area**

Sample: 1

Method / Dataset	poly	als	arpls	drpls
set1_ambient_1	41.1	47.6	38.6	41.4
set1_N2_1	42.9	49.2	43.4	47.2
set1_N2_2	41.5	49.0	41.2	45.1
**Mean (set1_N2)**	42.2	49.1	42.3	46.2
**SD (set1_N2)**	1.0	0.1	1.6	1.5
**CV (set1_N2)**	2%	0%	4%	3%
set2_ambient_1	45.2	-154.0	38.6	40.8
set2_N2_1	40.4	44.9	41.4	45.6
set2_N2_2	42.3	48.4	42.8	46.5
set2_N2_3	44.2	46.1	44.0	47.9
set2_N2_4	43.5	48.7	44.0	47.9
set2_N2_5	44.8	49.0	46.1	49.7
**Mean (set2_N2)**	43.0	47.4	43.7	47.5
**SD (set2_N2)**	1.7	1.8	1.7	1.6
**CV (set2_N2)**	4%	4%	4%	3%

Sample: 2

Method / Dataset	poly	als	arpls	drpls
set1_ambient	1.6	34.7	4.3	9.2
set1_N2_1	2.3	34.4	5.6	11.3
set1_N2_2	3.4	31.9	6.7	12.1
**Mean (set1_N**	2.9	33.2	6.2	11.7
**SD (set1_N2)**	0.8	1.8	0.8	0.6
**CV (set1_N2)**	27%	5%	13%	5%
set2_ambient	-0.4	-114.0	7.0	11.7
set2_N2_1	3.0	30.5	6.0	10.9
set2_N2_2	3.8	32.4	6.8	12.6
set2_N2_3	1.9	31.3	5.8	11.5
set2_N2_4	2.6	33.3	6.4	12.2
set2_N2_5	4.1	33.7	7.1	12.6
**Mean (set2_N**	3.1	32.2	6.4	12.0
**SD (set2_N2)**	0.9	1.3	0.5	0.7
**CV (set2_N2)**	29%	4%	8%	6%

Sample: 3

Method / Dataset	poly	als	arpls	drpls
set1_ambient	-8.6	32.4	-4.4	0.6
set1_N2_1	-10.8	25.2	-4.9	0.4
set1_N2_2	-9.6	26.1	-4.3	0.6
**Mean (set1_N**	-10.2	25.7	-4.6	0.5
**SD (set1_N2)**	0.8	0.6	0.4	0.1
**CV (set1_N2)**	-8%	2%	-9%	28%
set2_ambient	-9.3	-196.0	-2.5	2.3
set2_N2_1	-9.8	23.2	-4.4	0.1
set2_N2_2	-9.8	25.0	-4.8	0.7
set2_N2_3	-11.7	22.4	-6.0	-0.9
set2_N2_4	-11.6	25.1	-5.6	-0.1
set2_N2_5	-10.4	25.3	-5.0	0.4
**Mean (set2_N**	-10.7	24.2	-5.2	0.0
**SD (set2_N2)**	0.9	1.3	0.6	0.6
**CV (set2_N2)**	-9%	5%	-12%	1517%

Predictor: **0.53 THz peak height**

Sample: 1

Method / Dataset	poly	als	arpls	drpls
set1_ambient_1	45.8	45.0	47.3	46.5
set1_N2_1	48.5	45.4	48.3	46.3
set1_N2_2	48.1	45.8	48.3	45.8
**Mean (set1_N2)**	48.3	45.6	48.3	46.1
**SD (set1_N2)**	0.3	0.3	0.0	0.4
**CV (set1_N2)**	1%	1%	0%	1%
set2_ambient_1	52.3	47.7	47.3	45.8
set2_N2_1	46.7	43.6	46.8	45.6
set2_N2_2	48.1	45.2	47.7	46.2
set2_N2_3	49.2	46.5	49.2	47.3
set2_N2_4	48.6	46.5	48.6	46.9
set2_N2_5	48.6	45.7	48.8	47.2
**Mean (set2_N2)**	48.2	45.5	48.2	46.6
**SD (set2_N2)**	0.9	1.2	1.0	0.7
**CV (set2_N2)**	2%	3%	2%	2%

Sample: 2

Method / Dataset	poly	als	arpls	drpls
set1_ambient	5.0	5.7	8.8	-20.9
set1_N2_1	10.1	5.9	9.3	6.0
set1_N2_2	10.5	7.2	10.7	6.2
**Mean (set1_N**	10.3	6.6	10.0	6.1
**SD (set1_N2)**	0.3	0.9	1.0	0.1
**CV (set1_N2)**	3%	14%	10%	2%
set2_ambient	15.5	11.3	10.0	7.1
set2_N2_1	9.7	4.3	9.1	5.9
set2_N2_2	10.5	6.1	9.6	7.0
set2_N2_3	10.3	6.5	9.6	6.2
set2_N2_4	9.5	7.0	8.9	5.5
set2_N2_5	10.0	6.6	9.4	6.5
**Mean (set2_N**	10.0	6.1	9.3	6.2
**SD (set2_N2)**	0.4	1.1	0.3	0.6
**CV (set2_N2)**	4%	17%	3%	9%

Sample: 3

Method / Dataset	poly	als	arpls	drpls
set1_ambient	-11.6	nd	-20.1	-21.8
set1_N2_1	-7.8	nd	-18.8	-21.5
set1_N2_2	-6.7	32.0	-17.3	-22.4
**Mean (set1_N**	-7.3	32.0	-18.1	-22.0
**SD (set1_N2)**	0.8	#DIV/0!	1.1	0.6
**CV (set1_N2)**	-11%	#DIV/0!	-6%	-3%
set2_ambient	-10.7	-5.6	-17.9	-20.8
set2_N2_1	-8.2	nd	-19.1	-21.8
set2_N2_2	-7.4	-10.4	-19.4	-22.2
set2_N2_3	-8.1	-12.2	-17.1	-20.5
set2_N2_4	-9.7	nd	-20.2	-23.0
set2_N2_5	-7.7	9.1	-18.7	-21.9
**Mean (set2_N**	-8.2	-4.5	-18.9	-21.9
**SD (set2_N2)**	0.9	11.8	1.1	0.9
**CV (set2_N2)**	-11%	-262%	-6%	-4%

Predictor: **1.37 THz peak height**

Sample: 1

Method / Dataset	poly	als	arpls	drpls
set1_ambient_1	40.8	41.4	38.8	41.1
set1_N2_1	41.1	39.2	40.4	43.3
set1_N2_2	41.8	41.9	40.4	42.2
**Mean (set1_N2)**	41.5	40.6	40.4	42.8
**SD (set1_N2)**	0.5	1.9	0.0	0.8
**CV (set1_N2)**	1%	5%	0%	2%
set2_ambient_1	47.0	9.8	41.4	43.5
set2_N2_1	39.2	38.5	38.8	40.5
set2_N2_2	40.6	41.6	36.8	43.4
set2_N2_3	42.0	40.6	42.1	44.0
set2_N2_4	43.1	41.2	42.1	43.5
set2_N2_5	43.8	42.3	42.4	45.1
**Mean (set2_N2)**	41.7	40.8	40.4	43.3
**SD (set2_N2)**	1.9	1.4	2.5	1.7
**CV (set2_N2)**	4%	4%	6%	4%

Sample: 2

Method / Dataset	poly	als	arpls	drpls
set1_ambient	-7.1	15.1	-6.8	-3.0
set1_N2_1	-7.1	5.3	-4.6	0.2
set1_N2_2	-1.8	11.0	0.2	2.4
**Mean (set1_N**	-4.5	8.2	-2.2	1.3
**SD (set1_N2)**	3.7	4.0	3.4	1.6
**CV (set1_N2)**	-84%	49%	-154%	120%
set2_ambient	-9.2	-23.8	-1.4	3.0
set2_N2_1	-3.4	8.6	-1.3	0.2
set2_N2_2	-5.2	10.7	-9.2	3.5
set2_N2_3	-8.9	6.8	-3.6	-0.6
set2_N2_4	-3.5	8.2	-1.4	0.2
set2_N2_5	-2.6	10.2	-2.2	2.5
**Mean (set2_N**	-4.7	8.9	-3.5	1.2
**SD (set2_N2)**	2.5	1.6	3.3	1.7
**CV (set2_N2)**	-53%	18%	-93%	151%

Sample: 3

Method / Dataset	poly	als	arpls	drpls
set1_ambient	129.0	nd	-20.7	nd
set1_N2_1	-26.0	-3.3	-18.0	nd
set1_N2_2	-19.4	2.4	-13.9	-10.6
**Mean (set1_N**	-22.7	-0.5	-16.0	-10.6
**SD (set1_N2)**	4.7	4.0	2.9	#DIV/0!
**CV (set1_N2)**	-21%	-896%	-18%	#DIV/0!
set2_ambient	-16.0	nd	-16.3	-9.7
set2_N2_1	-20.8	-0.5	-14.3	-8.2
set2_N2_2	-24.4	0.8	-25.8	-9.9
set2_N2_3	-27.9	-4.1	-14.6	-9.7
set2_N2_4	-22.2	-2.3	-16.2	-13.2
set2_N2_5	nd	-0.5	-17.3	-11.3
**Mean (set2_N**	-23.8	-1.3	-17.6	-10.5
**SD (set2_N2)**	3.1	1.9	4.7	1.9
**CV (set2_N2)**	-13%	-144%	-27%	-18%

DOI: 10.3390/molecules27155040

Concentration of lactose anhydrous (≈95% of α-lactose monohydrate)

HPLC quantification (by external accredited laboratories)

		Sample 1	Sample 2
AOAC (2019) 980.13	1	**62.70**	**60.46**
	2	**61.24**	**62.52**
		Ref. No. SFC 1020/2565	Ref No. SFC 0823/2565
		27-Jun-22	11-Apr-22
AOAC (2019) 982.14	3	**not done**	**56.92**
			Ref No. 6501.1911/651949
			1-Apr-22
Mean		**61.97**	**59.97**

Predicted concentration by absorption spectra

*nd = not detected (absorbance ≤ -0.1 or absorbance ≥ 1)

Predictor: **0.53 THz peak area**

Sample: **1**

Dataset \ Method	poly	als	arpls	drpls
set1_ambient_1	42.5	39.2	41.2	38.3
set1_N2_1	45.8	33.1	46.8	36.9
set1_N2_2	46.3	33.5	45.5	35.4
**Mean (set1_N2)**	46.0	33.3	46.2	36.1
**SD (set1_N2)**	0.3	0.3	0.9	1.0
**CV (set1_N2)**	1%	1%	2%	3%
set2_ambient_1	47.8	41.4	40.9	36.6
set2_N2_1	45.2	31.3	46.8	39.1
set2_N2_2	46.6	34.3	45.9	36.6
set2_N2_3	47.4	33.4	47.5	38.8
set2_N2_4	46.9	35.1	47.7	39.4
set2_N2_5	46.6	34.4	49.9	41.0
**Mean (set2_N2)**	46.6	33.7	47.6	39.0
**SD (set2_N2)**	0.8	1.5	1.5	1.6
**CV (set2_N2)**	2%	4%	3%	4%

Sample: **2**

Dataset \ Method	poly	als	arpls	drpls
set1_ambient_1	6.5	11.5	7.2	-12.2
set1_N2_1	10.8	4.7	8.4	2.5
set1_N2_2	11.1	3.1	9.6	0.5
**Mean (set1_N2)**	11.0	3.9	9.0	1.5
**SD (set1_N2)**	0.2	1.1	0.9	1.4
**CV (set1_N2)**	2%	28%	10%	96%
set2_ambient_1	13.6	12.9	8.5	4.9
set2_N2_1	10.8	1.9	8.8	1.8
set2_N2_2	11.3	3.4	8.1	1.0
set2_N2_3	12.8	-1.0	7.6	0.8
set2_N2_4	11.7	4.2	9.3	2.5
set2_N2_5	12.3	5.0	11.1	3.1
**Mean (set2_N2)**	11.8	2.7	9.0	1.8
**SD (set2_N2)**	0.8	2.4	1.4	1.0
**CV (set2_N2)**	7%	87%	15%	53%

Sample: **3**

Dataset \ Method	poly	als	arpls	drpls
set1_ambient_1	-4.8	1.8	-13.8	-19.8
set1_N2_1	-2.9	-8.0	-11.8	-18.1
set1_N2_2	-2.4	-8.6	-11.4	-19.2
**Mean (set1_N2)**	-2.6	-8.3	-11.6	-18.7
**SD (set1_N2)**	0.3	0.4	0.3	0.7
**CV (set1_N2)**	-13%	-5%	-2%	-4%
set2_ambient_1	-10.1	2.7	-12.2	-18.4
set2_N2_1	-3.1	-10.6	-12.3	-18.8
set2_N2_2	-3.1	-10.7	-14.1	-19.3
set2_N2_3	-2.9	-14.3	-13.0	-21.9
set2_N2_4	-3.4	-8.4	-12.4	-19.3
set2_N2_5	-2.8	-8.4	-11.7	-17.9
**Mean (set2_N2)**	-3.1	-10.5	-12.7	-19.4
**SD (set2_N2)**	0.2	2.4	0.9	1.5
**CV (set2_N2)**	-8%	-23%	-7%	-8%

Predictor: **1.37 THz peak area**

Sample: **1**

Dataset \ Method	poly	als	arpls	drpls
set1_ambient_1	39.0	45.2	36.7	39.3
set1_N2_1	40.8	46.7	41.2	44.8
set1_N2_2	39.4	46.6	39.1	42.8
**Mean (set1_N2)**	40.1	46.6	40.2	43.8
**SD (set1_N2)**	0.9	0.1	1.5	1.4
**CV (set1_N2)**	2%	0%	4%	3%
set2_ambient_1	42.9	-146.3	36.7	38.8
set2_N2_1	38.4	42.7	39.3	43.3
set2_N2_2	40.2	46.0	40.7	44.2
set2_N2_3	42.0	43.8	41.8	45.5
set2_N2_4	41.3	46.3	41.8	45.5
set2_N2_5	42.6	46.6	43.8	47.2
**Mean (set2_N2)**	40.9	45.0	41.5	45.1
**SD (set2_N2)**	1.7	1.7	1.6	1.5
**CV (set2_N2)**	4%	4%	4%	3%

Sample: **2**

Dataset \ Method	poly	als	arpls	drpls
set1_ambient_1	1.5	33.0	4.1	8.7
set1_N2_1	2.2	32.7	5.3	10.7
set1_N2_2	3.2	30.3	6.4	11.5
**Mean (set1_N2)**	2.7	31.5	5.8	11.1
**SD (set1_N2)**	0.7	1.7	0.7	0.5
**CV (set1_N2)**	27%	5%	13%	5%
set2_ambient_1	-0.4	-108.3	6.7	11.1
set2_N2_1	2.9	29.0	5.7	10.4
set2_N2_2	3.6	30.8	6.5	12.0
set2_N2_3	1.8	29.7	5.5	10.9
set2_N2_4	2.5	31.6	6.1	11.6
set2_N2_5	3.9	32.0	6.7	12.0
**Mean (set2_N2)**	2.9	30.6	6.1	11.4
**SD (set2_N2)**	0.8	1.3	0.5	0.7
**CV (set2_N2)**	29%	4%	8%	6%

Sample: **3**

Dataset \ Method	poly	als	arpls	drpls
set1_ambient_1	-8.2	30.8	-4.2	0.6
set1_N2_1	-10.3	23.9	-4.7	0.4
set1_N2_2	-9.1	24.8	-4.1	0.6
**Mean (set1_N2)**	-9.7	24.4	-4.4	0.5
**SD (set1_N2)**	0.8	0.6	0.4	0.1
**CV (set1_N2)**	-8%	2%	-9%	28%
set2_ambient_1	-8.8	-186.2	-2.4	2.2
set2_N2_1	-9.3	22.0	-4.2	0.1
set2_N2_2	-9.3	23.8	-4.6	0.7
set2_N2_3	-11.1	21.3	-5.7	-0.9
set2_N2_4	-11.0	23.8	-5.3	-0.1
set2_N2_5	-9.9	24.0	-4.8	0.4
**Mean (set2_N2)**	-10.1	23.0	-4.9	0.0
**SD (set2_N2)**	0.9	1.2	0.6	0.6
**CV (set2_N2)**	-9%	5%	-12%	1517%

Predictor: **0.53 THz peak height**

Sample: **1**

Dataset \ Method	poly	als	arpls	drpls
set1_ambient_1	43.5	42.8	44.9	44.2
set1_N2_1	46.1	43.1	45.9	44.0
set1_N2_2	45.7	43.5	45.9	43.5
**Mean (set1_N2)**	45.9	43.3	45.9	43.7
**SD (set1_N2)**	0.3	0.3	0.0	0.3
**CV (set1_N2)**	1%	1%	0%	1%
set2_ambient_1	49.7	45.3	44.9	43.5
set2_N2_1	44.4	41.4	44.5	43.3
set2_N2_2	45.7	42.9	45.3	43.9
set2_N2_3	46.7	44.2	46.7	44.9
set2_N2_4	46.2	44.2	46.2	44.6
set2_N2_5	46.2	43.4	46.4	44.8
**Mean (set2_N2)**	45.8	43.2	45.8	44.3
**SD (set2_N2)**	0.9	1.1	0.9	0.7
**CV (set2_N2)**	2%	3%	2%	2%

Sample: **2**

Dataset \ Method	poly	als	arpls	drpls
set1_ambient_1	4.8	5.4	8.4	-19.9
set1_N2_1	9.6	5.6	8.8	5.7
set1_N2_2	10.0	6.8	10.2	5.9
**Mean (set1_N2)**	9.8	6.2	9.5	5.8
**SD (set1_N2)**	0.3	0.9	0.9	0.1
**CV (set1_N2)**	3%	14%	10%	2%
set2_ambient_1	14.7	10.7	9.5	6.7
set2_N2_1	9.2	4.1	8.6	5.6
set2_N2_2	10.0	5.8	9.1	6.7
set2_N2_3	9.8	6.2	9.1	5.9
set2_N2_4	9.0	6.7	8.5	5.2
set2_N2_5	9.5	6.3	8.9	6.2
**Mean (set2_N2)**	9.5	5.8	8.9	5.9
**SD (set2_N2)**	0.4	1.0	0.3	0.5
**CV (set2_N2)**	4%	17%	3%	9%

Sample: **3**

Dataset \ Method	poly	als	arpls	drpls
set1_ambient_1	-11.0	nd	-19.1	-20.7
set1_N2_1	-7.4	nd	-17.9	-20.4
set1_N2_2	-6.4	30.4	-16.4	-21.3
**Mean (set1_N2)**	-6.9	30.4	-17.1	-20.9
**SD (set1_N2)**	0.7	#DIV/0!	1.0	0.6
**CV (set1_N2)**	-11%	#DIV/0!	-6%	-3%
set2_ambient_1	-10.2	-5.3	-17.0	-19.8
set2_N2_1	-7.8	nd	-18.1	-20.7
set2_N2_2	-7.0	-9.9	-18.4	-21.1
set2_N2_3	-7.7	-11.6	-16.2	-19.5
set2_N2_4	-9.2	nd	-19.2	-21.9
set2_N2_5	-7.3	8.6	-17.8	-20.8
**Mean (set2_N2)**	-7.8	-4.3	-18.0	-20.8
**SD (set2_N2)**	0.8	11.2	1.1	0.9
**CV (set2_N2)**	-11%	-262%	-6%	-4%

Predictor: **1.37 THz peak height**

Sample: **1**

Dataset \ Method	poly	als	arpls	drpls
set1_ambient_1	38.8	39.3	36.9	39.0
set1_N2_1	39.0	37.2	38.4	41.1
set1_N2_2	39.7	39.8	38.4	40.1
**Mean (set1_N2)**	39.4	38.5	38.4	40.6
**SD (set1_N2)**	0.5	1.8	0.0	0.7
**CV (set1_N2)**	1%	5%	0%	2%
set2_ambient_1	44.7	9.3	39.3	41.3
set2_N2_1	37.2	36.6	36.9	38.5
set2_N2_2	38.6	39.5	35.0	41.2
set2_N2_3	39.9	38.6	40.0	41.8
set2_N2_4	40.9	39.1	40.0	41.3
set2_N2_5	41.6	40.2	40.3	42.8
**Mean (set2_N2)**	39.7	38.8	38.4	41.1
**SD (set2_N2)**	1.8	1.4	2.4	1.6
**CV (set2_N2)**	4%	4%	6%	4%

Sample: **2**

Dataset \ Method	poly	als	arpls	drpls
set1_ambient_1	-6.7	14.3	-6.5	-2.9
set1_N2_1	-6.7	5.0	-4.4	0.2
set1_N2_2	-1.7	10.5	0.2	2.3
**Mean (set1_N2)**	-4.2	7.7	-2.1	1.2
**SD (set1_N2)**	3.6	3.8	3.2	1.5
**CV (set1_N2)**	-84%	49%	-154%	120%
set2_ambient_1	-8.7	-22.6	-1.3	2.9
set2_N2_1	-3.2	8.2	-1.2	0.2
set2_N2_2	-4.9	10.2	-8.7	3.3
set2_N2_3	-8.5	6.5	-3.4	-0.6
set2_N2_4	-3.3	7.8	-1.3	0.2
set2_N2_5	-2.5	9.7	-2.1	2.4
**Mean (set2_N2)**	-4.5	8.5	-3.4	1.1
**SD (set2_N2)**	2.4	1.5	3.1	1.7
**CV (set2_N2)**	-53%	18%	-93%	151%

Sample: **3**

Dataset \ Method	poly	als	arpls	drpls
set1_ambient_1	122.6	nd	-19.7	nd
set1_N2_1	-24.7	-3.1	-17.1	nd
set1_N2_2	-18.4	2.3	-13.2	-10.1
**Mean (set1_N2)**	-21.6	-0.4	-15.2	-10.1
**SD (set1_N2)**	4.4	3.8	2.8	#DIV/0!
**CV (set1_N2)**	-21%	-896%	-18%	#DIV/0!
set2_ambient_1	-15.2	nd	-15.5	-9.2
set2_N2_1	-19.8	-0.5	-13.6	-7.8
set2_N2_2	-23.2	0.8	-24.5	-9.4
set2_N2_3	-26.5	-3.9	-13.9	-9.2
set2_N2_4	-21.1	-2.2	-15.4	-12.5
set2_N2_5	nd	-0.5	-16.4	-10.7
**Mean (set2_N2)**	-22.6	-1.3	-16.8	-9.9
**SD (set2_N2)**	2.9	1.8	4.5	1.8
**CV (set2_N2)**	-13%	-144%	-27%	-18%
