# Supplementary material for: Terahertz Spectroscopic Analysis of Lactose in Infant Formula: Implications for Detection and Quantification
Source: Molecules. 2022 Aug 8;27(15):5040. doi: 10.3390/molecules27155040 (PMC9370465; doi:10.3390/molecules27155040)
Supplement: Supplementary file 1 [file molecules-27-05040-s001.zip › index, supplementary table and figues.pdf]

SUPPLEMENTARY MATERIALS

Terahertz Spectroscopic Analysis of Lactose in Infant Formula: Implications for Detection and Quantification

Sopanant Datta [1], Kiattiwut Prasertsuk [2], Nuttawat Khammata [3], Patharakorn Rattanawan [2], Jia Yi Chia [2], Rungroj Jintamethasawat [2], Thawatchart Chulapakorn [2,4] and Taweetham Limpanuparb [1,*]

[1] Science Division, Mahidol University International College, Mahidol University, Salaya, Phutthamonthon, Nakhon Pathom, 73170, Thailand; sopanant.dat@student.mahidol.edu (S.D.)
[2] National Electronics and Computer Technology, National Science and Technology Development Agency, 112 Thailand Science Park, Pathum Thani, 12120, Thailand; kiattiwut.prasertsuk@nectec.or.th (K.P.); patharakorn.rattanawan@nectec.or.th (P.R.); jiayi.chia@nectec.or.th (J.Y.C.); rungroj.jintamethasawat@nectec.or.th (R.J.)
[3] Department of Physics and Materials Science, Faculty of Science, Chiang Mai University, Chiang Mai, 50200, Thailand; nuttawat_kh@cmu.ac.th
[4] Department of Construction Sciences, Lund, University, Lund, 22100, Sweden; thawatchart.chulapakorn@solid.lth.se
*****Correspondence: taweetham.lim@mahidol.edu

Table and figures in this document:

- Table S1 Pellet mass and thickness of pure α-lactose monohydrate standards and infant formula samples for THz-TDS measurement
- Figure S1 Custom-made THz-TDS system in this study at National Electronics and Computer Technology Centre, Pathum Thani, Thailand (a zoom-in of the sample holder is framed in red)
- Figure S2 Product label of infant formula Sample 1
- Figure S3 Product label of infant formula Sample 2
- Figure S4 Product label of infant formula Sample 3

Items in separate folders (For convenience, PDF files of spreadsheet and codes are also included in addition to original files.):

1. **data**
   a. **filelist.csv** contains a list of samples used in this study and their corresponding file names
   b. Nine dataset folders contain data from each replicate of THz-TDS measurement.

Set 1 (sampling rate = 250 times/measurement)	Set 2 (sampling rate = 500 times/measurement)
Ambient condition:	Ambient condition:
**set1_ambient_1**	**set2_ambient_1**
Nitrogen-rich atmosphere:	Nitrogen-rich atmosphere:
**set1_N2_1**	**set2_N2_1**
**set1_N2_2**	**set2_N2_2**
	**set2_N2_3**
	**set2_N2_4**
	**set2_N2_5**

   Each of the nine folders contain the following subfolders and files.

   - **time_domain** contains raw time-domain data from the machine in **csv files** for each sample with the following header.

Time (ps)	Sample signal (nA)	Reference signal (nA)

   - **frequency_domain** contains transmittance and absorbance *vs* frequency data (computed and exported from data-processing.ipynb) in **csv files** for each sample with the following header

Frequency (THz)	Transmittance (arbitrary unit)	Absorbance (arbitrary unit)

     and refractive index and absorption coefficient spectral data for each sample in **refractive-index.csv** and **absorption-coefficient.csv**, respectively.
   - **peak_area_and_height** contains csv files of peak area and peak height values of the absorption spectra (in **absorption_spectra** subfolder) and absorption coefficient spectra (in **absorption_coefficient_spectra** subfolder) for each sample, using the following baseline correction methods from Rampy Python library
     - **peakvalues-poly.csv**: “poly” (polynomial fitting)
     - **peakvalues-als.csv**: “als” (automatic least squares fitting)
     - **peakvalues-arpls.csv**: “arpls” (asymmetrically reweighted penalized least squares smoothing)
     - **peakvalues-drpls.csv**: “drpls” (doubly reweighted penalized least squares smoothing; used for the quantification model in the paper)

     with the following header

Sample		Peak area		Peak height	
type	number	0.53 THz	1.37 THz	0.53 THz	1.37 THz

     (sample type: L = lactose standard; S = infant formula sample)

   c. **absorption_spectra_prediction.xlsx** contains results of the linear regression analysis (r^2, RMSE and predicted concentration values) using the absorption spectra, for each dataset and baseline correction method.
   absorption_coefficient_spectra_prediction.xlsx contains results of the linear regression analysis (r^2, RMSE and predicted concentration values) using the absorption coefficient spectra, for each dataset and baseline correction method.
   These results are from the analysis computed in **codes/regression.nb**.

2. **codes**
   a. Jupyter notebook **data-processing.ipynb** contains codes for the computation of the transmittance, absorbance, refractive index and absorption coefficient along with curve fitting and baseline correction for the computation of peak area and peak height
   b. Mathematica notebook **spectra-visualization.nb** contains codes for the visualization of the transmission, absorption, refractive index and absorption coefficient spectra
   c. Mathematica notebook **regression.nb** contains codes for the linear regression analysis and visualization of plots

DOI: 10.3390/molecules27155040

Table S1 Pellet mass and thickness of pure α-lactose monohydrate standards and infant formula samples for THz-TDS measurement

	Mass of pellet (mg)	Thickness of pellet (mm)
**Pure standard**		
Concentration of α-lactose monohydrate in % (w/w)		
0	99.6	0.770
0	100.6	0.977
1	94.3	0.934
3	97.2	0.982
5	92.9	0.908
10	92.9	0.866
15	96.0	0.885
20	93.7	0.828
49	96.1	0.712
80	99.3	0.625
100	93.5	0.553
100	92.7	0.551
**Infant formula sample**		
1	98.3	0.587
2	97.9	0.582
3	99.0	0.585

DOI: 10.3390/molecules27155040

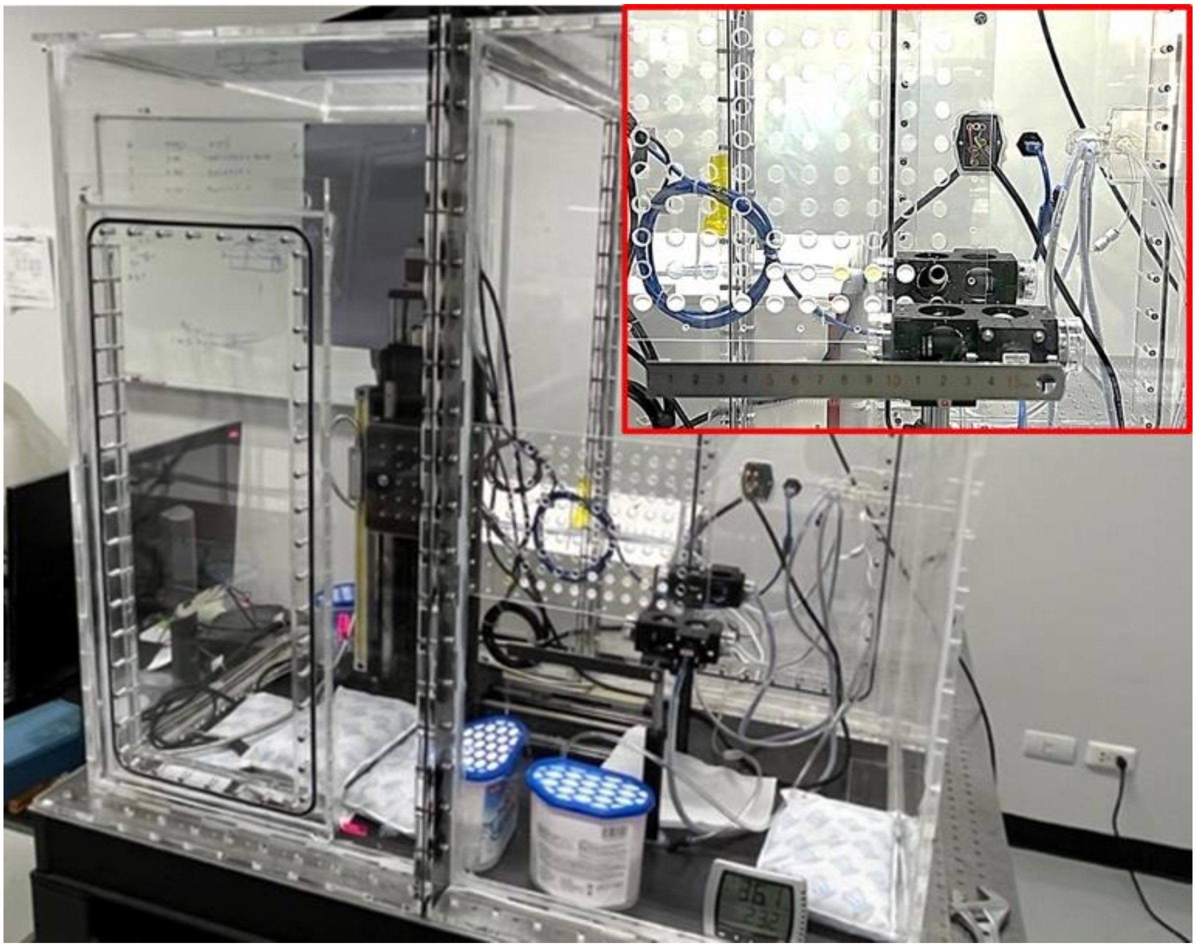

Figure S1 Custom-made THz-TDS system in this study at National Electronics and Computer Technology Center, Pathum Thani, Thailand (a zoom-in of the sample holder is framed in red)

DOI: 10.3390/molecules27155040

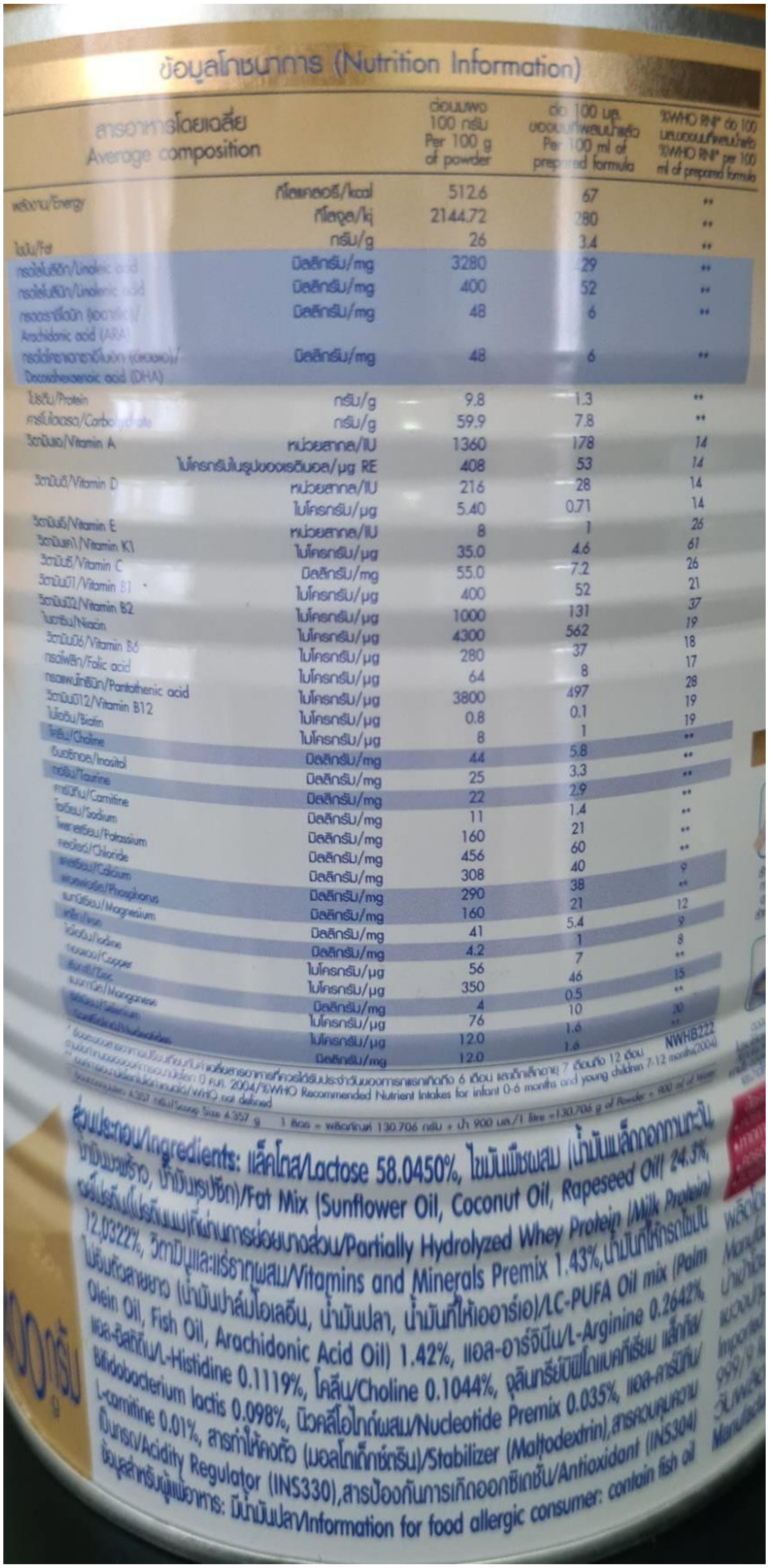

Figure S2 Product label of infant formula Sample 1
purchased from 7-Eleven, Thailand Science Park in late 2019

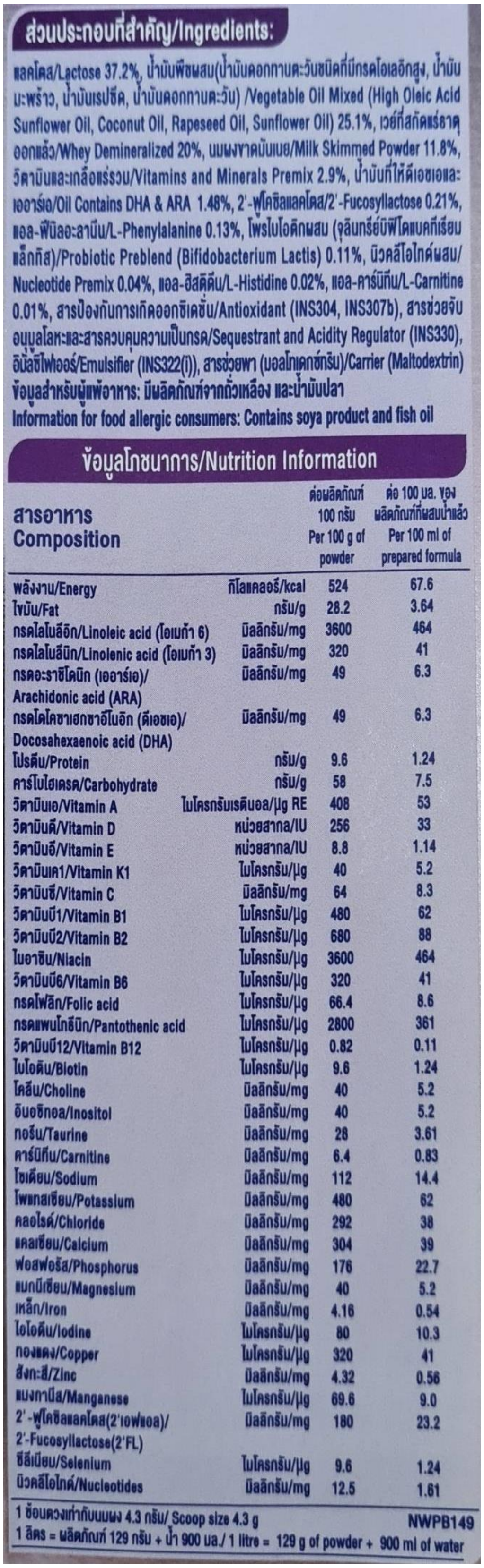

Figure S3 Product label of infant formula Sample 2 *purchased from Lotus's Khlong Luang on 11 March 2022*

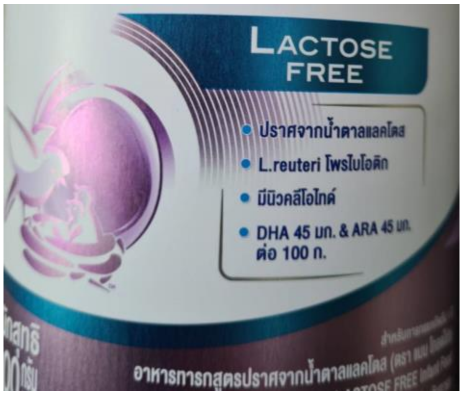

Figure S4 Product label of infant formula Sample 3 *purchased from Lotus's Khlong Luang on 11 March 2022*
